# Access to Functionalized Pyrenes, Peropyrenes, Terropyrenes, and Quarterropyrenes via Reductive Aromatization

**DOI:** 10.1002/anie.202100686

**Published:** 2021-05-07

**Authors:** Simon Werner, Tobias Vollgraff, Jörg Sundermeyer

**Affiliations:** ^1^ Fachbereich Chemie and Material Science Center (WZMW) Philipps-Universität Marburg Hans Meerwein Strasse 4 35032 Marburg Germany

**Keywords:** crystallography, cyclovoltammetry, fluorescence, organic dyes, reductive aromatization

## Abstract

Herein we report a versatile concept for the synthesis of fourfold functionalized, soluble pyrenes, peropyrenes, terropyrenes, and quarterropyrenes. They were obtained by a modular stepwise approach towards the rylene scaffold via Suzuki–Miyaura cross coupling, oxidative cyclodehydrogenation in the presence of caesium hydroxide under air, and finally zinc‐mediated reductive silylation. The silylated reaction products were characterized by X‐ray crystallography. The first example of a synthesized and crystallized quarterropyrene is presented and its oxidation reaction investigated. The functionalized ropyrenes were systematically characterized by means of UV/Vis–NIR and photoluminescence spectroscopy showing a bathochromic shift of 80 nm per naphthalene unit and a nearly linear increase of the extinction coefficients. Cyclic voltammograms and DFT calculations identify them as electron‐rich dyes and show a narrowing of the electrochemically determined HOMO–LUMO gap and lower oxidation potentials for the higher homologues.

## Graphical abstract

An approach for the modular synthesis of peropyrenes, terropyrenes, and quarterropyrenes via reductive aromatization and silylation key steps is presented. The functionalized members of the ropyrene family are characterized by X‐ray crystallography and ^13^C NMR spectroscopy. Optical spectroscopy and cyclic voltammetry are discussed in relation to DFT‐calculated frontier molecular orbital energies.

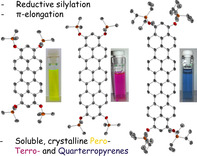

Polyaromatic hydrocarbons (PAHs) offer a huge variety of applications related to their unique (opto‐)electronic properties, for example in materials for organic electronics and photovoltaics.^[1]^ Studies elucidating structure–property relations gave rise to the synthesis of nanographenes with defined structures and tailored properties.^[2]^ Especially the edge structure and width determine their electronical properties (band structure).^[3]^ The family of poly‐perinaphthalenes (rylenes, see Figure 1 b), also known as 5‐armchair graphene nanoribbons (5‐AGNRs), is a broadly studied class of PAHs.^[4]^ Compared to other GNRs their simple structure, broad spectral light absorbance^[5]^ and singlet fission properties,^[6]^ as well as length‐dependent band gap sizes^[7]^ were motivation for intensive research efforts.^[8]^ With regard to molecular and soluble rylene compounds beyond the archetypical family of perylene diimides (PDIs, see Figure 1 a),^[9]^ higher homologue rylene diimides were synthesized by the group of Müllen using a modular approach of cross couplings starting from halogenated perylene or naphthalene monoimides and their corresponding boronic acid esters followed by oxidative cyclizations to form the rylenes.[^10^, ^11^, ^12^, ^13^, ^14^, ^15^] Thereby, rylene diimides with up to eight naphthalene units were formed, and also terrylene and quarterrylene were accessed.[^10^, ^11^, ^12^, ^13^, ^14^, ^15^, ^16^, ^17^]

**Figure 1:**
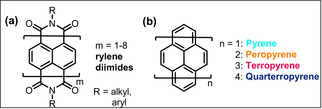
Chemical structures of a) the substance class of rylene diimides and b) pyrene and its higher homologues.

Another model system for molecular rylenes is peropyrene (Figure 1 b, *n*=2).^[18]^ Peropyrene is a potential candidate for singlet fission materials^[19]^ and effective synthesis strategies for substituted peropyrenes were developed during the past years.[^17^, ^20^, ^21^, ^22^] Terropyrenes, for example, the unsubstituted stem system^[23]^ or bent varieties for cyclophane syntheses,^[24]^ are rare in literature. All published strategies required multi‐step organic syntheses, especially for accessing the higher homologues, terropyrenes.[^21^, ^22^, ^25^, ^26^] Key of the synthesis route reported by Chalifoux and co‐workers are π‐extentions of aromatic systems using two‐ or tetrafold alkynylated precursors in acid‐mediated[^21^, ^22^, ^27^] or InCl_3_‐catalyzed benzannulations.^[25]^

Inspired by the modular construction principle of higher rylene diimide dyes[^11^, ^13^, ^15^] and the novel reductive functionalization approaches for naphthalene diimide (NTCDI)^[28]^ and perylene diimide (PTCDI)[^29^, ^30^] in our group and in the group of Miyake, we intended to reductively access higher homologue ropyrenes. The precursors **2**, **3**, **11**, and **12** of the final peropyrenes, terropyrenes, and quarterropyrenes are synthesized in analogy to the modular synthesis of soluble, liquid crystalline dihydroxy‐ropyrene‐quinones reported by Buffet and Bock (Scheme 1).^[31]^ Peropyrenequinone **2** was directly reduced and silylated, reacting with Zn as reducing agent in the presence of trimethylsilyl chloride to the orange air sensitive peropyrene silylether **4**. Zinc turned out to be the best reducing agent. It is readily available, non‐toxic, and processable even under non‐inert conditions. For reductive silylation of the smaller dihydroxy pyrenequinone **2** we could introduce four triisopropylsilyl groups, leading to highly soluble pyrene‐tetrasilyl ether **5** in rather poor yield. Next, we turned our attention to the application of a similar reduction protocol to obtain the higher homologues of **2**, terropyrenequinone **11** and quarterropyrenequinone **12**.

**Scheme 1:**
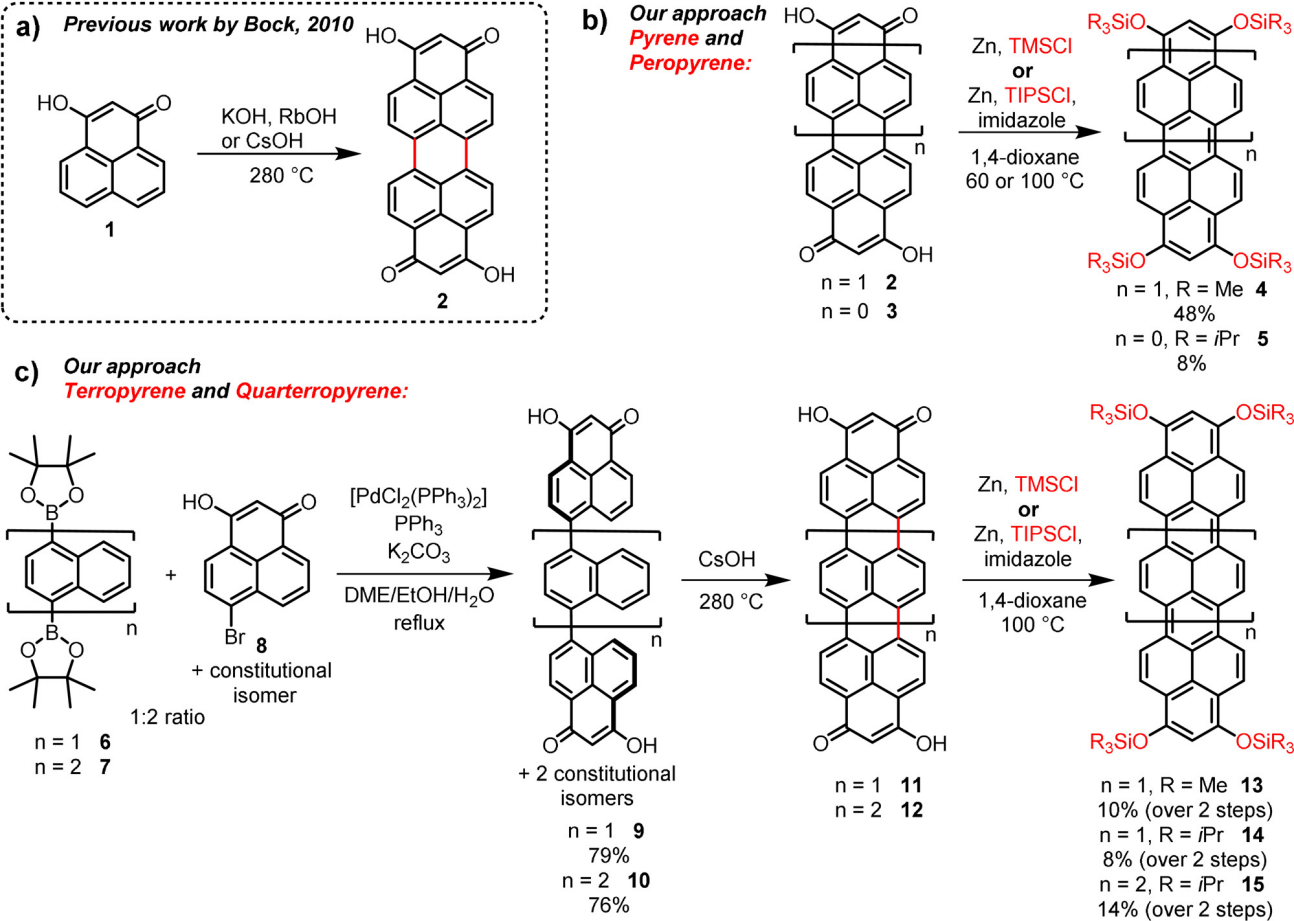
a) Previously reported synthesis of dihydroxy‐peropyrenequinone (**2**); b) our approaches for the synthesis of peropyrene‐silylether **4** and pyrene‐silylether **5** by reductive functionalization; c) the higher homologue terropyrene‐silylethers **13** and **14** and quarterropyrene‐silylether **15** by modular approaches.

**11** and **12** were synthesized in a facile two‐step protocol starting with bis‐boronic acid pinacol esters **6** ^[32]^ and **7** ^[33]^ together with peri‐brominated hydroxyphenalenone **8** under standard Suzuki–Miyaura coupling conditions in a 1:2 ratio. The resulting ter‐ and quarternaphthyls **9** and **10** could be isolated as inseparable isomer mixture (see SI) by a simple precipitation due to protonation with diluted acetic acid and subsequent washing with methanol. Non‐planar **9** and **10** were heated in a CsOH melt for three hours to 280 °C in a Ni‐crucible on air until the gas evolution ceased. We chose CsOH as base because of the improved intercalation and interaction capabilities of caesium cations with intermediately formed aromatic anions. **11** and **12** were isolated as insoluble dark blue solids that were directly used for further transformations. Surprisingly, **11** and **12** reacted under similar conditions as **2** with zinc and trialkylsilyl chlorides in dioxane despite their insolubility at 100 °C. The resulting silylated products were soluble since their capability of π–π stacking is reduced due to the sterically demanding silyl groups. Hence, a similar terropyrene‐trimethylsilylether **13**, as a higher homologue to **4**, could be isolated as purple‐red solid. After activating triisopropylsilyl chloride with imidazole, a reductive silylation of **11** and even **12** yielded the air stable terropyrene‐triisopropyl silylether **14** and royal blue colored quarterropyrene‐triisopropylether **15** with superior solubility, high purity, however low yields. Both **14** and **15** could be isolated by column chromatography with dichloromethane. We further synthesized a congener of **15** with *n*‐butyl groups in both terminal positions and TMS groups (**S4**) in order to improve the solubility, but the solubilizing effect of non‐bulky butyl groups was not significantly more effective. Unfortunately, all attempts to synthesize a higher homologue penterropyrene were not successful so far, since the corresponding penterropyrene‐quinone was too insoluble for further transformations. The good solubility of our silylated rylenes **4**, **13**, and **15** in chlorinated solvents made it possible to obtain X‐ray diffractive single crystals via slow gas phase diffusion of *n*‐pentane (Figure 2). Trimethylsilyl ethers **4** and **13** crystallize in the monoclinic space group *P*2_1_/*c* with π–π stacking pairs (*d*_π–π_=3.30 Å (**4**) and 3.64 Å (**13**)) of molecules oriented in a herringbone arrangement for **13**. Quarterropyrene **15** crystallizes in the monoclinic spacegroup *P*2_1_/*n* being the first investigated quarteropyrene in the crystalline state to the best of our knowledge. Its bulkier TIPS groups do not prevent the face‐to‐face π–π stacking interactions in the solid state (*d*_π–π_=3.36 Å) but induce a slight bending of the rylene backbone in comparison to the trimethylsilyl‐substituted homologues **4** and **13** and lead to a transversal intermolecular displacement in order to minimize steric repulsions.

**Figure 2:**
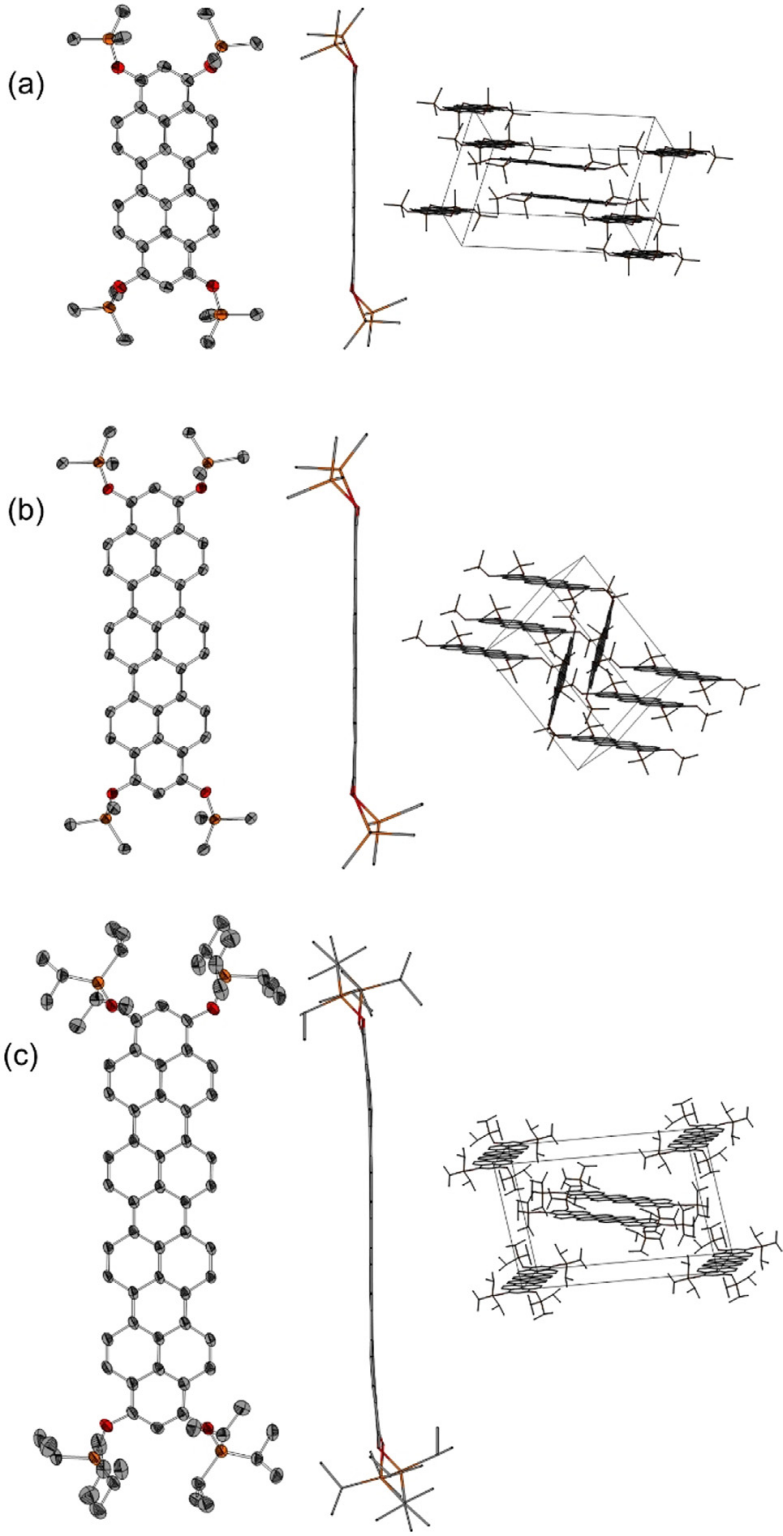
Solid state structures (left: front view, center: side view, and right: packing structure) of a) **4**; b) **13**, and c) **15**. Hydrogen atoms are omitted for clarity and thermal ellipsoids are shown at the 50 % probability level.^[38]^

The (opto‐)electronical properties of the rylenes **4**, **5**, **13**, **14**, and **15** were investigated by UV/Vis–NIR and photoluminescence spectroscopy as well as cyclovoltammetry. The optical spectra and cyclovoltammogramms of both quarterropyrene silylethers **13** and **14** were almost identical (see Figure S7). Typical for rylene dyes,[^11^, ^13^] the absorption spectra are dominated by the lowest‐energy S_1_←S_0_‐transition. The absorption maxima (*λ*_max_) and extinction coefficients (*ϵ*) increase nearly linearly with each additional naphthalene unit [a bathochromic shift of about 80 nm and an increase of the extinction coefficient from *λ*_max_=390 nm (*ϵ*=24 500 L mol^−1^ s^−1^) for **5** to *λ*_max_=634 nm (*ϵ*=128 200 L mol^−1^ s^−1^) for **15** (Figure 3 a)] with extinction coefficients in slightly smaller magnitudes compared to the related rylene diimides.[^11^, ^13^] All four compounds showed nearly no concentration‐dependent absorption maxima (see Figure S2–S6). TD‐DFT (see Figure S13) supports the trend also regarding the oscillator strengths of the *λ*_max_ transitions. The mirrored emission spectra reveal a similar trend with Stokes shifts of around 15 nm. **4**, **13**, and **15** show strong vibronic progressions indicating, together with the narrow Stokes shift, small reorganization energies.^[34]^ A broadening of the bands can be observed for the higher homologues. Measured fluorescence quantum yields (*Φ*_PL_, see SI) were only moderately high in case of **4** (0.30), lower for **13** (0.08) and **14** (0.06), and undetectably low in **4** and **15** with bulky rotating TIPS groups most likely deactivating the fluorescence through reorganization energy loss. The predictable evolution of optical properties can also be rationalized with the Kohn–Sham molecular orbitals of the HOMO and LUMO for **4**, **5**, **13**, and **15**, being of similar modular symmetry (Figure 3 d). The frontier molecular orbital energies were experimentally assigned using cyclic voltammetry (Figure 3 c). The electron‐rich dyes **4**, **5**, **14**, and **15** show two pronounced and quasi‐reversible oxidation potential waves, whereas only **14** shows one and **15** shows two not fully reversible reduction potential waves within the electrochemical window of dichloromethane. In comparison to reported arylated pero‐ and terropyrenes, **5** and **14** show lower oxidation potentials and are therefore more electron‐rich.^[25]^ Additionally, a narrowing of the electrochemically determined HOMO–LUMO gap with the growing π‐system could be observed. The experimental HOMO energies were referenced to the vacuum energy level of the ferrocene/ferrocenium redox couple (−4.8 eV,^[35]^ see Figure S8–S12) and the LUMO energy was accessed with the help of the optical HOMO–LUMO gap (determined from the intersection wavelength of normalized UV/Vis and PL spectra^[36]^). Both experiment and theory show an energy gap (E_g_) narrowing from E_g_=3.02 eV (theory 3.48 eV) for pyrene **5** to 1.93 eV (theory 1.96 eV) to **15**, respectively (see Table 1). This is indicated by higher HOMO and lower LUMO energies (from experimentally −5.01 eV (HOMO) and −2.04 eV (LUMO) of **5** to −4.62 eV (HOMO) and −2.73 eV (LUMO) for **15**), with theoretical values supporting the observed trends. In comparison with arylated ropyrenes, silylether substitution leads to higher HOMO levels.^[25]^

**Figure 3:**
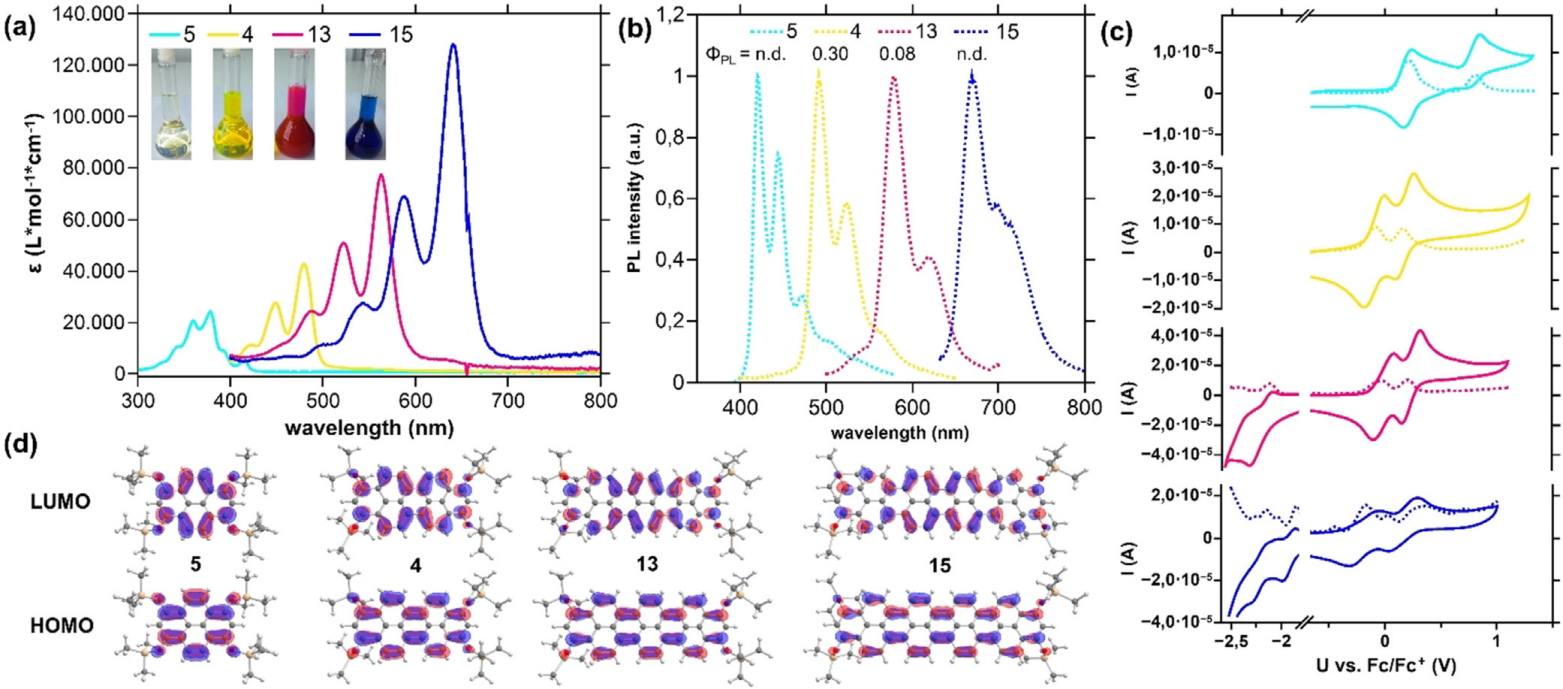
a) UV/Vis–NIR spectra of **4**, **5**, **13**, and **15** (*c*≈1×10^−5^ m) in CH_2_Cl_2_ and photographs of the corresponding solutions (inset). b) Normalized emission spectra of **4**, **5**, **13**, and **15** in CH_2_Cl_2_ (*c*≈1×10^−7^ m, *λ*_ex._=350 nm). c) Cyclic voltammograms of **4**, **5**, **14**, and **15** (measured in CH_2_Cl_2_, 0.1 m *n*‐Bu_4_NPF_6_, 100 mV s^−1^ scan rate, glassy carbon working electrode, platinum reference electrode) and corresponding differential pulse voltammograms (DPV, dashed lines, 10 mV s^−1^ scan rate). d) HOMO and LUMO of **5**, **4**, **13**, and **15** calculated by DFT (TIPS groups simplified by TMS, B3LYP, def‐2/TZVPP, isoval. 0.03 a.u.).

**Table 1:** Summary of experimentally and theoretically determined HOMO and LUMO energies of **4**, **5**, **14**, and **15**.

	*E*_HOMO,exp._ [eV]^[a]^	*E*_LUMO,exp._ [E_g_] [eV]^[b]^	*E*_HOMO,theo._ [eV]^[c]^	*E*_LUMO,theo._ [E_g_] [eV]^[c]^
**5**	−5.01	−2.04 [3.02]	−4.59	−1.11 [3.48]
**4**	−4.71	−2.16 [2.55]	−4.54	−1.85 [2.69]
**14**	−4.80	−2.66 [2.14]	−4.32	−2.02 [2.20]
**15**	−4.62	−2.73 [1.89]	−4.27	−2.33 [1.94]

[a] Electrochemically determined oxidation and reduction potentials referring to Fc/Fc^+^ as internal standard (*E*_HOMO_(fc)=−4.8 eV): *E*_HOMO,exp_=−4.8 eV−*E*_1/2,ox1_. [b] Determined HOMO and LUMO energies based on the optical HOMO–LUMO energy gap [E_g_]: *E*_LUMO,exp_=*E*_HOMO,exp_+E_g,opt_. [c] Calculated HOMO and LUMO energies, level of theory: def2‐TZVPP/B3LYP, for carthesian coordinates (XYZ) of optimized geometries see Table S1–S4.

The high HOMO value of **15** and also the existence of a non‐zero baseline in the NIR region of the UV/Vis spectrum gave rise to the assumption that minor amounts of the oxidation product **[15]^+^** are present after column chromatography with dichloromethane, also observed for other electron‐rich PAHs and resulting from traces of acid in the presence of air.^[37]^ In order to study the oxidation product of **15**, we added the oxidant nitrosyl tetrafluoroborate (NOBF_4_) in a UV/Vis–NIR titration experiment. Upon addition of excess (10 equiv) of NOBF_4_ a gradual color change from blue to turquoise green and broad NIR absorption bands appearing at 820 and 1050 nm (Figure 4) were observed. Addition of more than 10 equiv NOBF_4_ had no further effect. Using ^1^H NMR, the formation of a paramagnetic compound could be observed by the absence of distinct signals. TD‐DFT calculations (see SI, Figure S15) of **[15]^+^** describe the novel band at 1050 nm as SOMO–LUMO transition.

**Figure 4:**
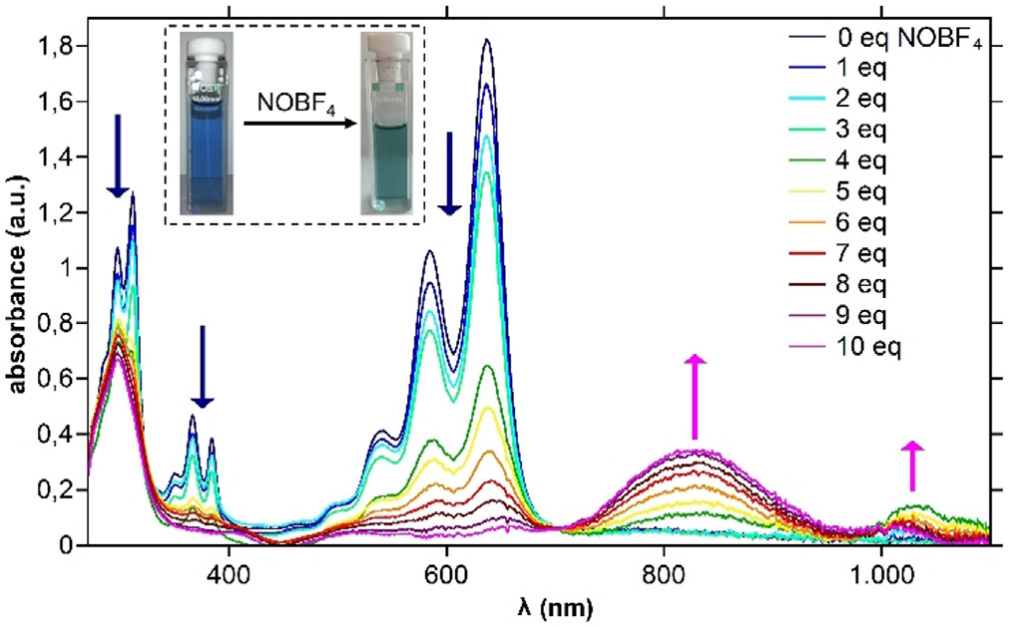
Changes in the UV/Vis–NIR absorption spectrum of **15** during titration with NOBF_4_ in CH_2_Cl_2_/acetonitrile. Inset: Color change after addition of 10 equiv of NOBF_4_.

In summary, we report a facile and efficient access to the higher homologues of peropyrene by a modular and scalable synthetic bottom‐up approach with a reductive aromatization by Zn/R_3_SiCl as a key step towards functionalized, soluble, and easily isolable electron‐rich rylene silylethers. Supported by comprehensive experimental spectroscopy and spectrometry, the influence of the enlargement by naphthalene units on the HOMO–LUMO gap was rationalized by spectroscopy, CV, and DFT calculations. As OSiR_3_ groups can be converted to OTf functionalities for further transformations,^[30]^ we are convinced that our new synthesis strategy allows an efficient general synthetic entry into novel functionalized PAHs for high‐performance organic electronic materials.

Supporting Information available: Additional experimental details, NMR spectra, CV/DPV measurements, UV/Vis and TD‐DFT spectroscopy, cartesian coordinates of calculated structures (XYZ), and XRD data. The CIF files of the presented structures are provided.

## Conflict of interest

The authors declare no conflict of interest.

## Supporting information

As a service to our authors and readers, this journal provides supporting information supplied by the authors. Such materials are peer reviewed and may be re‐organized for online delivery, but are not copy‐edited or typeset. Technical support issues arising from supporting information (other than missing files) should be addressed to the authors.

Supplementary
