# Supplementary material for: Access to Functionalized Pyrenes, Peropyrenes, Terropyrenes, and Quarterropyrenes via Reductive Aromatization
Source: Angew Chem Int Ed Engl. 2021 May 7;60(24):13631–5. doi: 10.1002/anie.202100686 (PMC8252597; doi:10.1002/anie.202100686)
Supplement: Supplementary file 1 — Supplementary [file ANIE-60-13631-s001.pdf]

Angewandte Chemie
Eine Zeitschrift der Gesellschaft Deutscher Chemiker

Supporting Information

Access to Functionalized Pyrenes, Peropyrenes, Terropyrenes, and Quarterropyrenes via Reductive Aromatization

*Simon Werner, Tobias Vollgraff, and Jörg Sundermeyer**

anie_202100686_sm_miscellaneous_information.pdf

Contents

Materials and Methods **4**

Additional experimental data for synthesis **5**

*Synthesis of a butyl-substituted quarteropyrene-trimethylsilyl ether (**S4**)* *5*

Attempts for the reductive aromatization towards penteropyrenes *6*

Concentration-dependent UV-Vis-NIR spectra **6**

Determination of fluorescence quantum yields **8**

Comparison of TMS vs. TIPS substituted terropyrene **9**

Cyclovoltammetry raw data and spectra referenced on ferrocene **9**

Density functional theory (DFT) **11**

Optimized geometries (XYZ-files) *11*

TD-DFT calculations *19*

Single crystal X-ray structures **21**

Crystal Data *21*

Molecular structures of the title compounds *22*

1,3,8,10-Tetrakis((trimethylsilyl)oxy)dibenzo-[*cd*,*lm*]-perylene (4) **22**

1,3,10,12-tetrakis((trimethylsilyl)oxy)benzo[rst]dinaphtho[8,1,2-*cde*:2',1',8'-*klm*]pentaphene (13) **25**

1,3,10,12-tetrakis((triisopropylsilyl)oxy)benzo[*rst*]dinaphtho[8,1,2-*cde*:2',1',8'-*klm*]pentaphene (15) **27**

Experimental **30**

*1,4-bis(4,4,5,5-tetramethyl-1,3,2-dioxaborolan-2-yl)naphthalene (**6**)* *30*

*4,4'-dibromo-1,1'-binaphthyl (**S10**)* *30*

*4,4'-bis(4,4,5,5-tetramethyl-1,3,2-dioxaborolan-2-yl)-1,1'-binaphthalene (**7**)* *31*

*4,4''-dibromo-1,1':4',1''-ternaphthalene (**S5**)* *31*

*4,4''-bis(4,4,5,5-tetramethyl-1,3,2-dioxaborolan-2-yl)-1,1':4',1''-ternaphthalene (**S6**)* *31*

*6-bromo-3-hydroxy-1H-phenalen-1-one (**8**)* *32*

*6-bromo-2-butyl-3-hydroxy-1H-phenalen-1-one (**S1**)* *32*

*3-hydroxy-7-(4-(3-hydroxy-1-oxo-1H-phenalen-6-yl)naphthalen-1-yl)-1H-phenalen-1-one (**9**)* *33*

*3-hydroxy-7-(4'-(3-hydroxy-1-oxo-1H-phenalen-6-yl)-[1,1'-binaphthalen]-4-yl)-1H-phenalen-1-one (**10**)* *33*

*2-butyl-7-(4'-(2-butyl-3-hydroxy-1-oxo-1H-phenalen-6-yl)-[1,1'-binaphthalen]-4-yl)-3-hydroxy-1H-phenalen-1-one (**S2**)* *34*

*2-butyl-7-(4''-(2-butyl-3-hydroxy-1-oxo-1H-phenalen-6-yl)-[1,1':4',1''-ternaphthalen]-4-yl)-3-hydroxy-1H-phenalen-1-one (**S7**)* *34*

*3,8-dihydroxypyrene-1,6-dione (**3**)* *35*

3,10-dihydroxydibenzo[cd,lm]perylene-1,8-dione (2) *35*

*3,12-dihydroxybenzo[rst]dinaphtho[8,1,2-cde:2',1',8'-klm]pentaphene-1,10-dione (**11**)* *35*

*3,14-dihydroxybenzo[rst]benzo[12,1]tetrapheno[7,8,9-cde]naphtho[2,1,8-klm]pentaphene-1,12-dione (**12**)* *36*

*2,13-dibutyl-3,14-dihydroxybenzo[rst]benzo[12,1]tetrapheno[7,8,9-cde]naphtho[2,1,8-klm]pentaphene-1,12-dione (**S3**)* *36*

*2,13-dibutyl-3,14-dihydroxybenzo[rst]benzo[12,1]tetrapheno[7,8,9-cde]naphtho[2,1,8-klm]pentaphene-1,12-dione (**S8**)* *36*

*1,3,6,8-tetrakis((triisopropylsilyl)oxy)pyrene (**5**)* *37*

*1,3,8,10-tetrakis((trimethylsilyl)oxydibenzo[cd,lm]-perylene (**4**)* *37*

*1,3,10,12-tetrakis((trimethylsilyl)oxy)benzo[rst]dinaphtho[8,1,2-cde:2',1',8'-klm]pentaphene (**13**)* *37*

*1,3,10,12-tetrakis((triisopropylsilyl)oxy)benzo[rst]dinaphtho[8,1,2-cde:2',1',8'-klm]pentaphene (**14**)* *38*

*((2,13-dibutylbenzo[rst]benzo[12,1]tetrapheno[7,8,9-cde]naphtho[2,1,8-klm]pentaphene-1,3,12,14-tetrayl)tetrakis(oxy))-tetrakis(trimethylsilane) (**S4**)* *38*

*1,3,10,12-tetrakis((triisopropylsilyl)oxy)benzo[rst]dinaphtho[8,1,2-cde:2',1',8'-klm]pentaphene (**15**)* *39*

NMR spectra 40

*1,4-bis(4,4,5,5-tetramethyl-1,3,2-dioxaborolan-2-yl)naphthalene (**6**)* *40*

*4,4'-dibromo-1,1'-binaphthyl (**S10**)* *41*

*4,4'-bis(4,4,5,5-tetramethyl-1,3,2-dioxaborolan-2-yl)-1,1'-binaphthalene (**7**)* *42*

*4,4''-dibromo-1,1':4',1''-ternaphthalene (**S5**)* *43*

*4,4''-bis(4,4,5,5-tetramethyl-1,3,2-dioxaborolan-2-yl)-1,1':4',1''-ternaphthalene (**S6**)* *44*

*6-bromo-3-hydroxy-1H-phenalen-1-one (**8**)* *45*

*6-bromo-2-butyl-3-hydroxy-1H-phenalen-1-one (**S1**)* *46*

*3-hydroxy-7-(4-(3-hydroxy-1-oxo-1H-phenalen-6-yl)naphthalen-1-yl)-1H-phenalen-1-one (**9**)* *47*

*3-hydroxy-7-(4'-(3-hydroxy-1-oxo-1H-phenalen-6-yl)-[1,1'-binaphthalen]-4-yl)-1H-phenalen-1-one (**10**)* *48*

*2-butyl-7-(4'-(2-butyl-3-hydroxy-1-oxo-1H-phenalen-6-yl)-[1,1'-binaphthalen]-4-yl)-3-hydroxy-1H-phenalen-1-one (**S2**)* *48*

*2-butyl-7-(4'-(2-butyl-3-hydroxy-1-oxo-1H-phenalen-6-yl)-[1,1'-binaphthalen]-4-yl)-3-hydroxy-1H-phenalen-1-one (**S7**)* *49*

*1,3,6,8-tetrakis((triisopropylsilyl)oxy)pyrene (**5**)* *49*

*1,3,8,10-tetrakis((trimethylsilyl)oxydibenzo[cd,lm]-perylene (**4**)* *50*

*1,3,10,12-tetrakis((trimethylsilyl)oxy)benzo[rst]dinaphtho[8,1,2-cde:2',1',8'-klm]pentaphene (**13**)* *51*

*1,3,10,12-tetrakis((triisopropylsilyl)oxy)benzo[rst]dinaphtho[8,1,2-cde:2',1',8'-klm]pentaphene (**14**)* *52*

*((2,13-dibutylbenzo[rst]benzo[12,1]tetrapheno[7,8,9-cde]naphtho[2,1,8-klm]pentaphene-1,3,12,14-tetrayl)tetrakis(oxy))-tetrakis(trimethylsilane) (**S4**)* *53*

*1,3,10,12-tetrakis((triisopropylsilyl)oxy)benzo[rst]dinaphtho[8,1,2-cde:2',1',8'-klm]pentaphene (**15**)* *54*

APCI(-) Mass spectra of Suzuki coupling products 56

Reverse Phase HPLC traces of Suzuki coupling products 58

References 59

Materials and Methods

All preparative operations were conducted by using standard Schlenk techniques and solvents were dried according to common procedures[1] and passed through columns of aluminium oxide, 3 Å molecular sieves and R3-11G-catalyst (BASF) or stored over molecular sieves (3 Å or 4 Å).
The data collection for the single-crystal structure determination was performed on a Stoe Stadivari by the X-ray service of Fachbereich Chemie, Universität Marburg. Information concerning the used hardware, and software used for data collection, cell refinement and data reduction as well as structure refinement can be reviewed in the electronic supplement tables and CCDC 2055267 – 2055269. After solution (SHELXT)[2] and refinement process (SHELXL 2017/1)[3] the data were validated by using Platon.[4] All graphic representations were created with Diamond 4.[5]
^{1}H and proton decoupled ^{13}C-NMR spectra were recorded in automation or by the service department of Fachbereich Chemie with a Bruker Avance II 300 spectrometer, a Bruker Avance II HD 300 or Avance III 500 spectrometer. All spectra were recorded at ambient temperature, if not mentioned otherwise. ^{1}H and ^{13}C NMR spectra were calibrated using residual proton signals of the solvent (CD_2Cl_2: δ_H = 5.32 ppm, δ_C = 53.84 ppm, $CDCl_3$: δ_H = 7.26 ppm, δ_C = 77.16 ppm). Multiplicity is abbreviated as follows: s (singlet), d (doublet), t (triplet), q (quartet), qt (quintet), m (multiplet), br. HR-APCI mass spectra were acquired with a LTQ-FT Ultra mass spectrometer (Thermo Fischer Scientific). The resolution was set to 100.000. HR-EI mass spectra were acquired with an AccuTOF GCv 4G (JEOL) Time of Flight (TOF) mass spectrometer. An internal or external standard was used for drift time correction. The LIFDI ion source and FD-emitters were purchased from Linden ChroMasSpec GmbH (Bremen, Germany). IR spectra were recorded in a glovebox on a Bruker Alpha ATR-FT-IR spectrometer.
Reverse Phase high-performance liquid chromatography coupled with mass spectrometry (HPLCMS) was used for estimation of product purity of Suzuki coupling products **9**, **10**, **S2** and **S7**. The spectra were recorded on a 1260 Infinity (Agilent) HPLC-system coupled with an Expression S CMS (Advion) mass spectrometer.
Absorption spectra were recorded with a Avance AvaSpect 2048 UV/Vis/NIR spectrophotometer in 10 mm cuvettes in dichloromethane in a nitrogen filled glovebox (Labmaster 130, mBraun) with a scan rate of 600 nm/min. Emission spectra were recorded with a Varian Cary Eclipse Spectrophotometer in 10 nm cuvettes in dichloromethane with a scan rate of 600 nm/min.
Fluorescence quantum yields (Φ_{PL}) were recorded by dilution method using a fluorescein solution (0.1 M in aqueous NaOH, Φ_{flu} = 0.95)[6] as reference. Both, the samples and the reference were measured at low concentrations in order to ensure a linear relationship between the intensity of emitted light and the concentration of the absorbing/emitting species. The quantum yields of the samples (Φ_s) were determined by the following equation 1.[6]

$$\Phi_{flu}=\frac{grad(s)}{grad(flu)}*\frac{n^2(s)}{n^2(flu)}*\Phi_{flu}$$

In equation 1, *grad* is the slope of the “emission versus absorbance” plot of the amples (s) and the standard fluorescein (flu). n is the refractive index of the used solvents.
Cyclic voltammetry (CV) and differential pulse voltammetry (DPV) measurements were carried out on a rhd instruments TSC 1600 closed electrochemical workstation (working electrode: glassy carbon; counter electrode: platinum crucible; reference electrode: platinum wire (pseudo reference electrode under nitrogen atmosphere in a glovebox (Labmaster 130, mBraun). The samples were measured in dichloromethane (concentration: 5 mmol/L) and calibrated using ferrocene as internal standard after measurements. Dichloromethane was filtered through an aluminum oxide pad prior to use. Tetrabutylammonium hexafluorophosphate ($TBAPF_6$; >99.0 %) was used as electrolyte for electrochemical analysis. The measurements were carried out at a concentration of 100 mmol/L of electrolyte.

Additional experimental data for synthesis

*Synthesis of a butyl-substituted quarteropyrene-trimethylsilyl ether (**S4**)*

As first attempt for the synthesis of a soluble quarteropyrene tetra-silylether, we synthesized a quarteropyrene trimethylsilylether (**S4**), terminal functionalized with *n*-butyl groups (see scheme S1). The synthesis route is analogous as discussed in the main text. As a first step, we reacted binaphthyl-bisboronic acid pinacolester with 6-bromo-2-butyl-3-hydroxy-1*H*-phenalen-1-one (**S1**) to form quarternaphthyl **S2** in moderate yields (37%). **S2** shows a slightly improved solubility in comparison with its non-butylated congener **10**, so a purification by column chromatography was possible. Nevertheless, the cyclodehydrogenated reaction product **S3** from the CsOH-melt air oxidation reaction of **S2**, was as insoluble as the non-butylated counterpart **12**. In a final step, **S3** was reacted with Zn and TMSCl to form silylether **S4**, proceeding with a yield (14% over 2 steps) comparable to the in the main text discussed reductive silylations of terropyrenequinone **11** and quarterropyrenequinone **12**. The solubility of **S4** in organic solvents as chloroform is moderate, so the acquisition of a proton NMR spectra was possible at elevated temperature (50 °C), but no 13C NMR or single crystals for XRD measurements could be obtained. It can be concluded that **S4** is less soluble than quarteropyrene triisopropylsilylether **15**, meaning that the TIPS groups prevent the quarteropyrene core much better from π-π-stacking interactions than butyl groups in combination with a TMS-substitution.

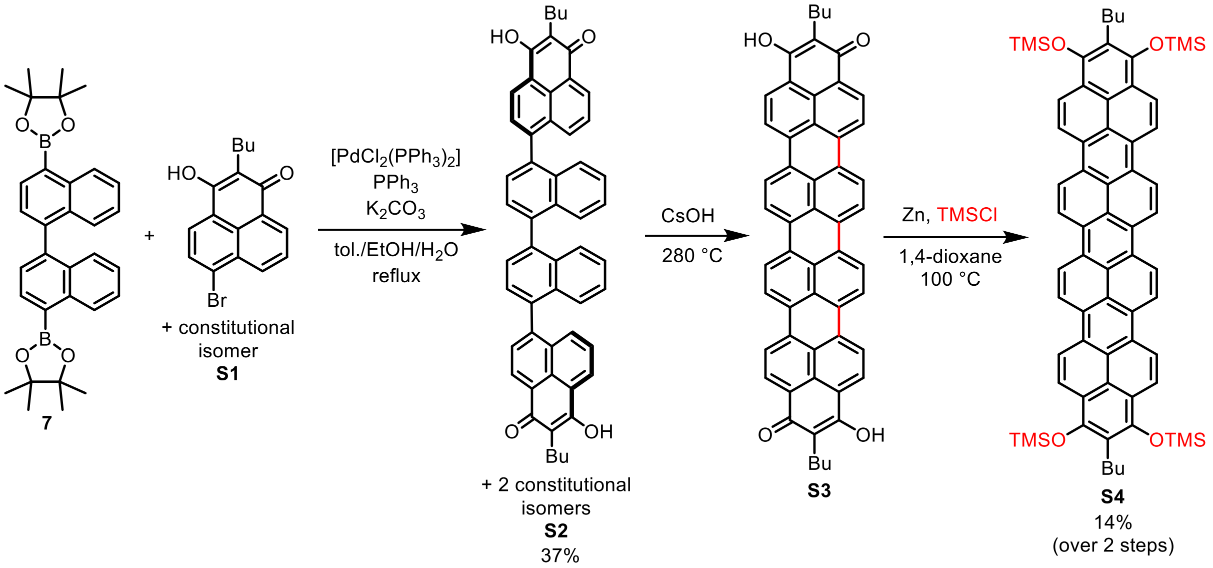

Scheme S1. Synthetic route towards butyl-substituted quarterropyrene trimethylsilyl ether **S4**.

S4 shows an absorption maximum at 634 nm in DCM with a nearly equal UV-Vis-NIR spectrum in comparison to **15**. The broadened bands in comparison to lighter homologue trimethylsilyl ethers indicate the weak solubility of **S4**. The cyclic voltammogram in DCM shows two reduction waves (-2.00 V and -1.82 V vs. Fc/Fc^+) and two oxidation waves (0.24 V and 1.24 V vs. Fc/Fc^+), none of them being fully reversible (figure S1).

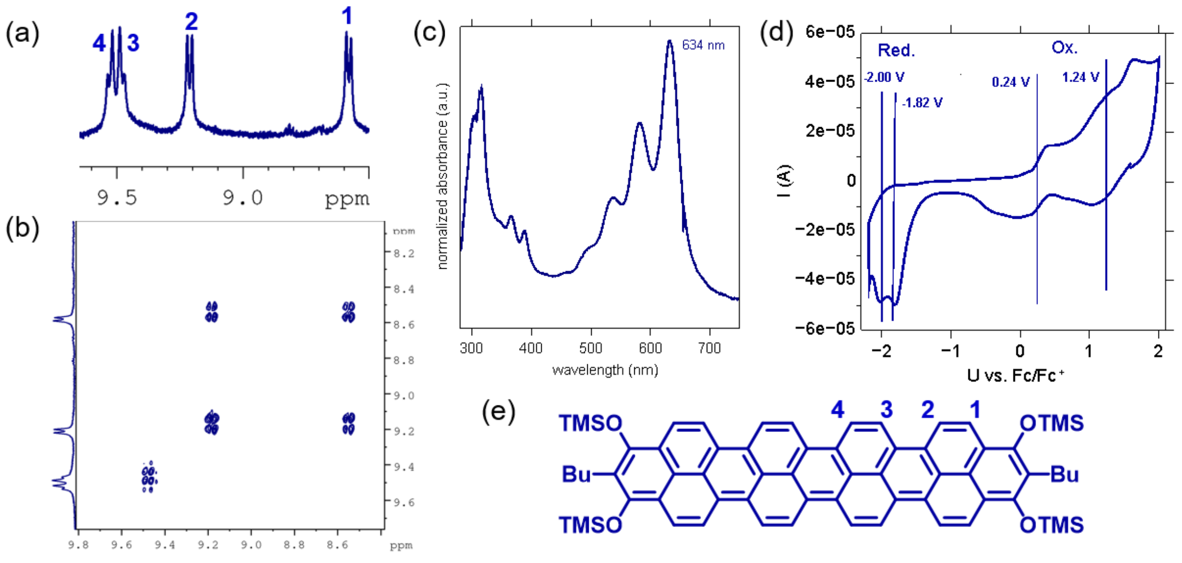

Figure S1. (a) Aromatic region of ^{1}H NMR spectrum of **S4** in $CDCl_3$ (323 K, 500 MHz); (b) COSY-NMR (500 MHz, $CDCl_3$) of the aromatic region of **S4**; (c) UV-Vis spectrum of **S4** measured in DCM; (d) cyclic voltammogram of **S4** in DCM (Scan rate 50 mV/s, electrolyte: nBu_4NPF_6, vs. Fc/Fc^+); (e) chemical structure of **S4** with assigned aromatic protons from NMR.

Attempts for the reductive aromatization towards penteropyrenes

The strategy of terminal butyl functionalization was also applied in the case of the synthesis of a penteropyrene, the higher homologue of quarteropyrene (Scheme S2). Therefore, ternaphthyl-bis boronic acid pinacolester **S6** as central building block was synthesized by a Suzuki Miyaura cross coupling of 1,4-dibromonaphthalene (**S4**) in excess (3 eq) with naphthalene bis boronic acid pinacolester **6**, leading to dibromo-ternaphthyl **S5** in moderate yield (40%). **S5** was subsequently converted to **S6** by Miyaura borylation. **S6** could be analogously to the binaphthyl congener **7** reacted with hydroxy-phenalenone **S1** to the non-planar pentanaphthyl **S7**, which could be isolated in good yields (70%). The following cyclodehydrogenative oxidation led to the black powder **S8**. Suffering even more from insolubility than quarterropyrenequinone **S3**, the penterropyrenequinone-analogue **S8** did not react in silylation reactions, even treatment with Zn, TIPSCl in the presence of imidazole under reflux in dioxane did not lead to a detectable product formation by mass spectrometry. The only evidence for traces of conversion of **S8** was a slightly dark green colored DCM solution after extraction the residue and filtration, eventually indicating a red shifted absorption maximum with respect to the dark blue colored lighter homologue quarteropyrene.

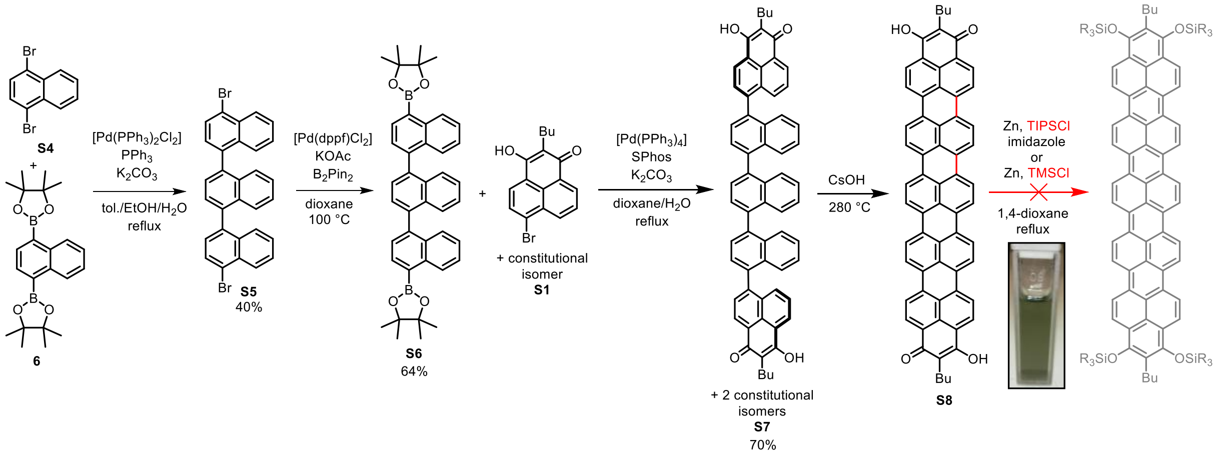

Scheme S2. Attempted synthetical route towards penterropyrenes**.**

Concentration-dependent UV-Vis-NIR spectra

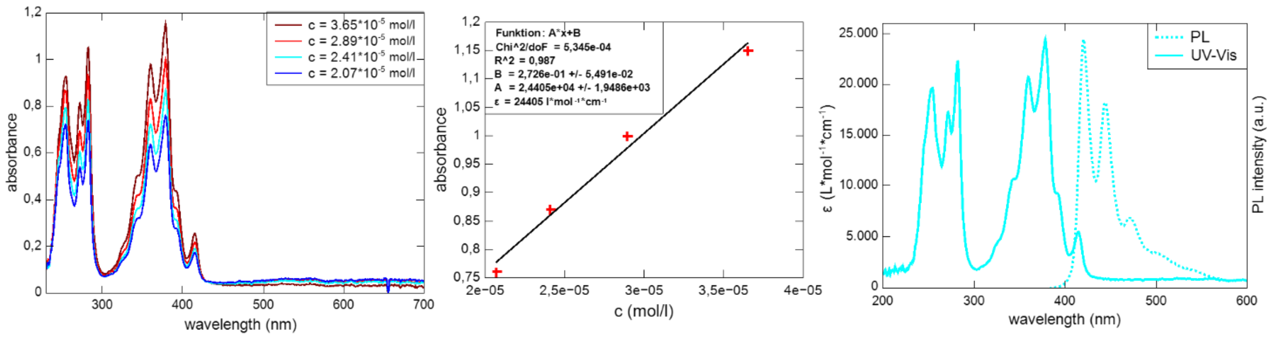

Figure S2. Left: UV-Vis-NIR spectra of pyrene **5** at four different concentration, recorded in CH_2Cl_2. Center: plot of the absorbance at λ_{max} versus the corresponding concentration to determine the molecular decadic attenuation coefficient ε from the slope (optical pass length = 1 cm). Right: UV-Vis-NIR and fluorescence spectrum shown in comparison.

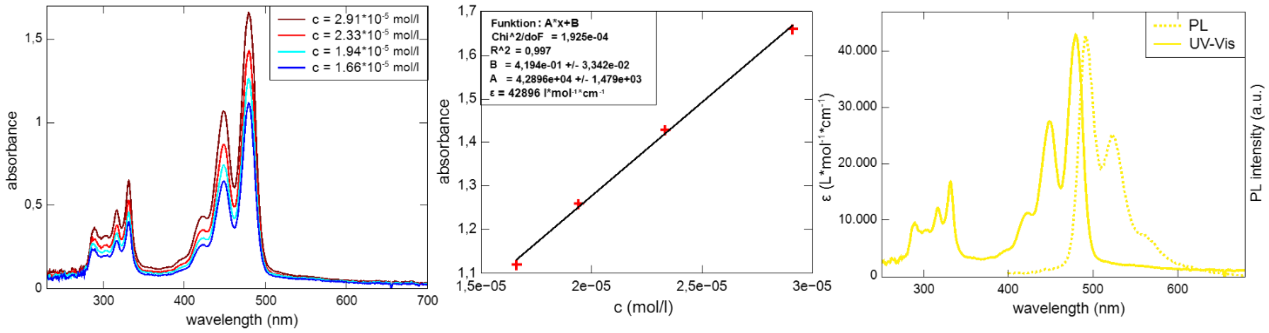

Figure S3. Left: UV-Vis-NIR spectra of peropyrene **4** at four different concentration, recorded in CH_2Cl_2. Center: plot of the absorbance at λ_{max} versus the corresponding concentration to determine the molecular decadic attenuation coefficient ε from the slope (optical pass length = 1 cm). Right: UV-Vis-NIR and fluorescence spectrum shown in comparison.

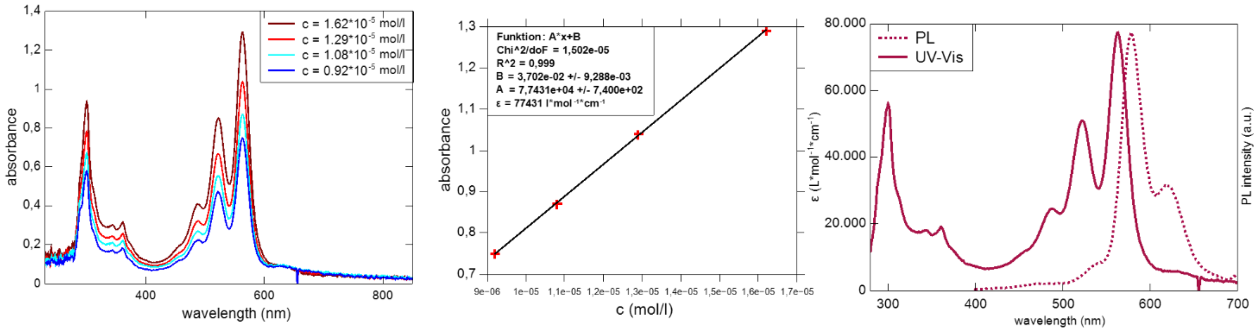

Figure S4. Left: UV-Vis-NIR spectra of terropyrene **13** at four different concentration, recorded in CH_2Cl_2. Center: plot of the absorbance at λ_{max} versus the corresponding concentration to determine the molecular decadic attenuation coefficient ε from the slope (optical pass length = 1 cm). Right: UV-Vis-NIR and fluorescence spectrum shown in comparison.

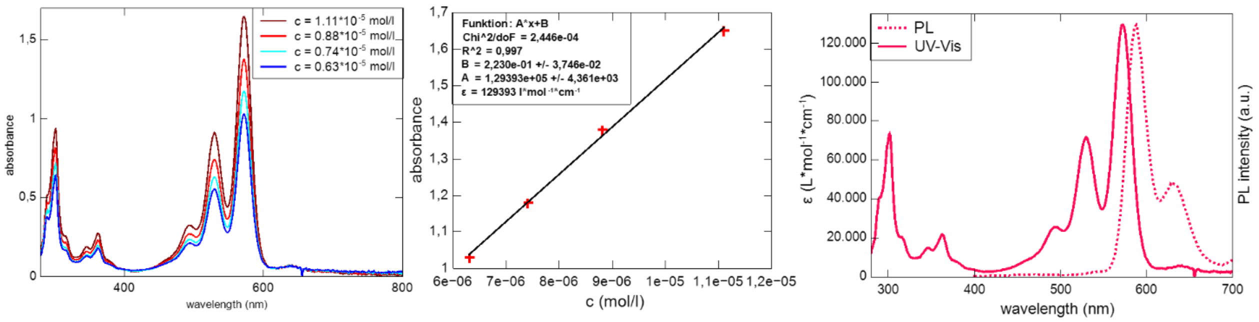

Figure S5. Left: UV-Vis-NIR spectra of terropyrene **14** at four different concentration, recorded in CH_2Cl_2. Center: plot of the absorbance at λ_{max} versus the corresponding concentration to determine the molecular decadic attenuation coefficient ε from the slope (optical pass length = 1 cm). Right: UV-Vis-NIR and fluorescence spectrum shown in comparison.

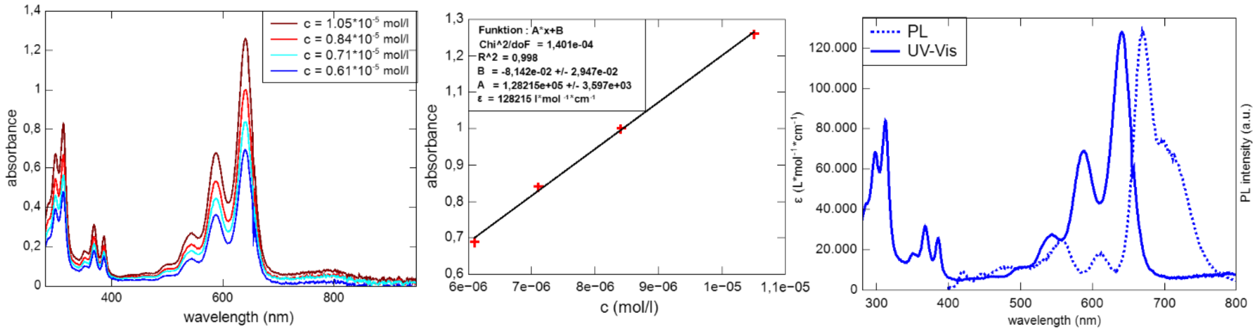

Figure S6. Left: UV-Vis-NIR spectra of quarterropyrene **15** at four different concentration, recorded in CH_2Cl_2. Center: plot of the absorbance at λ_{max} versus the corresponding concentration to determine the molecular decadic attenuation coefficient ε from the slope (optical pass length = 1 cm). Right: UV-Vis-NIR and fluorescence spectrum shown in comparison.

Determination of fluorescence quantum yields

In the following figure S7, the “emission versus absorbance” plots and the linear regression graphs for the determination of the fluorescence quantum yields of **4**, **13** and **14** as well as the standard fluorescein are shown. Note that in the case of triisopropylsilyl-substituted **5** and **15**, the emission values at suitable low concentrations were too low to determine fluorescence quantum yields.

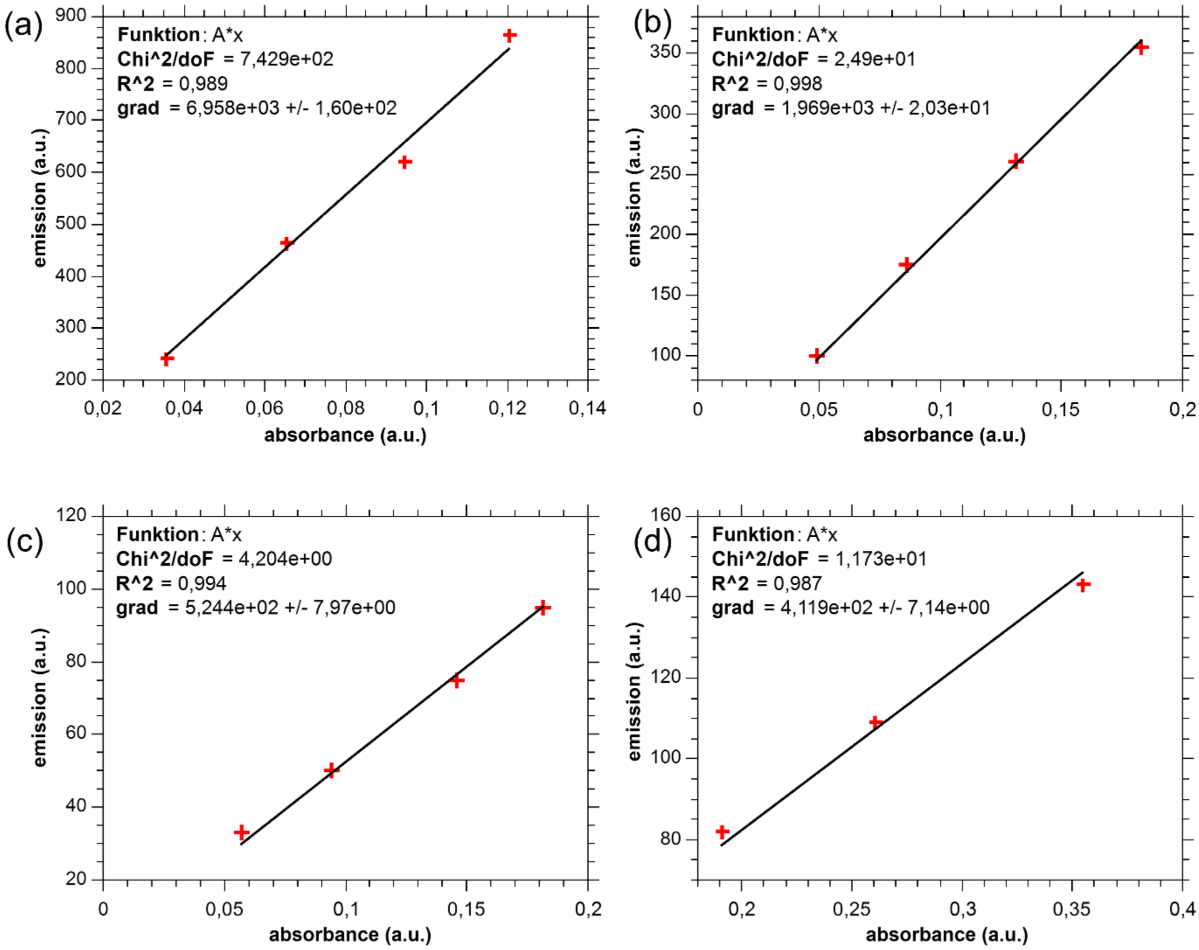

Figure S7. “emission versus absorption” plots of (a) reference fluorescein in 0.1 M NaOH solution; (b) compound **4** in dichloromethane; (c) compound **13** in dichloromethane and (d) compound **14** in dichloromethane. The concentrations were in the low 10^{-6} M range.

Comparison of TMS vs. TIPS substituted terropyrene

In order to verify whether a simplification of a TIPS-group by the smaller TMS-group is justified to simplify DFT calculations, we compared the optical and electrochemical properties of terropyrene trimethylsilyl ether **13** and terropyrene triisopropylsilyl ether **14** (figure S8). It can be clearly noted, that the UV-Vis and photoluminescence show only minor alterations. TIPS ether **14** is about 10 nm bathocromically shifted with respect to TMS ether **13**. This leads to an experimental error of only 0.03 eV for the optical bandgap determination from the intersection wavelength of the normalized UV-Vis and photoluminescence spectra. The cyclovoltammogramms of **13** and **14** show almost identical oxidation halfwave potential voltages. Whereas the two oxidation waves of **14** are almost reversible, the oxidation waves of **13** possess a more irreversible character. This indicates a possible decomposition of the more labile TMS ether groups of **13**. The first oxidation halfwave potential, which is crucial for the experimental determination of the HOMO energy, alters only about 0.02 V, leading to an experimentally small error of 0.02 eV for the HOMO energy due to the simplification.

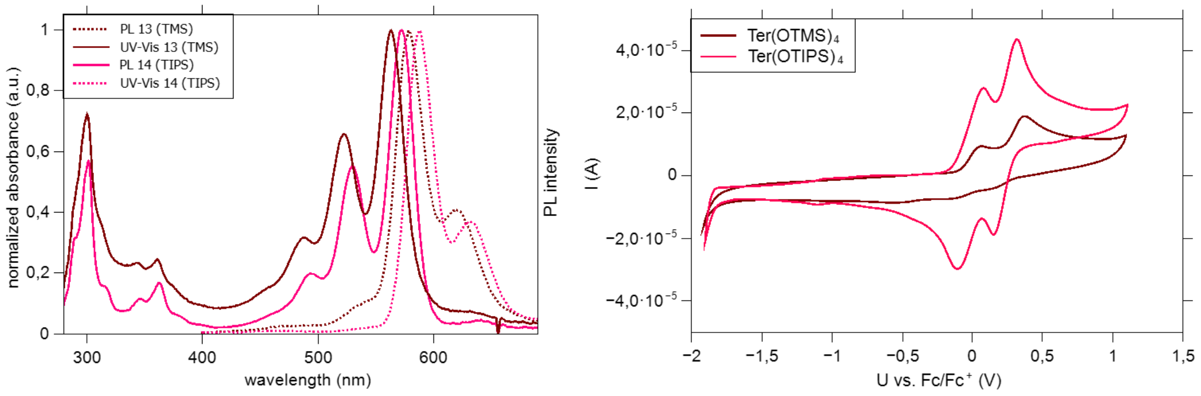

Figure S8. Left: Comparison of normalized UV-Vis-NIR spectra (solid lines) of **13** (red) and **14** (pink) and their corresponding photoluminescence spectra (dashed lines). Right: Comparison of the cyclovoltammogramms of **13** (red) and **14** (pink), referenced with ferrocene (measured in CH_2Cl_2, 0.1 M *n*-Bu_4NPF_6, 100 mV s^{-1} scan rate, glassy carbon working electrode, platinum reference electrode).

Cyclovoltammetry raw data and spectra referenced on ferrocene

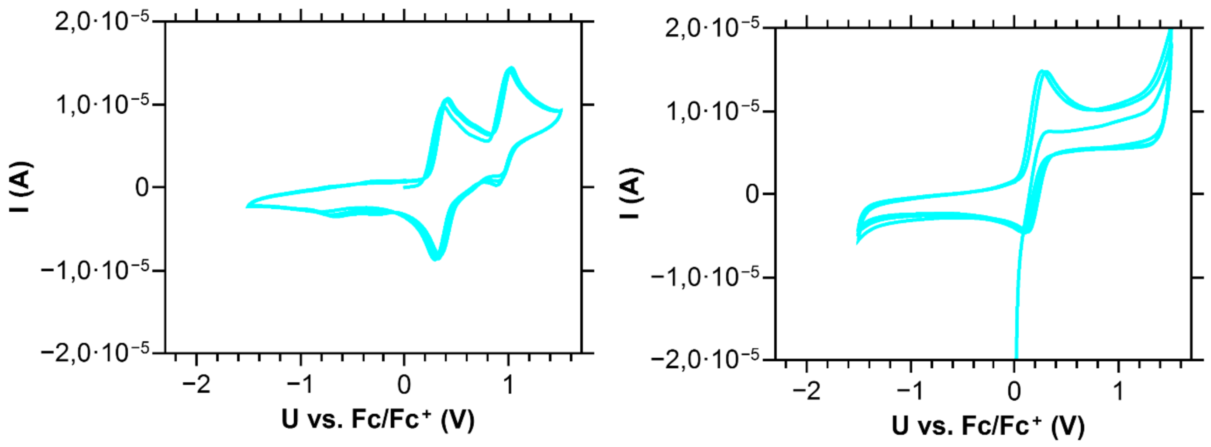

Figure S9. Left: Raw data of the cyclovoltammography of pyrene **5** (three scans were performed, the third one was used for analysis). Right: Cyclovoltammogramm of **5** after addition of 1 mM ferrocene (measured in CH_2Cl_2, 0.1 M *n*-Bu_4NPF_6, 100 mV s^{-1} scan rate, glassy carbon working electrode, platinum reference electrode).

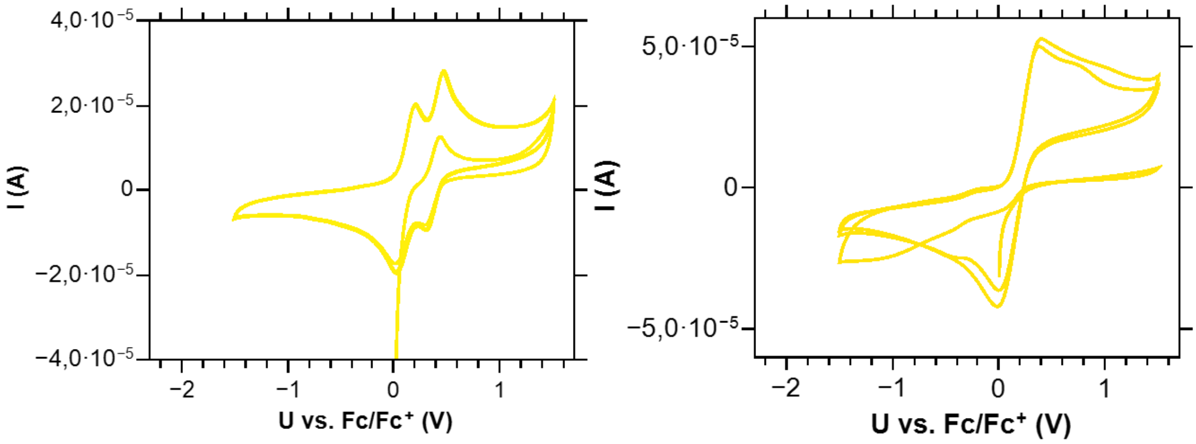

Figure S10. Left: Raw data of the cyclovoltammography of peropyrene **4** (three scans were performed, the third one was used for analysis). Right: Cyclovoltammogramm of **4** after addition of 1 mM ferrocene (measured in CH_2Cl_2, 0.1 M *n*-Bu_4NPF_6, 100 mV s^{-1} scan rate, glassy carbon working electrode, platinum reference electrode).

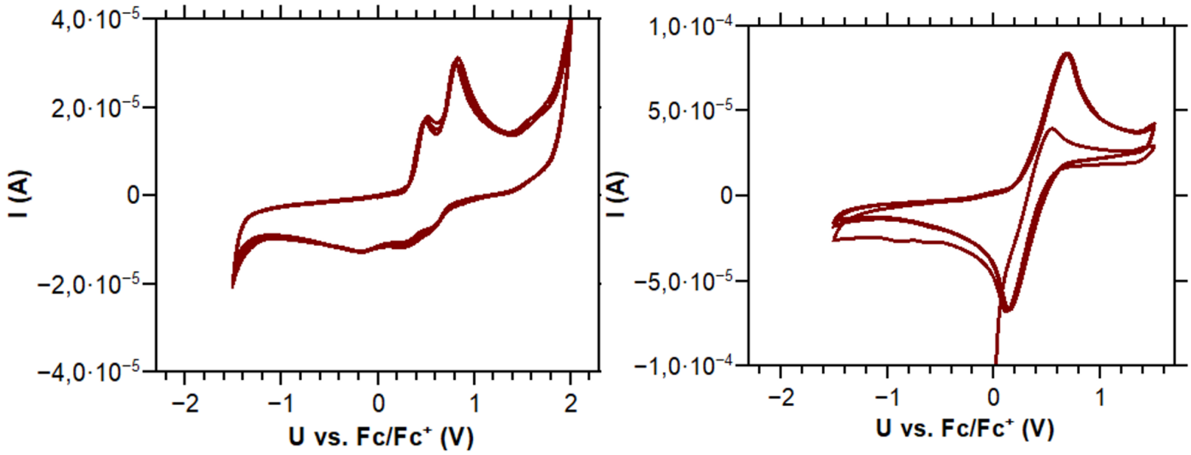

Figure S11. Left: Raw data of the cyclovoltammography of terropyrene **13** (three scans were performed, the third one was used for analysis). Right: Cyclovoltammogramm of **13** after addition of 1 mM ferrocene (measured in CH_2Cl_2, 0.1 M *n*-Bu_4NPF_6, 100 mV s^{-1} scan rate, glassy carbon working electrode, platinum reference electrode).

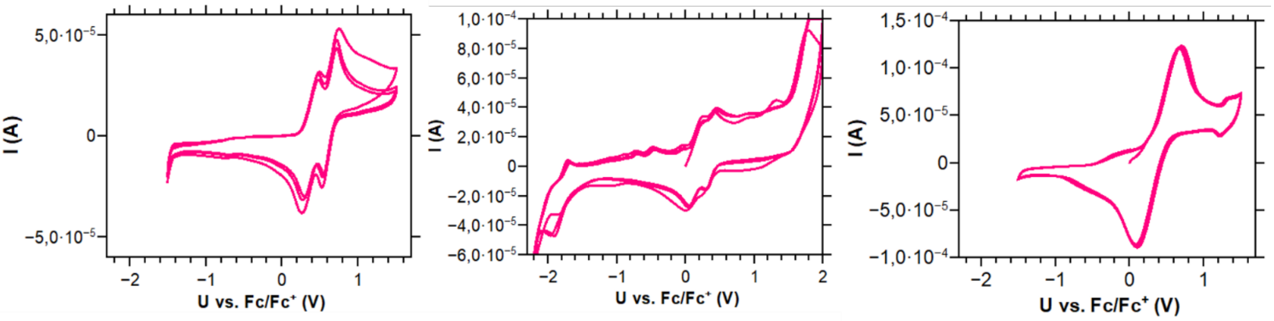

Figure S12. Left: Raw data of the cyclovoltammography of terropyrene **14** (three scans were performed, the third one was used for analysis). Center: Cyclovoltammogramm of **14** at larger voltage window to assign reduction potentials (note: this leads to decomposition of **14**). Right: Cyclovoltammogramm of **14** after addition of 1 mM ferrocene (measured in CH_2Cl_2, 0.1 M *n*-Bu_4NPF_6, 100 mV s^{-1} scan rate, glassy carbon working electrode, platinum reference electrode).

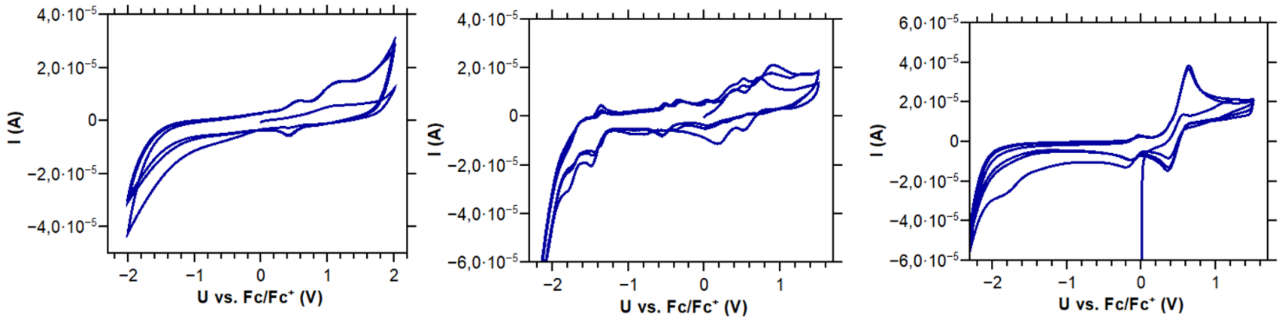

Figure S13. Left: Raw data of the cyclovoltammography of terropyrene **15** (three scans were performed, the third one was used for analysis). Center: Cyclovoltammogramm of **15** at larger voltage window to assign reduction potentials (note: this leads to decomposition of **15**). Right: Cyclovoltammogramm of **15** after addition of 1 mM ferrocene (measured in CH_2Cl_2, 0.1 M *n*-Bu_4NPF_6, 100 mV s^{-1} scan rate, glassy carbon working electrode, platinum reference electrode).

Density functional theory (DFT)

Density functional theory (DFT) calculations using the B3LYP[7] functional were performed. The def2-TZVPP[8,9] basis set was used with RIJCOSX auxiliary base set, employing the resolution-of-identity approximation.[9,10] For radical cation **[15-TMS]**$^+$, DFT calculations were performed using the CAM-B3LYP functional[11] with LANL2DZ base set.[12] Further D3-dispersion correction[13] was considered by applying Becke−Johnson damping.[14] Structural optimizations and TD-DFT calculations were performed using Orca 3.0.3.[15] The atomic coordinates for geometry optimization were taken from XRD structures, if possible. The structurally optimized molecules were used for TD-DFT calculations using the PBE functional[16] (PBE0-D3/def2-TZVPP), employing the resolution-of-identity approximation for both Coulomb integrals and HF exchange integrals.[17] For radical cation **[15-TMS]**$^+$, TD-DFT calculations were performed using the CAM-B3LYP functional with LANL2DZ base set.

Optimized geometries (XYZ-files)

Table S1. Cartesian coordinates (XYZ) of DFT optimized geometry of **5-TMS** (def-TZVPP/B3LYP).

C	-3.503569000	-0.056511000	-0.265835000
C	-2.796103000	-1.251619000	-0.355933000
C	-2.844943000	1.159476000	-0.109971000
C	-1.438855000	1.216686000	-0.092562000
C	-0.713071000	-0.010074000	-0.130621000
C	-1.392071000	-1.258853000	-0.234540000
C	0.717011000	0.013337000	-0.065335000
C	1.437840000	-1.215134000	-0.005917000
C	0.719113000	-2.449146000	-0.071848000
C	-0.632175000	-2.469578000	-0.204884000
C	-0.718399000	2.451127000	-0.048398000
C	0.639503000	2.474274000	-0.060533000
C	1.401176000	1.263917000	-0.061697000
C	2.809900000	1.256571000	-0.054724000
C	3.507929000	0.057302000	0.054022000
C	2.839878000	-1.161430000	0.105340000
O	-3.557155000	2.320582000	-0.020496000
Si	-4.742246000	2.785781000	1.069504000
C	-4.820507000	4.644403000	0.958564000
C	-4.301033000	2.237497000	2.802010000
C	-6.419968000	2.084209000	0.616569000
O	3.536889000	-2.328259000	0.221721000
Si	4.701968000	-2.799051000	1.330626000
C	4.735842000	-4.660458000	1.256844000
C	4.263527000	-2.214090000	3.052021000
C	6.399735000	-2.145937000	0.878740000
O	3.468781000	2.447030000	-0.119416000
Si	4.870960000	2.912841000	-0.908368000
C	4.861729000	4.774486000	-0.846201000
C	4.872332000	2.310350000	-2.678728000
C	6.406070000	2.283779000	-0.036951000
O	-3.435651000	-2.441527000	-0.526597000
Si	-4.813088000	-2.894332000	-1.363926000
C	-4.787935000	-4.757038000	-1.358240000
C	-4.781948000	-2.256255000	-3.121275000
C	-6.377530000	-2.301610000	-0.520557000

H	-4.581907000	-0.065266000	-0.320504000
H	1.282791000	-3.369630000	-0.032688000
H	-1.163993000	-3.406771000	-0.275797000
H	-1.284358000	3.370901000	-0.029489000
H	1.174008000	3.412643000	-0.049395000
H	4.586619000	0.069373000	0.100010000
H	-5.067006000	4.977317000	-0.051017000
H	-5.598484000	5.017284000	1.628369000
H	-3.877687000	5.105290000	1.256810000
H	-4.426034000	1.161070000	2.932178000
H	-4.965453000	2.732916000	3.513926000
H	-3.275311000	2.501248000	3.065282000
H	-7.186413000	2.570204000	1.225485000
H	-6.666400000	2.268569000	-0.430794000
H	-6.486878000	1.011457000	0.807477000
H	4.984247000	-5.019771000	0.256877000
H	5.498118000	-5.037883000	1.942013000
H	3.779271000	-5.092147000	1.555130000
H	4.396572000	-1.136772000	3.165241000
H	4.923825000	-2.703629000	3.771965000
H	3.235890000	-2.466139000	3.319380000
H	7.150839000	-2.658283000	1.485216000
H	6.640488000	-2.332748000	-0.169386000
H	6.500576000	-1.076917000	1.075714000
H	4.987129000	5.142606000	0.173343000
H	5.685310000	5.168795000	-1.444891000
H	3.933512000	5.182549000	-1.248676000
H	5.005895000	1.229819000	-2.751496000
H	5.697078000	2.781286000	-3.218485000
H	3.946822000	2.575911000	-3.192132000
H	7.278190000	2.824168000	-0.413937000
H	6.354069000	2.455382000	1.039857000
H	6.580944000	1.220962000	-0.212169000
H	-4.936653000	-5.160706000	-0.355540000
H	-5.593086000	-5.136155000	-1.991018000
H	-3.846950000	-5.144468000	-1.751796000
H	-4.947191000	-1.179380000	-3.183000000
H	-5.578075000	-2.743209000	-3.689322000
H	-3.836705000	-2.488630000	-3.614685000
H	-7.230582000	-2.848687000	-0.930133000
H	-6.352309000	-2.489782000	0.554321000
H	-6.564358000	-1.238814000	-0.683447000

Table S2. Cartesian coordinates (XYZ) of DFT optimized geometry of **4** (def-TZVPP/B3LYP).

Si	2.987330000	4.283566000	9.345428000
C	2.282950000	6.901980000	8.693827000
O	2.142397000	5.552912000	8.650501000
O	4.377062000	9.489703000	10.228760000
Si	5.742981000	8.974306000	11.036853000
C	3.267858000	7.500646000	9.472167000
H	3.914368000	6.886597000	10.075119000
C	2.775917000	11.112388000	8.662943000
H	3.556355000	11.550097000	9.266931000
C	2.606985000	9.700913000	8.692260000
C	3.432361000	8.879409000	9.477431000
C	1.975137000	11.892306000	7.896258000
H	2.139012000	12.957945000	7.905041000
C	1.577917000	9.102815000	7.918835000
C	0.714871000	9.932133000	7.129884000
C	-0.341497000	9.340205000	6.379239000
C	-1.211727000	10.166666000	5.616093000
C	-0.480068000	7.917248000	6.418919000
H	-1.259889000	7.444312000	5.843481000
C	0.352852000	7.127377000	7.144729000
H	0.223399000	6.054901000	7.146498000
C	1.406232000	7.689649000	7.921133000
C	2.429092000	2.781117000	8.396325000
H	1.356215000	2.615581000	8.507388000
H	2.945074000	1.889018000	8.757848000
H	2.650312000	2.887986000	7.332630000
C	4.838837000	4.488722000	9.165534000
H	5.319673000	3.538913000	9.413870000
H	5.253950000	5.238350000	9.836884000
H	5.123216000	4.756001000	8.146521000
C	2.563538000	4.117832000	11.159643000
H	3.156910000	3.314113000	11.601916000

H	1.510764000	3.873787000	11.311650000
H	2.786553000	5.032147000	11.711905000
C	5.250069000	7.916780000	12.502307000
H	6.125139000	7.664099000	13.105501000
H	4.783128000	6.979158000	12.196249000
H	4.541200000	8.444752000	13.142288000
C	6.854651000	8.012734000	9.876242000
H	6.295246000	7.259329000	9.321344000
H	7.643446000	7.499938000	10.431042000
H	7.328678000	8.672489000	9.147889000
C	6.540833000	10.569714000	11.581414000
H	7.439802000	10.393832000	12.174723000
H	5.849460000	11.158561000	12.186398000
H	6.822660000	11.166488000	10.712126000
Si	-3.374275000	17.232976000	3.913942000
C	-2.512317000	14.610846000	3.982705000
O	-2.294607000	15.952673000	3.935672000
O	-4.768232000	12.075939000	2.599126000
Si	-6.092011000	12.698988000	1.786119000
C	-3.556939000	14.032304000	3.272280000
H	-4.195345000	14.659743000	2.675058000
C	-3.136620000	10.418881000	4.140770000
H	-3.961641000	9.989454000	3.592130000
C	-2.933434000	11.825146000	4.068308000
C	-3.767597000	12.659528000	3.301960000
C	-2.316615000	9.632657000	4.883252000
H	-2.517963000	8.573787000	4.922345000
C	-1.856425000	12.408045000	4.788387000
C	-0.990539000	11.574969000	5.570174000
C	0.085332000	12.162193000	6.296424000
C	0.921408000	11.343387000	7.101676000
C	0.285143000	13.573412000	6.188417000
H	1.115925000	14.033025000	6.698525000
C	-0.532727000	14.364188000	5.449533000
H	-0.351272000	15.426479000	5.377832000
C	-1.637146000	13.813582000	4.741000000
C	-2.306110000	18.758465000	3.973122000
H	-1.581720000	18.766663000	3.157422000
H	-2.926723000	19.652923000	3.880543000
H	-1.754169000	18.822363000	4.912158000
C	-4.465092000	17.122582000	5.426591000
H	-5.264589000	17.865432000	5.389555000
H	-4.930367000	16.139500000	5.520416000
H	-3.876968000	17.306830000	6.327970000
C	-4.381368000	17.257330000	2.329435000
H	-4.837556000	18.241314000	2.195875000
H	-3.737352000	17.069096000	1.467884000
H	-5.189582000	16.525618000	2.318192000
C	-5.552038000	13.652756000	0.266403000
H	-6.429056000	13.965841000	-0.305700000
H	-4.982851000	14.550766000	0.507648000
H	-4.932512000	13.031201000	-0.382172000
C	-7.092759000	13.794312000	2.933710000
H	-6.477267000	14.557257000	3.411314000
H	-7.885359000	14.305075000	2.382238000
H	-7.559486000	13.204367000	3.724385000
C	-7.051209000	11.176254000	1.295515000
H	-7.932550000	11.434758000	0.705506000
H	-6.430229000	10.509697000	0.694725000
H	-7.385238000	10.625882000	2.176654000

Table S3. Cartesian coordinates (XYZ) of DFT optimized geometry of **13** (def-TZVPP/B3LYP).

Si	7.093583000	14.226517000	6.979288000
O	6.300563000	12.917195000	7.658350000
C	8.632505000	13.685908000	6.054776000
C	7.584546000	15.262202000	8.447965000
C	5.909491000	15.131242000	5.847994000
C	5.502166000	11.979614000	7.090919000
H	8.417823000	13.228408000	5.088332000
H	9.268381000	14.554111000	5.866408000
H	9.211527000	12.972508000	6.643570000
H	8.213144000	14.687654000	9.130345000
H	6.706875000	15.602346000	8.999867000
H	8.148645000	16.142276000	8.132964000

H	5.104714000	15.592360000	6.423211000
H	5.451833000	14.458198000	5.121687000
H	6.423555000	15.919326000	5.293506000
C	5.722708000	11.540930000	5.792785000
C	4.450202000	11.448284000	7.856043000
C	4.917397000	10.563986000	5.220372000
H	6.540269000	11.957513000	5.229883000
C	4.189891000	11.887860000	9.183122000
C	3.623426000	10.444658000	7.281709000
O	5.137905000	10.124557000	3.956923000
C	3.852501000	9.998575000	5.950061000
C	3.167367000	11.361171000	9.902832000
H	4.824596000	12.651260000	9.607251000
C	2.552508000	9.882795000	8.050085000
Si	6.262720000	10.596370000	2.803829000
C	3.000057000	8.997555000	5.406394000
C	2.316720000	10.340747000	9.378958000
H	3.006957000	11.725779000	10.904832000
C	1.730749000	8.865135000	7.486273000
C	8.020904000	10.384810000	3.415601000
C	5.972585000	12.377792000	2.299944000
C	5.947826000	9.418098000	1.397880000
C	1.991273000	8.459609000	6.140518000
H	3.170003000	8.671177000	4.390732000
C	1.255167000	9.780173000	10.142355000
C	0.687749000	8.289632000	8.265848000
H	8.219685000	9.349831000	3.699904000
H	8.707823000	10.652759000	2.608334000
H	8.265978000	11.011611000	4.270970000
H	4.925435000	12.558306000	2.049691000
H	6.257259000	13.079256000	3.085443000
H	6.569233000	12.616600000	1.416528000
H	6.605266000	9.633144000	0.552939000
H	6.134589000	8.391296000	1.717754000
H	4.916747000	9.483259000	1.045644000
H	1.367762000	7.709447000	5.680753000
C	0.444962000	8.747004000	9.593006000
C	0.967408000	10.217273000	11.466519000
C	-0.136782000	7.242840000	7.765659000
C	-0.617034000	8.169697000	10.374921000
C	-0.034007000	9.672441000	12.202959000
H	1.553084000	11.007133000	11.908778000
C	-1.138480000	6.698320000	8.501856000
H	0.032825000	6.857266000	6.772613000
C	-0.860093000	8.627262000	11.701979000
C	-1.427709000	7.137164000	9.825139000
H	-0.202575000	10.056972000	13.196592000
H	-1.723249000	5.907509000	8.060092000
C	-1.905775000	8.054369000	12.480026000
C	-2.491942000	6.579532000	10.586711000
C	-2.167958000	8.460862000	13.825208000
C	-2.729502000	7.039120000	11.914703000
C	-3.344189000	5.561139000	10.061524000
C	-3.180764000	7.927008000	14.556881000
H	-1.542870000	9.208897000	14.286329000
C	-3.804583000	6.481624000	12.680538000
C	-4.370630000	5.038727000	10.778675000
H	-3.182211000	5.195132000	9.060287000
C	-4.035918000	6.929336000	14.011255000
H	-3.352131000	8.254430000	15.571969000
C	-4.633484000	5.480768000	12.104425000
H	-5.006836000	4.277205000	10.353410000
C	-5.105850000	6.369170000	14.737717000
C	-5.690421000	4.954868000	12.866227000
O	-5.329249000	6.810731000	15.999935000
C	-5.913708000	5.395666000	14.163133000
O	-6.491468000	4.020784000	12.296602000

Si	-6.455291000	6.338648000	17.151789000
H	-6.735783000	4.983799000	14.722933000
Si	-7.283303000	2.709453000	12.973141000
C	-8.213327000	6.549823000	16.539133000
C	-6.165409000	4.557315000	17.656001000
C	-6.142184000	7.517137000	18.557934000
C	-8.824385000	3.246474000	13.896161000
C	-7.770173000	1.674656000	11.502411000
C	-6.099848000	1.806077000	14.106075000
H	-8.411684000	7.584322000	16.252785000
H	-8.900320000	6.283792000	17.346984000
H	-8.458906000	5.921393000	15.685110000
H	-5.118648000	4.376959000	17.907954000
H	-6.448802000	3.855740000	16.870140000
H	-6.763452000	4.318593000	18.538505000
H	-6.800368000	7.301786000	19.402220000
H	-6.329132000	8.543853000	18.237897000
H	-5.111423000	7.452441000	18.911177000
H	-8.611274000	3.704743000	14.862600000
H	-9.458250000	2.376790000	14.084499000
H	-9.404768000	3.958313000	13.306822000
H	-8.398080000	2.249319000	10.819476000
H	-6.890888000	1.336451000	10.951874000
H	-8.333788000	0.793407000	11.814928000
H	-5.293033000	1.346399000	13.532589000
H	-5.644977000	2.479929000	14.833418000
H	-6.613788000	1.017115000	14.659429000

Table S4. Cartesian coordinates (XYZ) of DFT optimized geometry of **15-TMS** (def-TZVPP/B3LYP).

C	-0.682933000	9.064764000	-0.003636000
C	-0.769781000	7.660440000	0.033817000
C	0.425821000	6.895499000	-0.060703000
C	1.680661000	7.545362000	-0.214167000
C	1.714258000	8.953009000	-0.308297000
C	0.543013000	9.695761000	-0.187461000
C	-2.013477000	6.980959000	0.154947000
C	-2.071607000	5.624249000	0.170759000
C	-0.899301000	4.812598000	0.090105000
C	0.363541000	5.463671000	-0.008840000
C	1.562326000	4.695218000	-0.062731000
C	2.800706000	5.395434000	-0.186500000
C	2.859989000	6.750952000	-0.270206000
C	-0.962794000	3.390196000	0.095846000
C	0.232421000	2.617252000	0.055046000
C	1.494782000	3.272216000	-0.003263000
C	-2.201494000	2.692085000	0.134567000
C	-2.265792000	1.335489000	0.129089000
C	-1.098584000	0.523837000	0.094295000
C	0.163520000	1.178965000	0.066093000
C	1.355462000	0.401252000	0.042698000
C	2.595470000	1.097952000	0.021481000
C	2.660728000	2.455828000	-0.004795000
C	-1.170681000	-0.899162000	0.077861000
C	0.019770000	-1.677295000	0.039256000
C	1.283539000	-1.023528000	0.032625000
C	-2.413651000	-1.589848000	0.087666000
C	-2.484575000	-2.945819000	0.061284000
C	-1.321714000	-3.763249000	0.011778000
C	-0.055384000	-3.114140000	-0.001920000
C	1.136059000	-3.890701000	-0.067428000
C	2.378333000	-3.197175000	-0.042590000
C	2.448125000	-1.840726000	0.007499000
C	-1.402028000	-5.183748000	-0.035169000
C	-0.209162000	-5.953698000	-0.143163000
C	1.061232000	-5.309180000	-0.164396000
C	-2.649363000	-5.877248000	0.012575000

C	-2.724835000	-7.232070000	-0.046939000
C	-1.554331000	-8.026830000	-0.188324000
C	-0.289214000	-7.380313000	-0.242363000
C	0.897152000	-8.145133000	-0.409667000
C	2.150253000	-7.472912000	-0.436025000
C	2.225583000	-6.123593000	-0.305353000
C	-1.608844000	-9.430757000	-0.287519000
C	-0.447866000	-10.170709000	-0.471787000
C	0.787113000	-9.539936000	-0.549007000
O	-1.827945000	9.793310000	0.089324000
Si	-2.357838000	10.901357000	1.229346000
C	-4.214676000	10.907345000	1.079324000
C	-1.699285000	12.613226000	0.858332000
C	-1.826327000	10.381980000	2.944272000
O	2.919956000	9.548176000	-0.492353000
Si	3.414243000	10.936850000	-1.290456000
C	5.274046000	10.869089000	-1.241176000
C	2.829848000	12.485319000	-0.415468000
C	2.803257000	10.941277000	-3.056860000
O	-2.812734000	-10.050529000	-0.167381000
Si	-3.639803000	-11.040554000	-1.237986000
C	-2.701663000	-12.635724000	-1.543520000
C	-3.880754000	-10.102250000	-2.837036000
C	-5.255868000	-11.388198000	-0.370511000
O	1.899425000	-10.274733000	-0.815099000
Si	2.776815000	-11.279599000	0.192639000
C	3.606643000	-10.254757000	1.511916000
C	4.020610000	-12.082967000	-0.934533000
C	1.650210000	-12.546642000	0.977325000
H	-2.917588000	7.568132000	0.217178000
H	-3.038435000	5.154358000	0.254138000
H	3.723029000	4.838007000	-0.228665000
H	3.811692000	7.250176000	-0.374891000
H	-3.126174000	3.246039000	0.157048000
H	-3.239264000	0.872549000	0.150212000
H	3.520219000	0.542807000	0.021528000
H	3.635351000	2.917680000	-0.021048000
H	-3.335818000	-1.032005000	0.113216000
H	-3.460255000	-3.404895000	0.064706000
H	3.300944000	-3.755599000	-0.060456000
H	3.424229000	-1.382389000	0.026738000
H	-3.565100000	-5.316782000	0.110251000
H	-3.681195000	-7.730390000	0.008370000
H	3.047921000	-8.057495000	-0.567835000
H	3.199067000	-5.660249000	-0.328567000
H	-4.640680000	9.941943000	1.357723000
H	-4.637638000	11.660889000	1.746832000
H	-4.533868000	11.147399000	0.063783000
H	-0.626575000	12.691149000	1.047480000
H	-2.197741000	13.342021000	1.501823000
H	-1.893291000	12.899266000	-0.177080000
H	-2.338965000	11.002573000	3.682907000
H	-2.082400000	9.341161000	3.150631000
H	-0.753183000	10.510954000	3.095951000
H	5.649700000	9.924269000	-1.636919000
H	5.689620000	11.673191000	-1.851574000
H	5.653432000	10.993252000	-0.225675000
H	3.006323000	12.425510000	0.660140000
H	1.770786000	12.688182000	-0.583459000
H	3.390431000	13.343960000	-0.793301000
H	3.289859000	11.752563000	-3.602918000
H	3.051042000	10.007701000	-3.564863000
H	1.725582000	11.096535000	-3.129723000
H	-2.408309000	-13.104374000	-0.601824000
H	-3.333178000	-13.344304000	-2.084272000
H	-1.801794000	-12.478765000	-2.139730000
H	-4.521133000	-9.231267000	-2.691091000

H	-4.339953000	-10.735030000	-3.599137000
H	-2.923827000	-9.747899000	-3.226748000
H	-5.927997000	-11.956916000	-1.016058000
H	-5.100037000	-11.967695000	0.540873000
H	-5.756754000	-10.458950000	-0.094089000
H	2.874558000	-9.692818000	2.095357000
H	4.163907000	-10.893669000	2.201432000
H	4.306243000	-9.540314000	1.074963000
H	3.522165000	-12.686743000	-1.694613000
H	4.687025000	-12.736110000	-0.366360000
H	4.633208000	-11.336377000	-1.442554000
H	2.231658000	-13.226233000	1.605347000
H	0.897144000	-12.073129000	1.609302000
H	1.134717000	-13.146302000	0.224792000
H	-0.499445000	-11.244327000	-0.563003000
H	0.575827000	10.773442000	-0.240764000

Table S5. Cartesian coordinates (XYZ) of DFT optimized geometry of **[15-TMS]$^+$** (LANL2DZ/CAM-B3LYP).

C	-0.817269000	9.307968000	0.237929000
C	-0.886000000	7.886600000	0.230987000
C	0.320866000	7.134440000	0.196101000
C	1.576874000	7.801961000	0.179732000
C	1.606921000	9.224989000	0.201052000
C	0.418753000	9.963054000	0.228405000
C	-2.131632000	7.201256000	0.241646000
C	-2.182480000	5.828616000	0.204362000
C	-0.996232000	5.036190000	0.161403000
C	0.269688000	5.701005000	0.166371000
C	1.484925000	4.948052000	0.130951000
C	2.724268000	5.657158000	0.124620000
C	2.771040000	7.030816000	0.144184000
C	-1.045569000	3.596182000	0.106736000
C	0.163744000	2.837762000	0.069093000
C	1.425158000	3.507604000	0.088351000
C	-2.274964000	2.891719000	0.080833000
C	-2.326087000	1.515353000	0.012554000
C	-1.149876000	0.728298000	-0.025348000
C	0.110190000	1.400855000	0.008889000
C	1.317611000	0.638653000	-0.028158000
C	2.548385000	1.338536000	0.000713000
C	2.599730000	2.715506000	0.057539000
C	-1.205289000	-0.716049000	-0.104898000
C	0.002220000	-1.478249000	-0.143652000
C	1.261963000	-0.805723000	-0.101274000
C	-2.434819000	-1.415901000	-0.147036000
C	-2.485523000	-2.792786000	-0.230504000
C	-1.311746000	-3.583796000	-0.268801000
C	-0.050581000	-2.914326000	-0.221151000
C	1.159338000	-3.672290000	-0.257399000
C	2.387265000	-2.968794000	-0.212725000
C	2.437662000	-1.592092000	-0.134775000
C	-1.370298000	-5.025329000	-0.356961000
C	-0.154119000	-5.778044000	-0.393565000
C	1.111397000	-5.113560000	-0.341070000
C	-2.607155000	-5.732461000	-0.403849000
C	-2.653191000	-7.106420000	-0.486167000
C	-1.460239000	-7.874718000	-0.523740000
C	-0.203820000	-7.210091000	-0.475273000
C	1.004463000	-7.960635000	-0.503805000
C	2.247333000	-7.277706000	-0.448094000
C	2.296393000	-5.904329000	-0.368199000
C	-1.493285000	-9.299396000	-0.606088000
C	-0.300567000	-10.036247000	-0.629597000
C	0.938988000	-9.384249000	-0.577748000
O	-1.979024000	10.007082000	0.280622000
Si	-2.784744000	11.172440000	-0.728223000

C	-1.619279000	12.605605000	-1.075024000
C	-4.258249000	11.654264000	0.314643000
C	-3.244496000	10.277757000	-2.318133000
O	2.809139000	9.847607000	0.147667000
Si	3.785471000	10.876228000	1.156343000
C	5.296972000	11.193528000	0.099541000
C	4.175642000	9.884574000	2.707908000
C	2.836841000	12.452227000	1.558975000
O	-2.707872000	-9.886199000	-0.656141000
Si	-3.484805000	-11.419828000	-0.942289000
C	-3.814155000	-12.192763000	0.741294000
C	-2.345142000	-12.491540000	-1.991368000
C	-5.052710000	-10.943368000	-1.858931000
O	2.111136000	-10.057050000	-0.604353000
Si	2.761189000	-11.652720000	-0.330616000
C	1.581751000	-12.633649000	0.758950000
C	4.382407000	-11.293594000	0.545767000
C	2.943375000	-12.462920000	-2.016754000
H	-3.039646000	7.790406000	0.286008000
H	-3.152671000	5.351507000	0.212285000
H	3.658894000	5.113936000	0.093736000
H	3.719501000	7.554023000	0.120658000
H	-3.211489000	3.432041000	0.109780000
H	-3.299597000	1.045177000	-0.011131000
H	3.484960000	0.798532000	-0.024869000
H	3.573471000	3.186202000	0.075804000
H	-3.371552000	-0.876177000	-0.117110000
H	-3.459593000	-3.262014000	-0.265123000
H	3.324589000	-3.507699000	-0.240244000
H	3.410746000	-1.121656000	-0.101637000
H	-3.542567000	-5.190303000	-0.374178000
H	-3.600123000	-7.631831000	-0.520991000
H	3.155298000	-7.868294000	-0.470578000
H	3.266318000	-5.428431000	-0.325624000
H	-0.785893000	12.302832000	-1.719153000
H	-2.159051000	13.406527000	-1.595631000
H	-1.203157000	13.034263000	-0.155928000
H	-4.767994000	10.765879000	0.702674000
H	-4.983285000	12.238017000	-0.263560000
H	-3.947270000	12.258216000	1.173352000
H	-3.713114000	10.966559000	-3.031368000
H	-2.363618000	9.841531000	-2.803883000
H	-3.955829000	9.467209000	-2.128407000
H	5.058303000	11.833614000	-0.755598000
H	6.082415000	11.690530000	0.681415000
H	5.707694000	10.257254000	-0.293581000
H	4.826524000	9.033486000	2.480540000
H	3.267819000	9.492326000	3.181371000
H	4.694170000	10.507389000	3.446795000
H	3.489449000	13.152067000	2.095515000
H	2.485256000	12.957456000	0.652489000
H	1.970992000	12.260772000	2.202421000
H	-4.292364000	-11.471099000	1.412238000
H	-4.488143000	-13.052773000	0.654299000
H	-2.893524000	-12.547015000	1.218516000
H	-1.839528000	-11.905550000	-2.767274000
H	-2.926371000	-13.271797000	-2.497186000
H	-1.581760000	-12.994742000	-1.387074000
H	-5.586589000	-11.827262000	-2.228375000
H	-5.734823000	-10.384548000	-1.209544000
H	-4.823046000	-10.311492000	-2.722817000
H	0.769429000	-13.088772000	0.180267000
H	2.123654000	-13.447379000	1.255569000
H	1.135810000	-12.012298000	1.543480000
H	5.076475000	-10.756775000	-0.108766000
H	4.874315000	-12.214463000	0.881066000
H	4.209378000	-10.672348000	1.430521000

H	3.538467000	-13.381097000	-1.955125000
H	1.969260000	-12.733731000	-2.440611000
H	3.444697000	-11.793028000	-2.722753000
H	-0.336681000	-11.116153000	-0.691224000
H	0.459187000	11.045781000	0.238730000

TD-DFT calculations

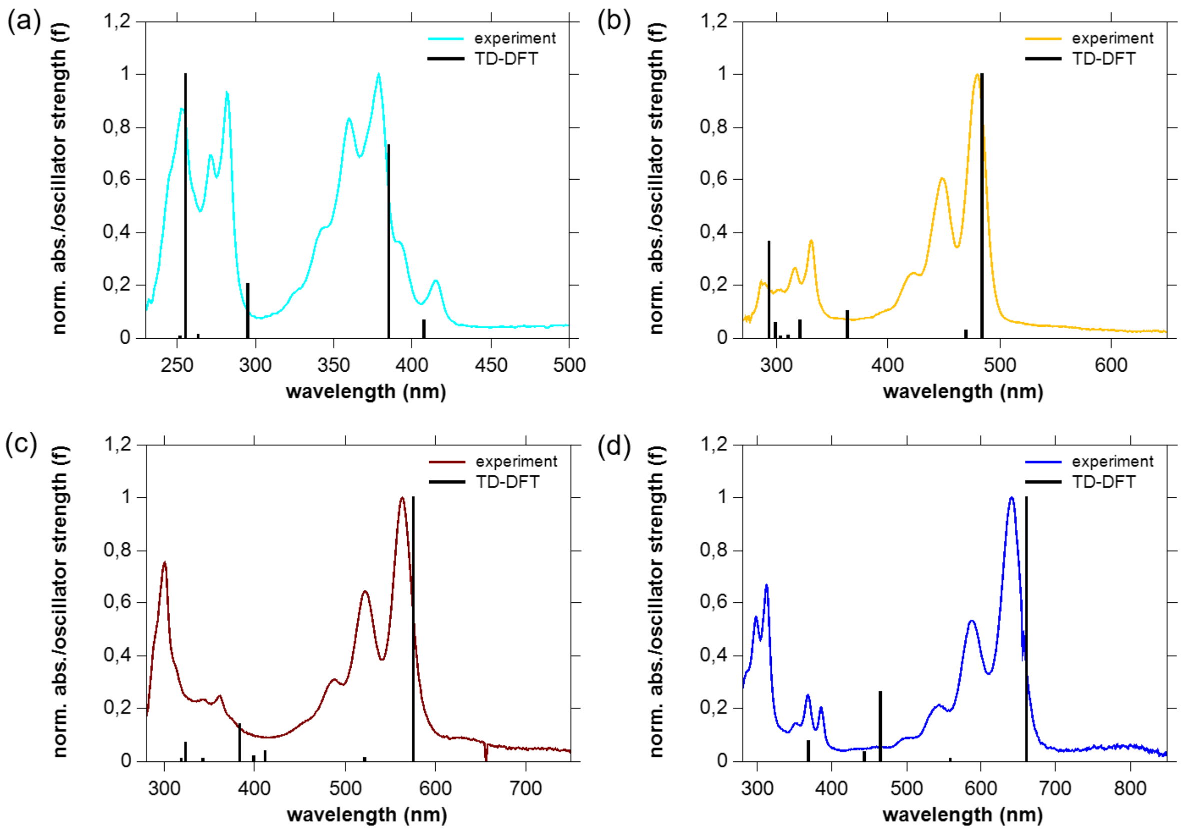

Figure S14. Comparison of normalized UV-Vis-NIR spectra (recorded in CH_2Cl_2) and normalized electron transitions calculated by TD-DFT (black bars, def-TZVPP/PBE level of theory) of a) pyrene **5-TMS**, b) peropyrene **4**, c) terropyrene **13** and d) quarterropyrene **15**.

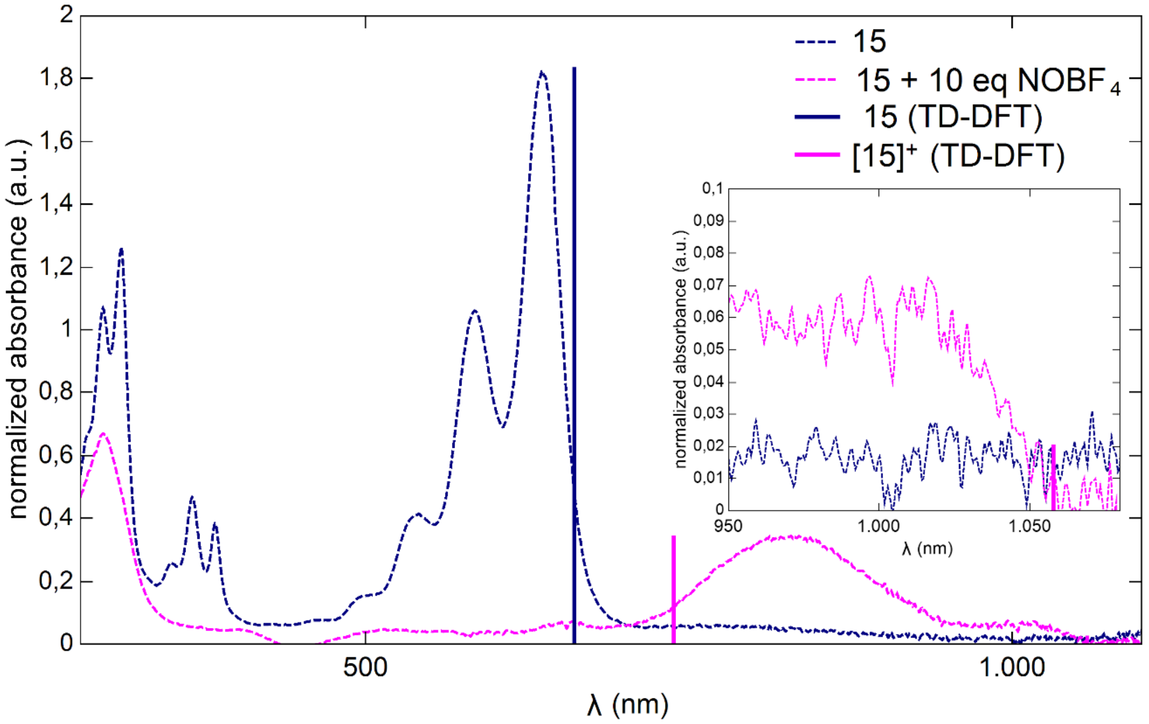

Figure S15. Comparison of normalized UV-Vis-NIR spectra of **15** (blue) and **15** after addition of 10 eq $NOBF_4$ (magenta), recorded in CH_2Cl_2/acetonitrile and main electron transitions calculated by TD-DFT (bars, normalized on absorption maxima) for **15-TMS** and radical cation **[15]**$^+$ (LANL2DZ/CAM-B3LYP level of theory).

Table S6. Characteristic main electron transition of **4**, **5**, **13** and **15**, calculated using TD-DFT (def2-TZVPP/PBE level of theory) and **[15]**$^+$ (LANL2DZ/CAM-B3LYP level of theory).

Compound	Transition energy (wavelength)	Oscillator strength	Description of main contributions
**5-TMS**	3.05 eV (407 nm)	0.054	HOMO→LUMO+1 (c = 0.915); 0.837<br>HOMO-1→LUMO (c = -0.368); 0.136<br>HOMO-2→LUMO+1 (c = 0.109); 0.012
	3.22 eV (385 nm)	0.626	HOMO→LUMO+1 (c = -0.916); 0.839<br>HOMO-1→LUMO+1 (c = -0.297); 0.088<br>HOMO-3→LUMO+2 (c = 0.160); 0.026<br>HOMO-2→LUMO+5 (c = 0.102); 0.011
**4**	2.56 eV (484 nm)	1.074	HOMO→LUMO (c = -0.929); 0.862<br>HOMO-2→LUMO+1 (c = -0.267); 0.071<br>HOMO-1→LUMO+3 (c = 0.115); 0.013<br>HOMO-4→LUMO+2 (c = -0.109); 0.012
**13**	2.15 eV (575 nm)	1.789	HOMO→LUMO (c = -0.937); 0.878<br>HOMO-3→LUMO (c = -0.140); 0.020<br>HOMO-2→LUMO (c = 0.219); 0.048<br>HOMO-1→LUMO+2 (c = -0.145); 0.021
**15-TMS**	1.85 eV (671 nm)	0.002	HOMO-1→LUMO (c = 0.735); 0.540<br>HOMO→LUMO+1 (c = -0.677); 0.459
	1.88 eV (661 nm)	2.458	HOMO→LUMO (c = -0.936); 0.875<br>HOMO-1→LUMO+1 (c = 0.179); 0.032<br>HOMO-3→LUMO (c = 0.179); 0.032<br>HOMO-2→LUMO+2 (c = -0.173); 0.030
**[15]**$^+$	1.17 eV (1057 nm)	0.110	SOMOβ→LUMOβ (c = -0.833), 0.693<br>SOMO-1β→LUMO+1β (c = -0.144), 0.021<br>SOMOα→LUMOα (c = -0.509), 0.259
	1.68 eV (739 nm)	2.729	SOMOβ→LUMOβ (c = -0.474), 0.225<br>SOMO-1β→LUMO+1β (c = -0.139), 0.019<br>SOMOα→LUMOα (c = 0.797), 0.635<br>SOMO-1α→LUMO+1α (c = 0.198), 0.039<br>SOMO-3α→LUMO+2α (c = 0.107), 0.012

Single crystal X-ray structures

Crystal Data

	**1,3,8,10-tetrakis((trimethylsilyl)oxy)dibenzo-[*cd*,*lm*]-perylene (4)**	**1,3,10,12-tetrakis((trimethylsilyl)oxy) benzo[rst]dinaphtho[8,1,2-*cde*:2',1',8'-*klm*]pentaphene (13)**	**1,3,10,12-tetrakis((triisopropylsilyl)oxy)benzo[*rst*]dinaphtho[8,1,2-*cde*:2',1',8'-*klm*]pentaphene (15)**
CCDC code	2055267	2055268	2055269
Identification code	Swp251loesen	swp363loesen	swp388loesen
Empirical formula	C_{38} H_{46} O_4 Si_4	C_{48} H_{50} O_4 Si_4	C_{82} H_{102} O_4 Si_4
Formula weight	679.11	803.24	1263.99
Temperature	100(2) K	100(2) K	100(2) K
Wavelength	1.54178 Å	1.54178 Å	1.54178 Å
Crystal system	Monoclinic	Monoclinic	Monoclinic
Space group	*P*2_1/*c*	*P*2_1/*c*	*P*2_1/*n*
Unit cell dimensions	a = 6.6117(3) Å	a = 6.06940(10) Å	a = 7.6755(4) Å
	b = 21.5680(11) Å	b = 16.9297(4) Å	b = 25.7790(8) Å
	c = 12.9662(6) Å	c = 19.9595(4) Å	c = 17.9782(8) Å
	α = 90°.	α = 90°.	α = 90°.
	β = 91.514(3)°.	β = 90.5320(10)°	β = 100.070(4)°.
	γ = 90°.	γ = 90°	γ = 90°.
Volume	1841.35(15) $Å^3$	2050.81(7) $Å^3$	3502.5(3) $Å^3$
Z	2	2	2
Density (calculated)	1.220 Mg/m^3	1.301 Mg/m^3	1.199 Mg/m^3
Absorption coefficient	1.790 mm^{-1}	1.701 mm^{-1}	1.173 mm^{-1}
F(000)	724	852	1364
Crystal size	0.313 x 0.146 x 0.058 mm^3	0.566 x 0.278 x 0.131 mm^3	0.203 x 0.112 x 0.057 mm^3
Theta range for data collection	3.979 to 69.993°	3.423 to 66.493°.	3.028 to 66.499°.
Index ranges	--7<=h<=8, -26<=k<=24, -9<=l<=15	-3<=h<=7, -20<=k<=20, -23<=l<=23	-9<=h<=9, -30<=k<=13, -21<=l<=21
Reflections collected	17724	21785	38566
Independent reflections	3498 [R(int) = 0.0526]	3583 [R(int) = 0.0263]	6161 [R(int) = 0.0961]
Completeness to theta = x	99.9 % (x= 69.993°)	99.3 % (x= 66.493°)	99.8 % (x= 66.499°)
Absorption correction	Semi-empirical from equivalents	Semi-empirical from equivalents	Semi-empirical from equivalents
Max. and min. transmission	0.4685 and 0.0831	0.1094 and 0.0333	0.7129 and 0.0950
Refinement method	Full-matrix least-squares on F^2	Full-matrix least-squares on F^2	Full-matrix least-squares on F^2
Data / restraints / parameters	3498 / 0 / 214	3583 / 0 / 259	6161 / 6 / 428
Goodness-of-fit on F^2	1.071	1.078	0.970
Final R indices [I>2sigma(I)]	R_1 = 0.0620, wR_2 = 0.1690	R_1 = 0.0407, wR_2 = 0.1214	R_1 = 0.0848, wR_2 = 0.2267
R indices (all data)	R_1 = 0.0835, wR_2 = 0.1857	R_1 = 0.0415, wR_2 = 0.1224	R_1 = 0.1271, wR_2 = 0.2535
Extinction coefficient	n/a	n/a	n/a
Largest diff. peak and hole	0.312 and -0.295 e.$Å^{-3}$	0.382 and -0.377 e.$Å^{-3}$	0.722 and -0.419 e.$Å^{-3}$

Molecular structures of the title compounds

1,3,8,10-Tetrakis((trimethylsilyl)oxy)dibenzo-[*cd*,*lm*]-perylene (**4**)

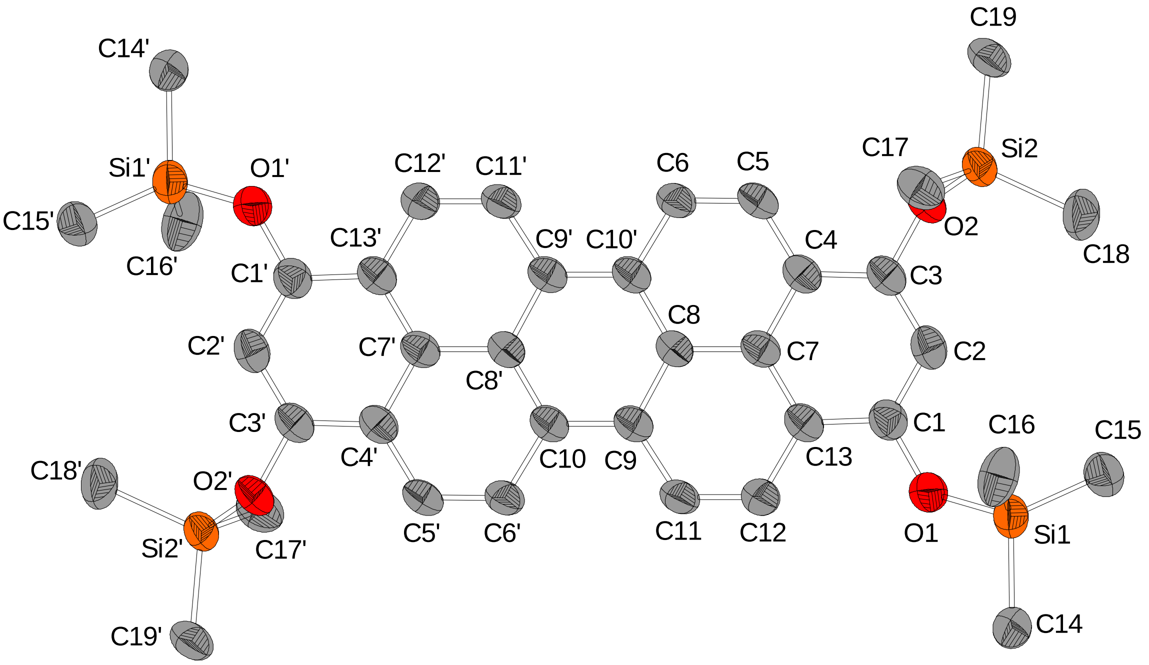

Figure S16. Reduced cell of the crystallographically determined molecular structure of **4**. Protons are not shown. Symmetry transformations: 1 -x,-y+1,-z+1.

Table S7. Bond lengths [Å] and angles [°] **4**.

Si(1)-O(1)	1.671(2)
Si(1)-C(14)	1.845(3)
Si(1)-C(16)	1.857(4)
Si(1)-C(15)	1.859(3)
C(1)-O(1)	1.369(4)
C(1)-C(2)	1.382(4)
C(1)-C(13)	1.406(4)
O(2)-C(3)	1.373(3)
O(2)-Si(2)	1.660(2)
Si(2)-C(19)	1.849(3)
Si(2)-C(18)	1.855(3)
Si(2)-C(17)	1.855(3)
C(2)-C(3)	1.384(5)
C(2)-H(2)	0.9300
C(5)-C(6)	1.362(4)
C(5)-C(4)	1.416(4)
C(5)-H(5)	0.9300
C(4)-C(3)	1.403(4)
C(4)-C(7)	1.427(4)
C(6)-C(10)#1	1.440(4)
C(6)-H(6)	0.9300
C(7)-C(13)	1.420(4)
C(7)-C(8)	1.438(4)
C(8)-C(10)#1	1.420(4)
C(8)-C(9)	1.430(4)
C(9)-C(10)	1.415(4)
C(9)-C(11)	1.432(4)
C(11)-C(12)	1.358(4)
C(11)-H(11)	0.9300
C(12)-C(13)	1.432(4)
C(12)-H(12)	0.9300

C(14)-H(14A)	0.9600
C(14)-H(14B)	0.9600
C(14)-H(14C)	0.9600
C(15)-H(15A)	0.9600
C(15)-H(15B)	0.9600
C(15)-H(15C)	0.9600
C(16)-H(16A)	0.9600
C(16)-H(16B)	0.9600
C(16)-H(16C)	0.9600
C(17)-H(17A)	0.9600
C(17)-H(17B)	0.9600
C(17)-H(17C)	0.9600
C(18)-H(18A)	0.9600
C(18)-H(18B)	0.9600
C(18)-H(18C)	0.9600
C(19)-H(19A)	0.9600
C(19)-H(19B)	0.9600
C(19)-H(19C)	0.9600
O(1)-Si(1)-C(14)	103.38(14)
O(1)-Si(1)-C(16)	108.61(14)
C(14)-Si(1)-C(16)	112.24(19)
O(1)-Si(1)-C(15)	110.91(15)
C(14)-Si(1)-C(15)	110.54(16)
C(16)-Si(1)-C(15)	110.91(18)
O(1)-C(1)-C(2)	121.5(3)
O(1)-C(1)-C(13)	117.1(3)
C(2)-C(1)-C(13)	121.4(3)
C(1)-O(1)-Si(1)	128.5(2)
C(3)-O(2)-Si(2)	127.1(2)
O(2)-Si(2)-C(19)	104.20(14)
O(2)-Si(2)-C(18)	110.49(15)
C(19)-Si(2)-C(18)	110.15(18)
O(2)-Si(2)-C(17)	107.37(14)
C(19)-Si(2)-C(17)	112.95(16)
C(18)-Si(2)-C(17)	111.40(19)
C(1)-C(2)-C(3)	120.0(3)
C(1)-C(2)-H(2)	120.0
C(3)-C(2)-H(2)	120.0
C(6)-C(5)-C(4)	121.5(3)
C(6)-C(5)-H(5)	119.3
C(4)-C(5)-H(5)	119.3
C(3)-C(4)-C(5)	122.4(3)
C(3)-C(4)-C(7)	118.2(3)
C(5)-C(4)-C(7)	119.3(3)
O(2)-C(3)-C(2)	120.5(3)
O(2)-C(3)-C(4)	117.8(3)
C(2)-C(3)-C(4)	121.6(3)
C(5)-C(6)-C(10)#1	121.4(3)
C(5)-C(6)-H(6)	119.3
C(10)#1-C(6)-H(6)	119.3
C(13)-C(7)-C(4)	120.2(3)
C(13)-C(7)-C(8)	120.4(3)
C(4)-C(7)-C(8)	119.3(3)
C(10)#1-C(8)-C(9)	120.5(3)
C(10)#1-C(8)-C(7)	120.2(3)
C(9)-C(8)-C(7)	119.3(3)
C(10)-C(9)-C(8)	119.3(3)
C(10)-C(9)-C(11)	122.5(3)
C(8)-C(9)-C(11)	118.3(3)
C(9)-C(10)-C(8)#1	120.2(3)
C(9)-C(10)-C(6)#1	121.4(3)
C(8)#1-C(10)-C(6)#1	118.3(3)
C(12)-C(11)-C(9)	122.5(3)
C(12)-C(11)-H(11)	118.8
C(9)-C(11)-H(11)	118.8
C(11)-C(12)-C(13)	120.6(3)

C(11)-C(12)-H(12)	119.7
C(13)-C(12)-H(12)	119.7
C(1)-C(13)-C(7)	118.5(3)
C(1)-C(13)-C(12)	122.6(3)
C(7)-C(13)-C(12)	118.9(3)
Si(1)-C(14)-H(14A)	109.5
Si(1)-C(14)-H(14B)	109.5
H(14A)-C(14)-H(14B)	109.5
Si(1)-C(14)-H(14C)	109.5
H(14A)-C(14)-H(14C)	109.5
H(14B)-C(14)-H(14C)	109.5
Si(1)-C(15)-H(15A)	109.5
Si(1)-C(15)-H(15B)	109.5
H(15A)-C(15)-H(15B)	109.5
Si(1)-C(15)-H(15C)	109.5
H(15A)-C(15)-H(15C)	109.5
H(15B)-C(15)-H(15C)	109.5
Si(1)-C(16)-H(16A)	109.5
Si(1)-C(16)-H(16B)	109.5
H(16A)-C(16)-H(16B)	109.5
Si(1)-C(16)-H(16C)	109.5
H(16A)-C(16)-H(16C)	109.5
H(16B)-C(16)-H(16C)	109.5
Si(2)-C(17)-H(17A)	109.5
Si(2)-C(17)-H(17B)	109.5
H(17A)-C(17)-H(17B)	109.5
Si(2)-C(17)-H(17C)	109.5
H(17A)-C(17)-H(17C)	109.5
H(17B)-C(17)-H(17C)	109.5
Si(2)-C(18)-H(18A)	109.5
Si(2)-C(18)-H(18B)	109.5
H(18A)-C(18)-H(18B)	109.5
Si(2)-C(18)-H(18C)	109.5
H(18A)-C(18)-H(18C)	109.5
H(18B)-C(18)-H(18C)	109.5
Si(2)-C(19)-H(19A)	109.5
Si(2)-C(19)-H(19B)	109.5
H(19A)-C(19)-H(19B)	109.5
Si(2)-C(19)-H(19C)	109.5
H(19A)-C(19)-H(19C)	109.5
H(19B)-C(19)-H(19C)	109.5

Symmetry transformations used to generate equivalent atoms:
#1 -x,-y+1,-z+1

1,3,10,12-tetrakis((trimethylsilyl)oxy)benzo[rst]dinaphtho[8,1,2-*cde*:2',1',8'-*klm*]pentaphene (13)

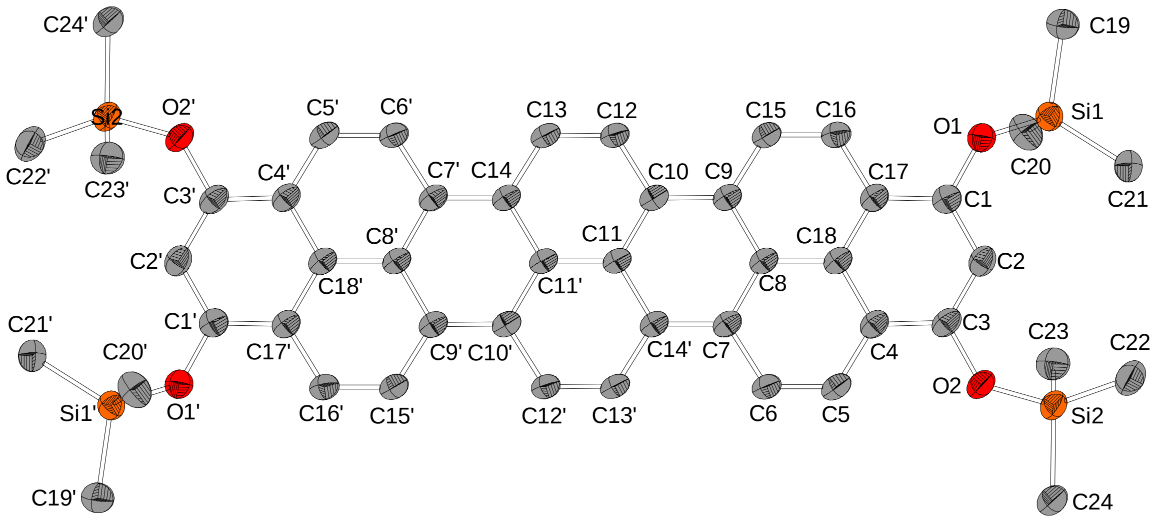

Figure S17. Reduced cell of the crystallographically determined molecular structure of **13**. Protons are not shown. Symmetry transformations: 1 -x,-y+1,-z+1.

Table S8. Bond lengths [Å] and angles [°] **13**.

Si(1)-O(1)	1.6715(12)
Si(1)-C(19)	1.847(2)
Si(1)-C(20)	1.854(2)
Si(1)-C(21)	1.862(2)
Si(2)-O(2)	1.6721(12)
Si(2)-C(24)	1.8506(19)
Si(2)-C(23)	1.856(2)
Si(2)-C(22)	1.859(2)
O(1)-C(1)	1.369(2)
O(2)-C(3)	1.377(2)
C(1)-C(2)	1.394(2)
C(1)-C(17)	1.405(2)
C(2)-C(3)	1.383(3)
C(3)-C(4)	1.401(2)
C(4)-C(5)	1.425(2)
C(4)-C(18)	1.428(2)
C(5)-C(6)	1.350(2)
C(6)-C(7)	1.439(2)
C(7)-C(14)#1	1.418(2)
C(7)-C(8)	1.420(2)
C(8)-C(9)	1.429(2)
C(8)-C(18)	1.437(2)
C(9)-C(10)	1.421(2)
C(9)-C(15)	1.431(2)
C(10)-C(11)	1.423(2)
C(10)-C(12)	1.433(2)
C(11)-C(14)#1	1.430(2)
C(11)-C(11)#1	1.436(3)
C(12)-C(13)	1.351(2)
C(13)-C(14)	1.427(2)
C(15)-C(16)	1.351(2)
C(16)-C(17)	1.431(2)
C(17)-C(18)	1.417(2)
O(1)-Si(1)-C(19)	104.13(8)
O(1)-Si(1)-C(20)	107.98(8)
C(19)-Si(1)-C(20)	112.11(9)
O(1)-Si(1)-C(21)	112.81(8)

C(19)-Si(1)-C(21)	110.02(9)
C(20)-Si(1)-C(21)	109.71(10)
O(2)-Si(2)-C(24)	104.93(8)
O(2)-Si(2)-C(23)	108.82(8)
C(24)-Si(2)-C(23)	112.20(9)
O(2)-Si(2)-C(22)	110.69(9)
C(24)-Si(2)-C(22)	109.04(9)
C(23)-Si(2)-C(22)	110.99(11)
C(1)-O(1)-Si(1)	127.30(10)
C(3)-O(2)-Si(2)	126.46(11)
O(1)-C(1)-C(2)	120.88(16)
O(1)-C(1)-C(17)	118.42(15)
C(2)-C(1)-C(17)	120.65(16)
C(3)-C(2)-C(1)	120.25(16)
O(2)-C(3)-C(2)	120.98(15)
O(2)-C(3)-C(4)	117.52(16)
C(2)-C(3)-C(4)	121.49(15)
C(3)-C(4)-C(5)	122.63(15)
C(3)-C(4)-C(18)	118.26(16)
C(5)-C(4)-C(18)	119.09(15)
C(6)-C(5)-C(4)	121.23(15)
C(5)-C(6)-C(7)	121.97(16)
C(14)#1-C(7)-C(8)	120.01(15)
C(14)#1-C(7)-C(6)	121.83(16)
C(8)-C(7)-C(6)	118.16(15)
C(7)-C(8)-C(9)	120.36(15)
C(7)-C(8)-C(18)	120.14(15)
C(9)-C(8)-C(18)	119.49(15)
C(10)-C(9)-C(8)	119.40(15)
C(10)-C(9)-C(15)	122.47(15)
C(8)-C(9)-C(15)	118.13(15)
C(9)-C(10)-C(11)	120.55(15)
C(9)-C(10)-C(12)	121.73(16)
C(11)-C(10)-C(12)	117.71(15)
C(10)-C(11)-C(14)#1	119.57(15)
C(10)-C(11)-C(11)#1	120.47(18)
C(14)#1-C(11)-C(11)#1	119.96(19)
C(13)-C(12)-C(10)	121.80(16)
C(12)-C(13)-C(14)	122.42(15)
C(7)#1-C(14)-C(13)	122.28(15)
C(7)#1-C(14)-C(11)#1	120.10(16)
C(13)-C(14)-C(11)#1	117.62(15)
C(16)-C(15)-C(9)	122.39(15)
C(15)-C(16)-C(17)	120.90(16)
C(1)-C(17)-C(18)	118.89(15)
C(1)-C(17)-C(16)	122.21(16)
C(18)-C(17)-C(16)	118.89(15)
C(17)-C(18)-C(4)	120.40(15)
C(17)-C(18)-C(8)	120.20(15)
C(4)-C(18)-C(8)	119.40(16)

Symmetry transformations used to generate equivalent atoms:
#1 -x,-y+1,-z+1

1,3,10,12-tetrakis((triisopropylsilyl)oxy)benzo[*rst*]dinaphtho[8,1,2-*cde*:2',1',8'-*klm*]pentaphene (15)

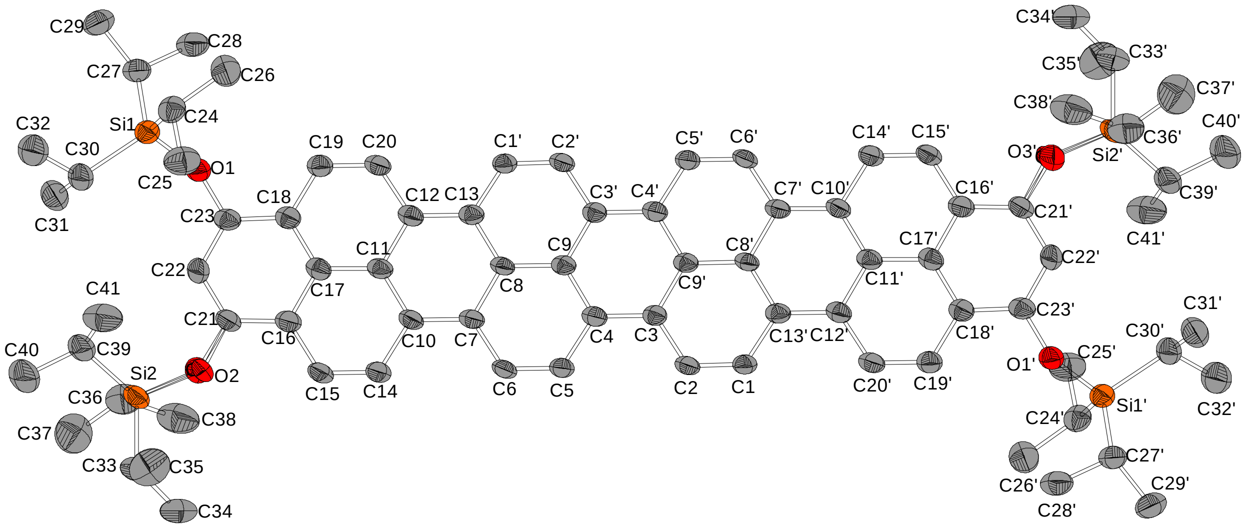

Figure S18. Reduced cell of the crystallographically determined molecular structure of **15**. Protons are not shown. Symmetry transformations: 1 -x+2,-y+1,-z+1.

Table S9. Bond lengths [Å] and angles [°] **13**.

Si(1)-O(1)	1.676(3)
Si(1)-C(24)	1.874(5)
Si(1)-C(30)	1.878(4)
Si(1)-C(27)	1.882(4)
Si(2)-O(3)	1.63(3)
Si(2)-O(2)	1.668(8)
Si(2)-C(33)	1.855(5)
Si(2)-C(39)	1.877(5)
Si(2)-C(36)	1.924(6)
O(1)-C(23)	1.366(5)
C(1)-C(2)	1.339(5)
C(1)-C(13)#1	1.430(5)
C(2)-C(3)	1.426(5)
C(3)-C(4)	1.422(6)
C(3)-C(9)#1	1.436(5)
C(4)-C(5)	1.425(5)
C(4)-C(9)	1.429(5)
C(5)-C(6)	1.343(6)
C(6)-C(7)	1.424(5)
C(7)-C(10)	1.414(6)
C(7)-C(8)	1.439(5)
C(8)-C(9)	1.426(5)
C(8)-C(13)	1.437(5)
C(10)-C(11)	1.421(6)
C(10)-C(14)	1.445(5)
C(11)-C(17)	1.432(6)
C(11)-C(12)	1.439(5)
C(12)-C(13)	1.415(6)
C(12)-C(20)	1.429(6)
C(14)-C(15)	1.343(6)
C(15)-C(16)	1.430(6)
C(16)-C(21)	1.393(6)
C(16)-C(17)	1.430(5)
C(17)-C(18)	1.411(6)
C(18)-C(23)	1.408(6)
C(18)-C(19)	1.434(6)
C(19)-C(20)	1.343(6)

C(21)-O(2)	1.382(7)
C(21)-C(22)	1.387(6)
C(21)-O(3)	1.40(2)
C(22)-C(23)	1.391(6)
C(24)-C(26)	1.541(6)
C(24)-C(25)	1.541(7)
C(27)-C(28)	1.527(6)
C(27)-C(29)	1.534(6)
C(30)-C(31)	1.526(7)
C(30)-C(32)	1.552(6)
C(33)-C(35)	1.540(8)
C(33)-C(34)	1.545(7)
C(36)-C(37)	1.484(8)
C(36)-C(38)	1.547(8)
C(39)-C(40)	1.519(6)
C(39)-C(41)	1.549(8)
O(1)-Si(1)-C(24)	111.05(19)
O(1)-Si(1)-C(30)	109.98(18)
C(24)-Si(1)-C(30)	107.9(2)
O(1)-Si(1)-C(27)	101.21(18)
C(24)-Si(1)-C(27)	113.7(2)
C(30)-Si(1)-C(27)	112.9(2)
O(3)-Si(2)-C(33)	118.5(18)
O(2)-Si(2)-C(33)	105.6(3)
O(3)-Si(2)-C(39)	119.2(8)
O(2)-Si(2)-C(39)	109.9(4)
C(33)-Si(2)-C(39)	112.5(2)
O(3)-Si(2)-C(36)	80(3)
O(2)-Si(2)-C(36)	107.2(5)
C(33)-Si(2)-C(36)	113.2(3)
C(39)-Si(2)-C(36)	108.4(3)
C(23)-O(1)-Si(1)	134.5(3)
C(2)-C(1)-C(13)#1	122.7(4)
C(1)-C(2)-C(3)	122.4(4)
C(4)-C(3)-C(2)	122.5(4)
C(4)-C(3)-C(9)#1	120.1(4)
C(2)-C(3)-C(9)#1	117.4(4)
C(3)-C(4)-C(5)	122.1(4)
C(3)-C(4)-C(9)	120.4(4)
C(5)-C(4)-C(9)	117.5(4)
C(6)-C(5)-C(4)	122.4(4)
C(5)-C(6)-C(7)	122.2(4)
C(10)-C(7)-C(6)	122.8(4)
C(10)-C(7)-C(8)	119.6(4)
C(6)-C(7)-C(8)	117.6(4)
C(9)-C(8)-C(13)	120.6(4)
C(9)-C(8)-C(7)	119.8(4)
C(13)-C(8)-C(7)	119.6(4)
C(8)-C(9)-C(4)	120.5(4)
C(8)-C(9)-C(3)#1	120.0(4)
C(4)-C(9)-C(3)#1	119.5(4)
C(7)-C(10)-C(11)	121.0(4)
C(7)-C(10)-C(14)	121.7(4)
C(11)-C(10)-C(14)	117.3(4)
C(10)-C(11)-C(17)	120.9(4)
C(10)-C(11)-C(12)	119.6(4)
C(17)-C(11)-C(12)	119.5(4)
C(13)-C(12)-C(20)	122.4(4)
C(13)-C(12)-C(11)	119.9(4)
C(20)-C(12)-C(11)	117.7(4)
C(12)-C(13)-C(1)#1	122.9(4)
C(12)-C(13)-C(8)	120.3(4)
C(1)#1-C(13)-C(8)	116.8(4)
C(15)-C(14)-C(10)	122.3(4)
C(14)-C(15)-C(16)	121.3(4)
C(21)-C(16)-C(15)	122.5(4)

C(21)-C(16)-C(17)	118.6(4)
C(15)-C(16)-C(17)	118.9(4)
C(18)-C(17)-C(16)	120.5(4)
C(18)-C(17)-C(11)	120.4(4)
C(16)-C(17)-C(11)	119.1(4)
C(23)-C(18)-C(17)	118.7(4)
C(23)-C(18)-C(19)	122.6(4)
C(17)-C(18)-C(19)	118.7(4)
C(20)-C(19)-C(18)	121.2(4)
C(19)-C(20)-C(12)	122.5(4)
O(2)-C(21)-C(22)	123.4(4)
O(2)-C(21)-C(16)	115.0(5)
C(22)-C(21)-C(16)	121.1(4)
C(22)-C(21)-O(3)	113(2)
C(16)-C(21)-O(3)	120.0(9)
C(21)-C(22)-C(23)	120.4(4)
O(1)-C(23)-C(22)	120.9(4)
O(1)-C(23)-C(18)	118.4(4)
C(22)-C(23)-C(18)	120.7(4)
C(26)-C(24)-C(25)	109.8(4)
C(26)-C(24)-Si(1)	113.8(3)
C(25)-C(24)-Si(1)	112.5(3)
C(28)-C(27)-C(29)	109.2(4)
C(28)-C(27)-Si(1)	114.1(3)
C(29)-C(27)-Si(1)	113.4(3)
C(31)-C(30)-C(32)	109.6(4)
C(31)-C(30)-Si(1)	112.8(3)
C(32)-C(30)-Si(1)	115.2(3)
C(35)-C(33)-C(34)	110.1(5)
C(35)-C(33)-Si(2)	112.9(4)
C(34)-C(33)-Si(2)	112.4(4)
C(37)-C(36)-C(38)	109.7(5)
C(37)-C(36)-Si(2)	115.8(4)
C(38)-C(36)-Si(2)	111.9(4)
C(40)-C(39)-C(41)	107.7(4)
C(40)-C(39)-Si(2)	114.5(4)
C(41)-C(39)-Si(2)	113.6(4)
C(21)-O(2)-Si(2)	131.7(7)
C(21)-O(3)-Si(2)	133(4)

Symmetry transformations used to generate equivalent atoms:
#1 -x+2,-y+1,-z+1

Experimental

*1,4-bis(4,4,5,5-tetramethyl-1,3,2-dioxaborolan-2-yl)naphthalene (**6**)*

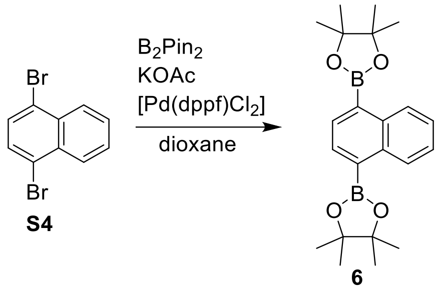

According to a literature known procedure[18], 1.00 g (3.51 mmol, 1.0 eq) 1,4-dibromonaphthalene (**S4**), 1.80 g (18.30 mmol, 5.2 eq) dry KOAc, 2.05 g (8.11 mmol, 2.3 eq) bis(pinacolato)diboron and 75 mg (0.10 mmol, 3 mol%) [Pd(dppf)Cl_2] were evacuated and back-flushed with argon in a flame-dried Schlenk flask. Thereto, 25 mL anhydrous dioxane were added and the mixture stirred for 2 d at 100 °C under argon atmosphere. After cooling to room temperature, 20 mL water and 20 mL ethyl acetate were added and the organic phase was separated. The aqueous phase was extracted with ethyl acetate (2x 30 mL). The combined organic phases were dried over MgSO4 and the crude product was purified by column chromatography on silica (*n*-hexane/ethyl acetate 20:1). 1.05 g (2.76 mmol, 79 %) of **6** were obtained as colorless solid. ^{1}H NMR (300.1 MHz, $CDCl_3$): δ_H = 1.42 (s, 24H, 4 x C(CH_3)$_2$), 7.48-7.54 (m, 2H), 8.02 (s, 2H), 8.72-8.78 (m, 2H) ppm. ^{13}C NMR (75.5 MHz, $CDCl_3$): δ_C = 25.1, 83.9, 126.0, 128.8, 134.4, 136.7 ppm. The analytical data are in accordance with the literature.[18]

*4,4'-dibromo-1,1'-binaphthyl (**S10**)*

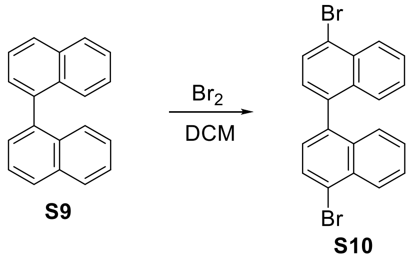

According to a literature known procedure[19], 1.00 g (3.94 mmol, 1.0 eq) 1,1'-binaphthyl (**S9**) were dissolved in 30 mL DCM and cooled to 0°C. Under exclusion of light, 0.8 mL (15.7 mmol, 4.0 eq) bromine were added dropwise over the course of 15 min at 0 °C and stirred for further 2 h at room temperature. To this, 30 mL *n*-pentane were added and a colorless solid was formed. The suspension was filtered and the solid was washed with n-pentane (20 mL) and dried in vacuo. 1.39 g (3.39 mmol, 86 %) of **S10** were obtained as off-white solid. ^{1}H NMR (300.1 MHz, $CDCl_3$): δ_H = 7.28-7.35 (m, 6H), 7.55-7.65 (m, 2H), 7.90 (d, $^3J_{H,H}$ = 7.6 Hz, 2H), 8.36 (d, $^3J_{H,H}$ = 7.6 Hz, 2H) ppm. ^{13}C NMR (75.5 MHz, $CDCl_3$): δ_C = 123.2, 127.1, 127.1, 127.5, 127.6, 128.3, 129.6, 132.1, 134.1, 137.8 ppm. The analytical data are in accordance with the literature.[19]

*4,4'-bis(4,4,5,5-tetramethyl-1,3,2-dioxaborolan-2-yl)-1,1'-binaphthalene (**7**)*

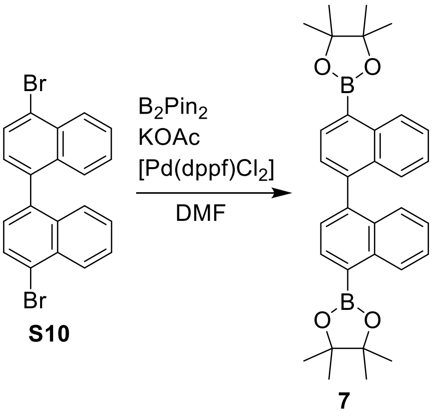

According to a literature known procedure[19], 1.35 g (3.30 mmol, 1.0 eq) 4,4'-dibromo-1,1'-binaphthyl (**S10**), 1.95 g (20.00 mmol, 6.9 eq) dry KOAc, 2.09 g (8.20 mmol, 2.5 eq) bis(pinacolato)diboron and 75 mg (0.10 mmol, 3 mol%) [Pd(dppf)Cl_2] were evacuated and back-flushed with argon in a flame-dried Schlenk flask. Thereto, 15 mL anhydrous DMF were added and the mixture stirred for 18 at 100 °C under argon atmosphere. After cooling to room temperature, 20 mL methanol were added and the precipitate filtered. The resulting solid was washed with water and methanol (20 mL) and recrystallized in THF/n-hexane. 1.32 g (2.61 mmol, 79 %) of **7** were obtained as off-white solid. ^{1}H NMR (300.1 MHz, $CDCl_3$): δ_H = 1.46 (s, 24H, 4 x C(CH_3)$_2$), 7.24 (t, $^3J_{H,H}$ = 7.5 Hz, 2H), 7.36 (d, $^3J_{H,H}$ = 8.2 Hz, 2H), 7.45-7.55 (m, 4H), 8.19 (d, $^3J_{H,H}$ = 7.0 Hz, 2H), 8.87 (d, $^3J_{H,H}$ = 8.4 Hz, 2H) ppm. ^{13}C NMR (75.5 MHz, $CDCl_3$): δ_C = 27.1, 84.0, 125.7, 126.4, 126.9, 127.0, 128.7, 132.6, 135.2, 137.1, 142.3 ppm. The analytical data are in accordance with the literature.[19]

*4,4''-dibromo-1,1':4',1''-ternaphthalene (**S5**)*

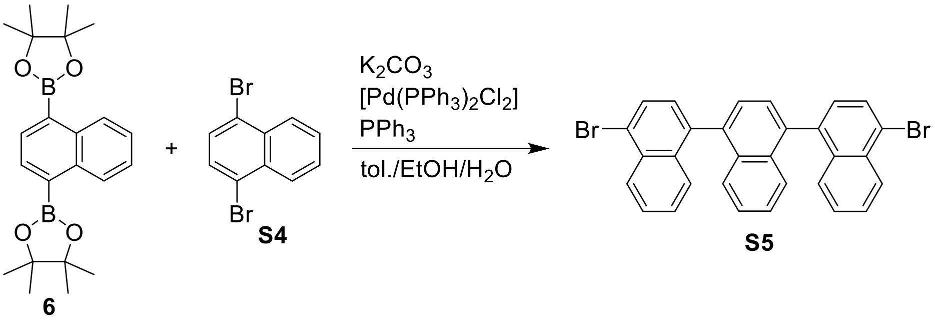

In a Schlenk flask, 1.25 g (4.30 mmol, 3.0 eq) 1,4-dibromonaphthalene (**S4**), 545 mg (1.43 mmol, 1.0 eq) **6** and 1.19 g (8.60 mmol, 6.0 eq) K_2CO_3 were dissolved in 40 mL toluene, 10 mL ethanol and 5 mL water and subsequently degassed. Under argon atmosphere, 50 mg (0.07 mmol, 5 mol%) [Pd(PPh3)2Cl2] and 36 mg (0.14 mmol, 10 mol%) triphenyl phosphine were added and it was refluxed for 18 h. After cooling to room temperature, 20 mL water were added and the organic phase was separated. The aqueous phase was extracted with ethyl acetate (2x 30 mL). The combined organic phases were dried over $MgSO_4$ and the crude product was purified by column chromatography on silica (*n*-hexane/chloroform 20:1). 305 mg (0.57 mmol, 40 %) of **S5** were obtained as colorless solid. ^{1}H NMR (300.1 MHz, $CDCl_3$): δ_H = 7.25-7.32 (m, 2H), 7.38-7.70 (m, 12H), 7.78 (dd, $^3J_{H,H}$ = 7.6, Hz, $^4J_{H,H}$ = 3.6 Hz, 2H), 8.39 (d, $^3J_{H,H}$ = 8.6 Hz, 2H) ppm. ^{13}C NMR (75.5 MHz, $CDCl_3$): δ_C = 123.0, 126.5, 126.6, 127.0, 127.3, 127.4, 127.6, 127.6, 127.8, 127.8, 128.8, 130.0, 132.4, 132.2, 134.6, 138.2, 139.0 ppm. The analytical data are in accordance with the literature.[19]

*4,4''-bis(4,4,5,5-tetramethyl-1,3,2-dioxaborolan-2-yl)-1,1':4',1''-ternaphthalene (**S6**)*

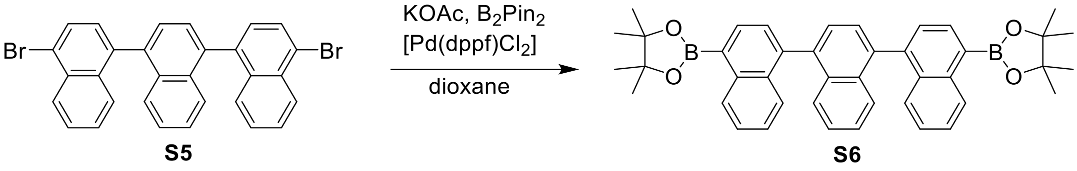

According to a literature known procedure[19], 300 mg (0.56 mmol, 1.0 eq) 4,4''-dibromo-1,1':4',1''-ternaphthalene (**S5**), 330 mg (3.36 mmol, 6.0 eq) dry KOAc, 427 mg (1.68 mmol, 3.0 eq) bis(pinacolato)diboron and 20 mg (0.03 mmol, 3 mol%) [Pd(dppf)Cl_2] were evacuated and back-flushed with argon in a flame-dried Schlenk flask. Thereto, 20 mL anhydrous dioxane were added and the mixture stirred for 18 h at 100 °C under argon atmosphere. After cooling to room temperature, 20 mL water and 20 mL ethyl acetate were added and the organic phase was separated. The aqueous phase was extracted with ethyl acetate (2x 30 mL). The combined organic phases were dried over MgSO4 and the crude product was purified by column chromatography on silica (*n*-hexane/ethyl acetate 4:1). 225 mg (0.36 mmol, 64 %) of **S6** were obtained as colorless solid, from which excess of bis(pinacolato)diboron was removed by washing with diethyl ether (10 mL). ^{1}H NMR (300.1 MHz, CD_2Cl_2): δ_H = 1.47 (s, 24H, 4 x C(CH_3)$_2$), 7.22-7.28 (m, 2H), 7.30-7.45 (m, 4H), 7.49-7.66 (m, 8H), 8.21 (dd, $^3J_{H,H}$ = 7.0, Hz, $^4J_{H,H}$ = 3.5 Hz, 2H) 8.89 (dd, $^3J_{H,H}$ = 8.8 Hz, $^4J_{H,H}$ = 2.2 Hz, 2H) ppm. ^{13}C NMR (75.5 MHz, CD_2Cl_2): δ_C = 25.2, 84.4, 126.1, 126.3, 126.6, 127.1, 127.3, 127.5, 127.6, 129.2, 133.1, 133.2, 135.5, 137.5, 138.9, 142.4 ppm. The analytical data are in accordance with the literature.[19]

*6-bromo-3-hydroxy-1H-phenalen-1-one (**8**)*

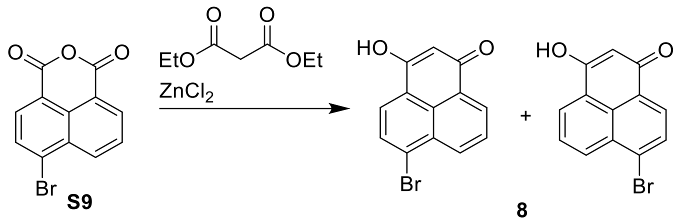

S9 8

In a flame dried Schlenk flask, 6 mL diethylmalonate were added to 2.00 g (7.20 mmol, 1.0 eq) 6-bromo naphthalene anhydride (**S9**) and 2.00 g (14.40 mmol, 2.0 eq) dry $ZnCl_2$ under argon atmosphere. The suspension was stirred at 185 °C for 6 h, until the gas evolution ceased and the red mixture solidified. 200 mL 2M aqueous KOH were added and the red solution filtered. The filtrate was cooled with an ice bath and 200 mL 2M acetic acid were added carefully. The resulting yellow to brown precipitate was collected by filtration, washed with water and dried at 120 °C overnight. 1.60 g **8** (5.85 mmol, 81 %) were obtained as brown solid. ^{1}H NMR (300.1 MHz, DMSO-d_6): δ_H = 5.38 (s, 1H), 7.72 (t, $^3J_{H,H}$ = 7.8 Hz, 1H), 7.92 (d, $^3J_{H,H}$ = 7.8 Hz, 1H), 7.99 (d, $^3J_{H,H}$ = 7.7 Hz, 1H), 8.20 (dd, $^3J_{H,H}$ = 8.3 Hz, $^4J_{H,H}$ = 4.2 Hz, 2H) ppm. ^{13}C NMR (75.5 MHz, DMSO-d_6): δ_C = 105.3, 127.9, 128.2, 129.1, 130.1, 130.8, 131.5, 131.6, 132.5, 133.2, 133.6 ppm (C-O not detected). HR-MS (APCI-) m/z clcd. for $[C_{13}H_6BrO_2]^-$: 279.9537 (found: 279.9545). IR (ATR), ṽ = 3019 (w), 2216 (w), 2170 (w), 1958 (w), 1622 (s), 1554 (vs), 1338 (m), 1254 (m), 1212 (m), 1024 (w), 942 (w), 805 (m), 768 (m), 649 (w), 521 (w) cm^{-1}.
Note: **8** consists of two possible inseparable constitutional isomers leading to broadened ^{1}H NMR signals.

*6-bromo-2-butyl-3-hydroxy-1H-phenalen-1-one (**S1**)*

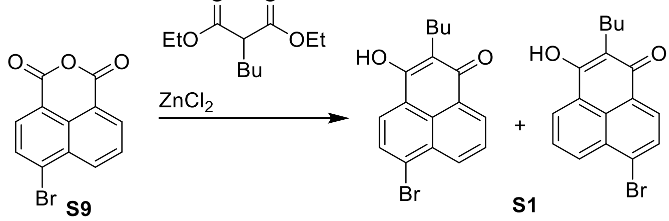

S9 S1

In a flame dried Schlenk flask, 5 mL diethyl butylmalonate were added to 1.00 g (3.60 mmol, 1.0 eq) 6-bromo naphthalene anhydride (**S9**) and 1.00 g (7.20 mmol, 2.0 eq) dry $ZnCl_2$ under argon atmosphere. The suspension was stirred at 185 °C for 4 h, until the gas evolution ceased and the red mixture solidified. The solid was taken up with a mixture of chloroform and ethyl acetate (250 mL) and washed with 250 mL water and 250 mL brine. The organic layer was separated and dried over Na_2SO_4 and filtered through a plug of neutral aluminum oxide, which was rinsed with 100 mL acetone. The solvent was concentrated to 20 mL and 100 mL *n*-pentane were added. Pure product crystalized at -20 °C from this mixture. 510 mg **S1** (1.55 mmol, 43 %) were obtained as orange solid. ^{1}H NMR (300.1 MHz, DMSO-d_6): δ_H = 0.90 (t, $^3J_{H,H}$ = 7.0 Hz, 3H, Bu*H*), 1.40-1.48 (m, 4H, Bu*H*), 2.62 (t, $^3J_{H,H}$ = 7.3 Hz, 2H, Bu*H*), 7.87 (t, $^3J_{H,H}$ = 7.9 Hz, 1H), 8.09 (d, $^3J_{H,H}$ = 7.9 Hz, 1H), 8.17 (d, $^3J_{H,H}$ = 7.9 Hz, 1H), 8.39 (dd, $^3J_{H,H}$ = 7.1 Hz, $^4J_{H,H}$ = 2.5 Hz, 2H), 10.55 (s, 1H, O*H*) ppm. ^{13}C NMR (75.5 MHz, DMSO-d_6): δ_C = 14.3, 22.6, 30.9, 118.5, 127.0, 127.1, 127.7, 128.0, 128.5, 128.6, 130.1, 131.1 ppm (C-O not detected). HR-MS (APCI-) m/z clcd. for $[C_{17}H_{14}BrO_2]^-$: 331.0164 (found: 331.0180). IR (ATR), ṽ = 3079 (w), 2950 (m), 2921 (m), 2861 (w), 1688 (w), 1616 (m), 1550 (vs), 1457 (w), 1367 (w), 1172 (s), 1104 (m), 1019 (m), 845 (w), 773 (w), 693 (w), 560 (w) cm^{-1}.
Note: **S1** consists of two possible inseparable constitutional isomers leading to broadened ^{1}H NMR signals.

*3-hydroxy-7-(4-(3-hydroxy-1-oxo-1H-phenalen-6-yl)naphthalen-1-yl)-1H-phenalen-1-one (**9**)*

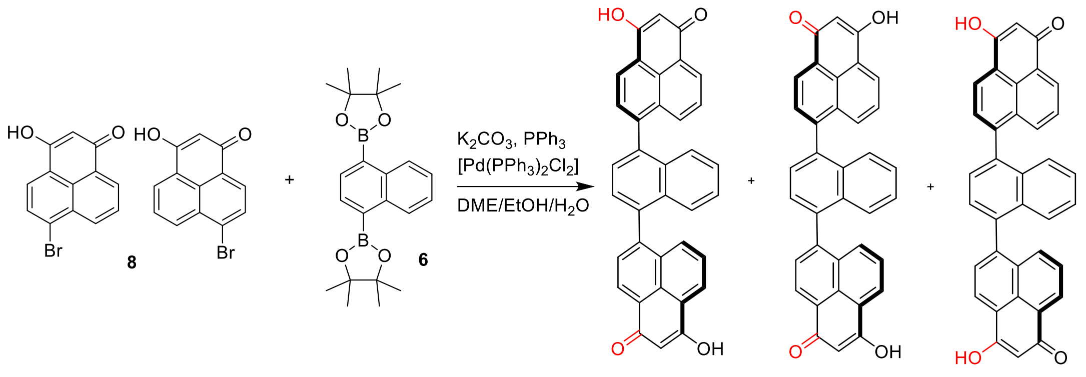

9 (mixture of isomers)

In a Schlenk flask, 417 mg (1.09 mmol, 1.0 eq) boronic acid pinacol ester **6**, 725 mg (2.65 mmol, 2.4 eq) 6-bromo-3-hydroxy-1*H*-phenalen-1-one (**8**, isomer mixture) and 1.83 g (13.2 mmol, 12.0 eq) K_2CO_3 were dissolved in 20 mL dimethoxyethan, 10 mL ethanol and 5 mL water and degassed thoroughly. Under argon atmosphere, 77 mg (0.11 mmol, 10 mol%) [$Pd(PPh_3)_2Cl_2$] and 53 mg (0.20 mmol, 20 mol%) triphenylphosphine were added and the reaction mixture was heated to reflux for 18 h. After cooling down to room temperature, 2 M acetic acid (50 mL) was added and a yellow precipitate was formed. The yellow solid was collected by filtration and washed with water (50 mL), methanol (50 mL) and diethyl ether (50 mL) and dried in vacuo. 448 mg (0.87 mmol, 80 %) of the yellow solid **9** were obtained and directly used for the next step. Note: due to the insoluble nature of the product, some aromatic impurities could not be removed completely from the product. ^{1}H NMR (300.1 MHz, DMSO-d_6): δ_H = 6.10 (s, 2H), 7.30-8.10 (br m, 12H), 8.15-8.40 (br m, 4H), 11.87 (br s, 2H, O*H*) ppm. ^{13}C NMR not recorded due to low solubility. HR-MS (APCI-) m/z clcd. for $[C_{36}H_{19}O_4]^-$: 515.1289 (found: 515.1292). IR (ATR), $\tilde{v}$ = 3056 (w), 3006 (w), 1775 (m), 1736 (m), 1626 (m), 1573 (s), 1510 (m), 1362 (w), 1302 (m), 1186 (m), 1119 (m), 1021 (w), 846 (w), 750 (vs), 720 (s), 694 (vs), 541 (s), 514 (m) cm^{-1}.
Note: **9** consists of inseparable constitutional isomers leading to broadened ^{1}H NMR signals.

*3-hydroxy-7-(4'-(3-hydroxy-1-oxo-1H-phenalen-6-yl)-[1,1'-binaphthalen]-4-yl)-1H-phenalen-1-one (**10**)*

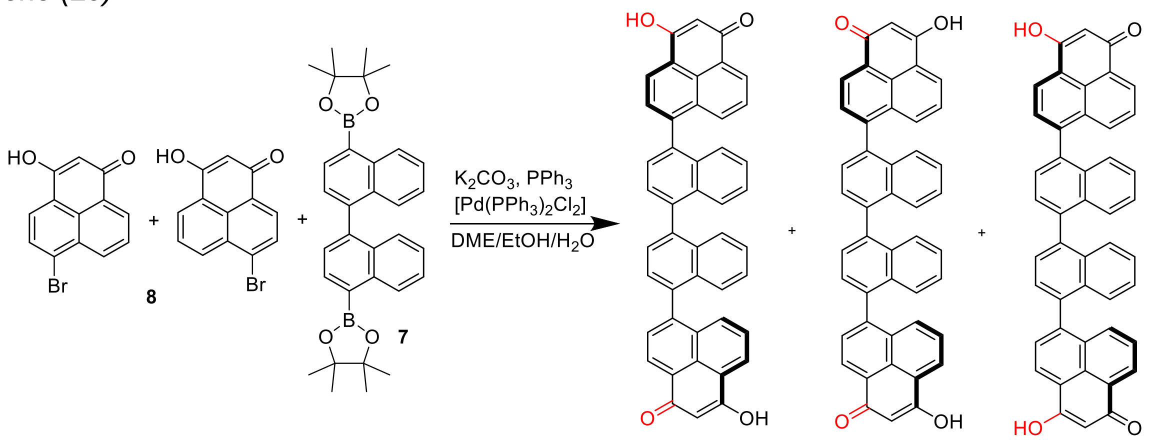

10 (mixture of isomers)

In a Schlenk flask, 600 mg (1.19 mmol, 1.0 eq) boronic acid pinacol ester **7**, 840 mg (3.09 mmol, 2.6 eq) 6-bromo-3-hydroxy-1*H*-phenalen-1-one (**8**, isomer mixture) and 1.97 g (14.3mmol, 12.0 eq) K_2CO_3 were dissolved in 30 mL dimethoxyethan, 5 mL ethanol and 5 mL water and degassed thoroughly. Under argon atmosphere, 85 mg (0.12 mmol, 10 mol%) [$Pd(PPh_3)_2Cl_2$] and 139 mg (0.53 mmol, 40 mol%) triphenylphosphine were added and the reaction mixture was heated to reflux for 18 h. After cooling down to room temperature, 2 M acetic acid (50 mL) was added and a yellow precipitate was formed. The yellow solid was collected by filtration and washed with water (50 mL), methanol (50 mL) and diethyl ether (50 mL) and dried in vacuo. 581 mg (0.90 mmol, 76 %) of the yellow solid **10** were obtained and directly used for the next step. Note: due to the insoluble nature of the product, some aromatic impurities could not be removed completely from the product. ^{1}H NMR (300.1 MHz, DMSO-d_6): δ_H = 6.11 (s, 2H), 7.05-8.05 (br m, 18H), 8.30-8.60 (br m, 4H), 11.92 (br s, 2H, O*H*) ppm. ^{13}C NMR not recorded due to low solubility. HR-MS (APCI-) m/z clcd. for $[C_{46}H_{25}O_4]^-$: 641.1758 (found: 641.1758). IR (ATR), $\tilde{v}$ = 3057 (w), 2997 (w), 2357 (w), 2149 (w), 1919 (w), 1626 (s), 1567 (vs), 1514 (s), 1481 (m), 1366 (m), 1300 (m), 1237 (m), 1204 (w), 839 (m), 764 (vs), 721 (m), 693 (m), 540 (m) cm^{-1}.
Note: **10** consists of inseparable constitutional isomers leading to broadened ^{1}H NMR signals.

*2-butyl-7-(4'-(2-butyl-3-hydroxy-1-oxo-1H-phenalen-6-yl)-[1,1'-binaphthalen]-4-yl)-3-hydroxy-1H-phenalen-1-one (**S2**)*

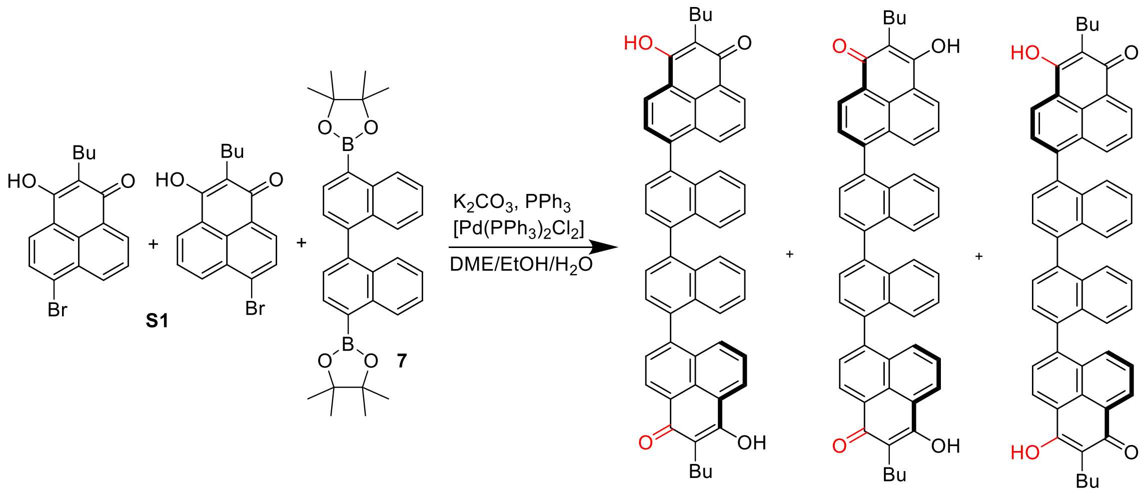

S2 (mixture of isomers)

In a Schlenk flask, 860 mg (1.70 mmol, 1.0 eq) boronic acid pinacol ester **7**, 1.35 g (4.03 mmol, 2.5 eq) 6-bromo-2-butyl-3-hydroxy-1*H*-phenalen-1-one (**S1**, isomer mixture) and 1.50 g (10.9 mmol, 6.4 eq) K_2CO_3 were dissolved in 20 mL toluene, 10 mL ethanol and 5 mL water and degassed thoroughly. Under argon atmosphere, 119 mg (0.17 mmol, 10 mol%) [Pd(PPh_3)$_2$$Cl_2$] and 89 mg (0.34 mmol, 40 mol%) triphenylphosphine were added and the reaction mixture was heated to reflux for 18 h. After cooling down to room temperature, 2 M HCl (50 mL) was added and the organic phase was separated. The aqueous phase was extracted with chloroform (2 x 50 mL). The combined organic phase was dried over $MgSO_4$ and purified via column chromatography on silica (first, side products were eluted using chloroform, product **S2** was eluted using ethyl acetate/acetone 10:1). 480 mg (0.64 mmol, 37 %) of the yellow solid **S2** were obtained. ^{1}H NMR (300.1 MHz, DMSO-d$_6$): δ_H = 0.88-1.02 (m, 6H, Bu*H*), 1.20-1.65 (m, 12H, Bu*H*), 7.20-8.05 (br m, 18H), 8.30-8.60 (br m, 4H), 10.53 (br s, 2H, O*H*) ppm. ^{13}C NMR not recorded due to low solubility. HR-MS (APCI-) m/z clcd. for [$C_{54}H_{41}O_4$]$^-$: 753.3010 (found: 753.3038). IR (ATR), ṽ = 3268 (w), 2953 (w), 2925 (m), 2868 (w), 2020 (w), 1938 (w), 1705 (w), 1621 (m), 1557 (s), 1431 (m), 1213 (m), 1187 (w), 848 (w), 719 (vs), 661 (w), 544 (w) cm^{-1}.
Note: **S2** consists of inseparable constitutional isomers leading to broadened ^{1}H NMR signals.

*2-butyl-7-(4''-(2-butyl-3-hydroxy-1-oxo-1H-phenalen-6-yl)-[1,1':4',1''-ternaphthalen]-4-yl)-3-hydroxy-1H-phenalen-1-one (**S7**)*

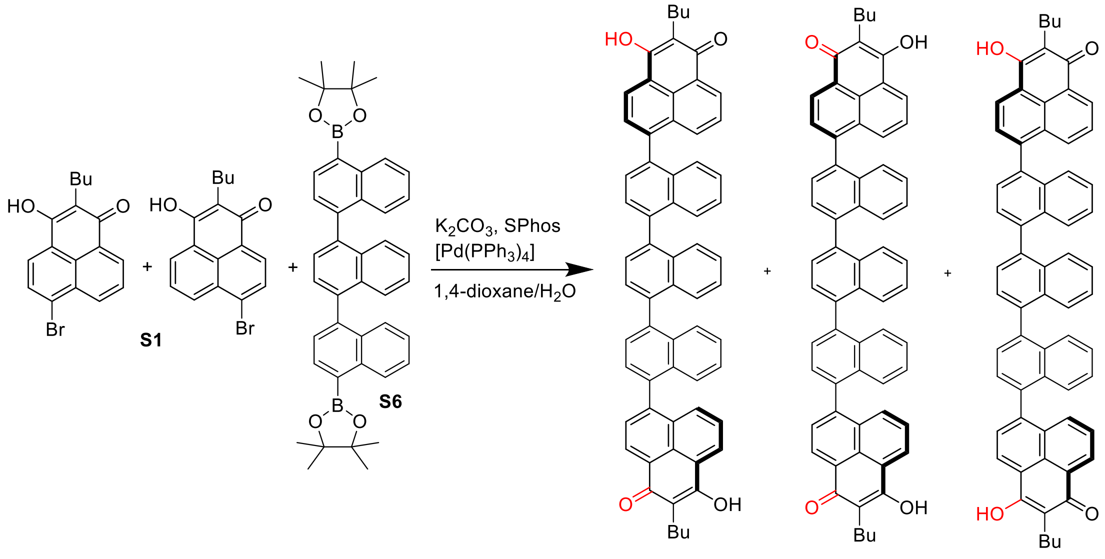

S7 (mixture of isomers)

In a Schlenk flask, 200 mg (0.32 mmol, 1.0 eq) boronic acid pinacol ester **S6**, 256 mg (0.80 mmol, 2.5 eq) 6-bromo-2-butyl-3-hydroxy-1*H*-phenalen-1-one (**S1**, isomer mixture) and 531 mg (3.84 mmol, 12.0 eq) K_2CO_3 were dissolved in 10 mL dioxane and 3 mL water and degassed thoroughly. Under argon atmosphere, 37 mg (0.03 mmol, 10 mol%) [Pd(PPh_3)$_4$] and 25 mg (0.06 mmol, 20 mol%) SPhos were added and the reaction mixture was heated to 100 °C and stirred for 3 d. After cooling down to room temperature, 2 M HCl (20 mL) was added and the organic phase was separated. The aqueous

phase was extracted with chloroform (3 x 20 mL). The combined organic phase was dried over $MgSO_4$ and filtered. The solvent was evaporated and the residue suspended in 50 mL methanol. The precipitate was collected by filtration and was washed with diethyl ether (20 mL). 196 mg (0.22 mmol, 70 %) of the yellow solid **S7** were obtained. ^{1}H NMR (300.1 MHz, DMSO-d$_6$): δ_H = 0.80-1.05 (m, 6H, Bu*H*), 1.10-1.60 (m, 12H, Bu*H*), 7.30-8.00 (br m, 24H), 8.35-8.65 (br m, 4H), 10.52 (br s, 2H, O*H*) ppm. ^{13}C NMR not recorded due to low solubility. HR-MS (APCI-) m/z clcd. for $[C_{64}H_{47}O_4]^-$: 880.3514 (found: 880.3494). IR (ATR), ṽ = 2958 (w), 2930 (w), 2108 (w), 2020 (w), 1943 (w), 1705 (w), 1616 (m), 1565 (vs), 1440 (w), 1216 (m), 1204 (w), 1189 (w), 848 (w), 719 (vs), 709 (w), 653 (w), 546 (w) cm^{-1}.
Note: **S7** consists of inseparable constitutional isomers leading to broadened ^{1}H NMR signals.

*3,8-dihydroxypyrene-1,6-dione (**3**)*

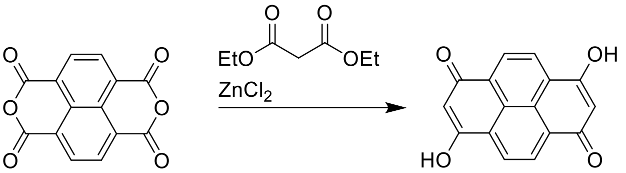

S10 **3**

In a flame dried Schlenk flask, 10 mL diethylmalonate were added to 1.00 g (3.73 mmol, 1.0 eq) naphthalene dianhydride (**S10**) and 2.00 g (14.40 mmol, 3.9 eq) dry $ZnCl_2$ under argon atmosphere. The suspension was stirred at 185 °C for 3 h, until the gas evolution ceased and the black mixture solidified. 100 mL 2M aqueous KOH were added and the brown solution filtered. The filtrate was cooled with an ice bath and 100 mL 2M acetic acid were added carefully. The resulting yellow to brown precipitate was collected by filtration, washed with water and methanol and dried at 120 °C overnight. 692 mg of **3** were obtained as brown solid. Due to the insolubility of **3**, no NMR measurements could be acquired and **3** was directly used for the next step without further purification HR-MS (APCI-) m/z clcd. for $[C_{16}H_7O_4]^-$: 263.0350 (found: 263.0357). IR (ATR), ṽ = 3064 (w), 2654 (w), 2369 (w), 2189 (w), 1676 (m), 1556 (vs), 1451 (vs), 1363 (s), 1289 (vs), 1234 (vs), 1193 (s), 1040 (m), 903 (w), 833 (m), 801 (m), 740 (w), 650 (w), 595 (w) cm^{-1}. The analytical data are in accordance to the literature.[20]

3,10-dihydroxydibenzo[cd,lm]perylene-1,8-dione (2)

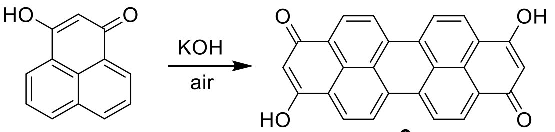

1 **2**

According to a literature known procedure[20], 1.96 g (10.00 mmol, 1.0 eq) 3-hydroxy-1H-phenalen-1-one (**1**) and 10 g mortared KOH pellets were homogenized. It was heated to 285 °C for 2 h in a Ni crucible open to air. In that time, a purple melt was formed and gas evolution ceased. After cooling to room temperature, the melt was taken up in water (100 mL), filtered and the purple filtrate was acidified with 2M acetic acid to pH = 5 and a brown precipitate was formed. The precipitate was ultrasonicated for 5 min and collected by filtration. The dark brown, insoluble solid was washed with water, methanol, THF and diethyl ether until the filtrate remains colorless. After drying at 120 °C overnight, 1.28 g of **2** were obtained as blackish-brown solid. Due to the insolubility of **2**, no NMR measurements could be acquired and it was directly used for the next step without further purification. IR (ATR), ṽ = 3174 (w), 3107 (w), 3012 (w), 2778 (w), 2256 (w), 2140 (w), 2103 (w), 2050 (w), 1921 (m), 1902 (w), 1870 (w), 1762 (w), 1585 (m), 1544 (m), 1509 (w), 1073 (vs), 955 (m), 923 (w), 803 (m), 660 (w), 591 (m), 460 (vs) cm^{-1}. The analytical data are in accordance to the literature.[20]

*3,12-dihydroxybenzo[rst]dinaphtho[8,1,2-cde:2',1',8'-klm]pentaphene-1,10-dione (**11**)*

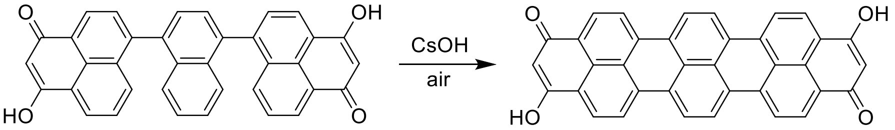

+ isomers
9 **11**

In a Ni crucible open to air, 390 mg (0.76 mmol, 1.0 eq) of **9** (isomer mixture) and 4.0 g monohydrated CsOH were homogenized. It was heated to 285 °C for 2 h on air. In that time, a purple-blue melt was formed and gas evolution ceased. After cooling to room temperature, the melt was taken up in water (100 mL), and the blue suspension was

acidified with 2M acetic acid to pH = 5 and a purple-blue precipitate was formed. The precipitate was ultrasonicated for 5 min and collected by filtration. The purple, insoluble solid was washed with water, methanol, THF and diethyl ether until the filtrate remains colorless. After drying at 120 °C overnight, 212 mg of **11** were obtained as purple solid. Due to the insolubility of **11**, no NMR measurements could be acquired and it was directly used for the next step without further purification. IR (ATR), ṽ =3669 (w), 2359 (w), 2279 (w), 2207 (m), 2169 (w), 2092 (m), 2049 (w), 1989 (m), 1961 (w), 1901 (w), 1689 (s), 1611 (vs), 1545 (s), 1497 (s), 1357 (m), 1238 (vs), 1184 (s), 999 (m), 800 (m), 711 (m), 648 (m) cm^{-1}.

*3,14-dihydroxybenzo[rst]benzo[12,1]tetrapheno[7,8,9-cde]naphtho[2,1,8-klm]pentaphene-1,12-dione (**12**)*

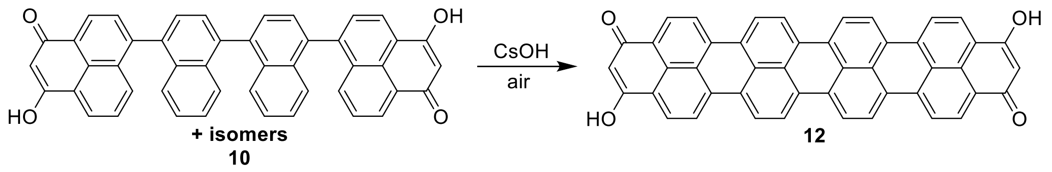

In a Ni crucible open to air, 582 mg (0.91 mmol, 1.0 eq) of **10** (isomer mixture) and 6.0 g monohydrated CsOH were homogenized. It was heated to 285 °C for 2 h on air. In that time, a blackish-blue melt was formed and gas evolution ceased. After cooling to room temperature, the melt was taken up in water (200 mL), and the blue suspension was acidified with 2M acetic acid to pH = 5 and a dark blue precipitate was formed. The precipitate was ultrasonicated for 5 min and collected by filtration. The purple, insoluble solid was washed with water, methanol, THF and diethyl ether until the filtrate remains colorless. After drying at 120 °C overnight, 429 mg of **12** were obtained as blackish-blue solid. Due to the insolubility of **12**, no NMR measurements could be acquired and it was directly used for the next step without further purification. IR (ATR), ṽ = 3860 (w), 3759 (w), 2195 (w), 2152 (w), 2078 (w), 1998 (w), 1680 (m), 1655 (w), 1605 (w), 1539 (s), 1356 (w), 1311 (w), 1299 (m), 1172 (s), 1051 (m), 1042 (w), 800 (s), 755 (w), 706 (m), 560 (w) cm^{-1}.

*2,13-dibutyl-3,14-dihydroxybenzo[rst]benzo[12,1]tetrapheno[7,8,9-cde]naphtho[2,1,8-klm]pentaphene-1,12-dione (**S3**)*

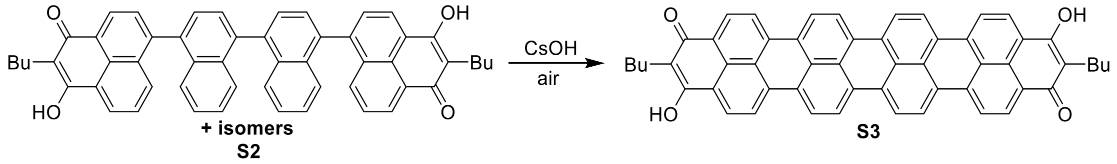

In a Ni crucible open to air, 385 mg (0.51 mmol, 1.0 eq) of **S2** (isomer mixture) and 4.0 g monohydrated CsOH were homogenized. It was heated to 285 °C for 2 h on air. In that time, a blackish-blue melt was formed and gas evolution ceased. After cooling to room temperature, the melt was taken up in water (200 mL), and the blue suspension was acidified with 2M acetic acid to pH = 5 and a dark blue precipitate was formed. The precipitate was ultrasonicated for 5 min and collected by filtration. The purple, insoluble solid was washed with water, methanol, THF and diethyl ether until the filtrate remains colorless. After drying at 120 °C overnight, 260 mg of **S3** (0.35 mmol, 68 %) were obtained as blackish-blue solid. Due to the insolubility of **S3**, no NMR measurements could be acquired and it was directly used for the next step without further purification. IR (ATR), ṽ = 3911 (w), 3860 (w), 3757 (w), 3711 (w), 2370 (w), 2198 (w), 2151 (w), 2073 (w), 1995 (w), 1681 (m), 1608 (w), 1544 (vs), 1356 (m),, 1297 (m), 1174 (s), 1050 (m), 801 (s), 702 (m), 658 (m), 553 (m) cm^{-1}.

*2,13-dibutyl-3,14-dihydroxybenzo[rst]benzo[12,1]tetrapheno[7,8,9-cde]naphtho[2,1,8-klm]pentaphene-1,12-dione (**S8**)*

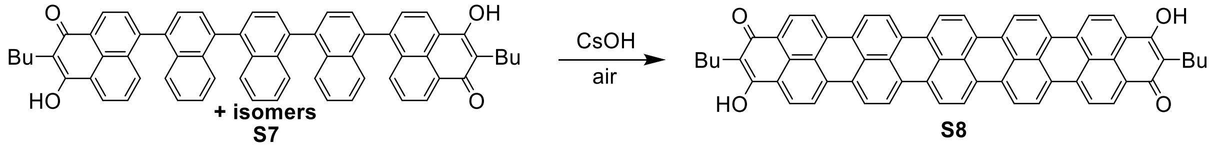

In a Ni crucible open to air, 175 mg (0.20 mmol, 1.0 eq) of **S7** (isomer mixture) and 3.5 g monohydrated CsOH were homogenized. It was heated to 285 °C for 2 h on air. In that time, a black melt was formed and gas evolution ceased. After cooling to room temperature, the melt was taken up in water (200 mL), and the black suspension was acidified with 2M acetic acid to pH = 5 and a black precipitate was formed. The precipitate was ultrasonicated for 5 min and collected

by filtration. The purple, insoluble solid was washed with water, methanol, acetone, THF and diethyl ether until the filtrate remains colorless. After drying at 120 °C overnight, 93 mg of **S8** were obtained as black solid. Due to the insolubility of **S8**, no NMR measurements could be acquired and it was directly used for the next step without further purification. IR (ATR), ṽ = 3831 (w), 3727 (w), 2356 (w), 2312 (w), 2196 (m), 2121 (w), 2042 (w), 1991 (w), 1952 (w), 1550 (m), 1186 (s), 802 (s), 749 (m), 707 (m), 644 (w) cm^{-1}.

*1,3,6,8-tetrakis((triisopropylsilyl)oxy)pyrene (**5**)*

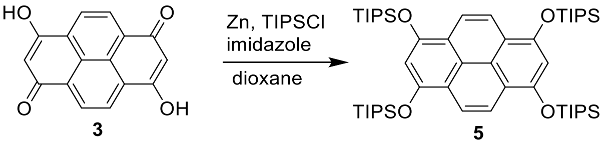

Pyrenequinone (**3**, 264 mg, 1.0 mmol, 1.0 eq), Zn dust (523 mg, 8.0 mmol, 8.0 eq), imidazole (545 mg, 8.0 mmol, 8.0 eq) and triisopropyl chloride (1.7 mL, 8.0 mmol, 8.0 eq) were heated to 90 °C for 18 h under argon atmosphere in 20 mL 1,4-dioxane. The brownish solution was cooled to room temperature, filtered and the solvent was evaporated in vacuo. The brown residue was purified by column chromatography over neutral aluminum oxide (eluent: DCM), the solvent was removed in vacuo and the residual yellow oil was dissolved in 30 mL *n*-pentane and precipitated at -80 °C. 78 mg (0.08 mmol, 8%) of the off-white solid **5** were obtained after removing the supernatant and drying in vacuo. ^{1}H NMR (500.1 MHz, $CDCl_3$): δ_H = 1.19 (d, $^{3}J_{H,H}$ = 7.5 Hz, 72H, $(CH_3)_2CH$), 1.44 (hept, $^{3}J_{H,H}$ = 7.5 Hz, 12H, $(CH_3)_2CH$), 7.02 (s, 2H, *H*a), 8.06 (s, 4H, *H*b) ppm. ^{13}C NMR (125.8 MHz, $CDCl_3$): δ_C = 13.5, 18.3, 107.5, 118.4, 118.6, 127.9, 149.5 ppm. HR-MS (LIFDI+) m/z clcd. for $[C_{52}H_{90}O_4Si_4]^+$: 890.59161 (found: 890.59229). IR (ATR), ṽ = 2945 (m), 2866 (m), 2043 (w), 1950 (w), 1590 (m9, 1500 (m), 1464 (m), 1388 (w), 1309 (vs), 1193 (m), 1153 (m), 928 (w), 889 (s), 689 (m), 621 (w9, 497 (w) cm^{-1}. λ_{max} (abs.) = 380 nm (DCM, ε = 24405 $L\cdot mol^{-1}\cdot cm^{-1}$). λ_{max} (em.) = 419 nm (DCM). CV: $E_{1/2}(Ox_1)$ = 0.26 V (vs. Fc/Fc^+), $E_{1/2}(Ox_2)$ = 0.77 V (vs. Fc/Fc^+).

*1,3,8,10-tetrakis((trimethylsilyl)oxydibenzo[cd,lm]-perylene (**4**)*

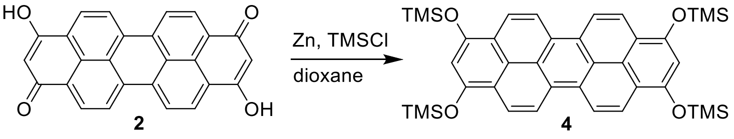

Peropyrenequinone (**2**, 778 mg, 2.0 mmol, 1.0 eq), Zn dust (1.05 g, 16.0 mmol, 8.0 eq) and trimethylsilyl chloride (2.0 mL, 16.0 mmol, 8.0 eq) were stirred for 3 h under argon atmosphere at 100 °C in 40 mL 1,4-dioxane. The greenish solution was cooled to room temperature and all volatile components were removed in vacuo. The residue was taken up in 40 mL of dichloromethane and filtered. After removing the solvent under vacuum, the crude product was washed with *n*-pentane. 647 mg (0.95 mmol, 48%) of **4** were obtained as an orange solid. X-ray suited single crystals of **4** were grown by slow gas phase diffusion of *n*-pentane into a saturated DCM solution of **4** at -18 °C. ^{1}H NMR (500.1 MHz, $CDCl_3$): δ_H = 0.46 (s, 36H, $Si(CH_3)_3$), 7.17 (s, 2H, *H*a), 8.46 (d, $^{3}J_{H,H}$ = 9.4 Hz, 4H, *H*b), 8.98 (d, $^{3}J_{H,H}$ = 9.6 Hz, 4H, *H*c) ppm. ^{13}C NMR (125.8 MHz, $CDCl_3$): δ_C = 0.7, 109.0, 118.7, 120.2, 121.5, 124.0, 125.5, 128.0, 150.1 ppm. HR-MS (EI+) m/z clcd. for $[C_{36}H_{46}O_4Si_4]^+$: 678.24409 (found: 678.24731). IR (ATR), ṽ = 3072 (m), 2954 (w), 2864 (w), 1624 (w), 1596 (w), 1554 (m), 1504 (m), 1482 (w), 1423 (w),1393 (w), 1352 (w), 1292 (w), 1251 (m), 1190 (m), 1167 (s), 1147 (w), 1089 (w), 915 (w), 873 (m), 838 (m), 785 (w), 749 (w), 681 (w), 645 (w), 559 (w) cm^{-1}. λ_{max} (abs.) = 479 nm (DCM, ε = 42896 $L\cdot mol^{-1}\cdot cm^{-1}$). λ_{max} (em.) = 495 nm (DCM). CV: $E_{1/2}(Ox_1)$ = -0.09 V (vs. Fc/Fc^+), $E_{1/2}(Ox_2)$ = 0.17 V (vs. Fc/Fc^+).

*1,3,10,12-tetrakis((trimethylsilyl)oxy)benzo[rst]dinaphtho[8,1,2-cde:2',1',8'-klm]pentaphene (**13**)*

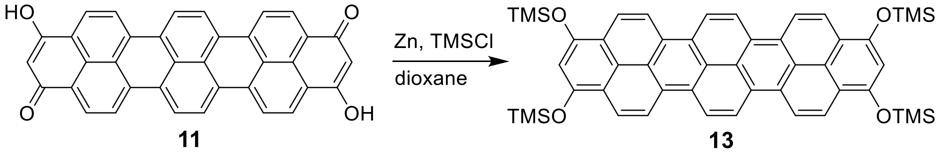

Teropyrenequinone **11** (153 mg, 0.3 mmol, 1.0 eq), Zn dust (315 mg, 4.8 mmol, 16.0 eq) and trimethylsilyl chloride (0.6 mL, 4.8 mmol, 16.0 eq) were stirred for 2 h under argon atmosphere at 100 °C in 40 mL 1,4-dioxane. The red solution was cooled to

room temperature and all volatile components were removed in vacuo. The residue was taken up in 20 mL of dichloromethane and filtered. After removing the solvent under vacuum, the crude product was washed with *n*-pentane. 45 mg (0.06 mmol, 10% over 2 steps) of **13** were obtained as an orange solid. X-ray suited single crystals of **13** were grown by slow gas phase diffusion of *n*-pentane into a saturated DCM solution of **13** at room temperature. ^{1}H NMR (500.1 MHz, $CDCl_3$): δ_H = 0.52 (s, 36H, Si(C*H*$_3$)$_3$), 7.24 (s, 2H, *H*a), 8.59 (d, $^{3}J_{H,H}$ = 9.3 Hz, 4H, *H*b), 9.17 (d, $^{3}J_{H,H}$ = 9.5 Hz, 4H, *H*c), 9.42 (s, 4H, *H*d) ppm. ^{13}C NMR (125.8 MHz, $CDCl_3$): δ_C = 0.8, 109.0, 118.7, 120.2, 122.2, 122.6, 123.7, 124.4, 124.9, 126.5, 127.8, 150.6 ppm. HR-MS (LIFDI+) m/z clcd. for $[C_{48}H_{50}O_4Si_4]^{+}$: 802.27861 (found: 802.27765). IR (ATR), ṽ = 2958 (w), 1592 (m), 1499 (m), 1391 (m), 1350 (w), 1301 (s), 1257 (w), 1188 (w), 1149 (w), 1021 (w), 878 (s), 848 (vs), 784 (m), 756 (w), 409 (w) cm^{-1}. λ_{max} (abs.) = 563 nm (DCM, ε = 77431 L·mol^{-1}·cm^{-1}). λ_{max} (em.) = 577 nm (DCM). CV: $E_{1/2}(Ox_1)$ = -0.04 V (vs. Fc/Fc$^+$), $E_{1/2}(Ox_2)$ = 0.25 V (vs. Fc/Fc$^+$).

*1,3,10,12-tetrakis((triisopropylsilyl)oxy)benzo[rst]dinaphtho[8,1,2-cde:2',1',8'-klm]pentaphene (**14**)*

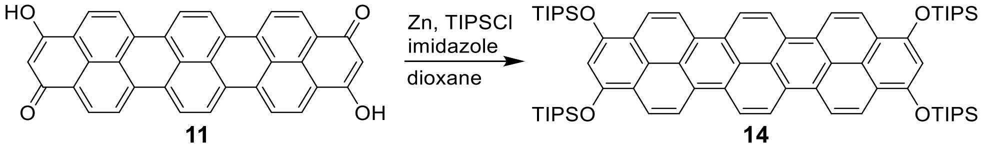

11 14

Teropyrenequinone **11** (180 mg, 0.35 mmol, 1.0 eq), Zn dust (190 mg, 2.91 mmol, 8.3 eq), imidazole (198 mg, 2.91 mmol, 8.3 eq) and triisopropyl chloride (0.6 mL, 2.9 mmol, 8.3 eq) were heated to 95 °C for 18 h under argon atmosphere in 20 mL 1,4-dioxane. The red solution was cooled to room temperature, diluted with 20 mL DCM and filtered over a pad of neutral aluminum oxide and rinsed with additional 50 mL DCM. The solvent was removed in vacuo and the purple residue was washed with diethyl ether (20 mL) and *n*-pentane (20 mL). 62 mg (0.05 mmol, 8% over 2 steps) of the purple-red solid **14** were obtained after drying in vacuo. ^{1}H NMR (500.1 MHz, $CDCl_3$): δ_H = 1.27 (d, $^{3}J_{H,H}$ = 7.5 Hz, 72H, $(CH_3)_2$C*H*), 1.57 (hept, $^{3}J_{H,H}$ = 7.5 Hz, 12H, (C*H*$_3$)$_2$CH), 7.27 (s, 2H, *H*a), 8.70 (d, $^{3}J_{H,H}$ = 9.4 Hz, 4H, *H*b), 9.14 (d, $^{3}J_{H,H}$ = 9.6 Hz, 4H, *H*c), 9.35 (s, 4H, *H*d) ppm. ^{13}C NMR (125.8 MHz, $CDCl_3$): δ_C = 13.5, 18.3, 107.5, 118.0, 119.9, 122.2, 122.5, 123.8, 124.4, 124.7, 126.5, 127.9, 151.5 ppm. HR-MS (LIFDI+) m/z clcd. for $[C_{72}H_{98}O_4Si_4]^{+}$: 1138.65421 (found: 1138.65888). IR (ATR), ṽ = 2944 (w), 2894 (w), 2866 (w), 1589 (w), 1497 (m), 1463 (s), 1389 (m), 1348 (s), 1299 (s), 1259 (m), 1187 (m), 1147 (w), 1019 (m), 860 (m), 781 (s), 681 (w) cm^{-1}. λ_{max} (abs.) = 572 nm (DCM, ε = 129393 L·mol^{-1}·cm^{-1}). λ_{max} (em.) = 587 nm (DCM). CV: $E_{1/2}(Red_1)$ = -2.21 V (vs. Fc/Fc$^+$), $E_{1/2}(Ox_1)$ = 0.00 V (vs. Fc/Fc$^+$), $E_{1/2}(Ox_2)$ = 0.24 V (vs. Fc/Fc$^+$).

*((2,13-dibutylbenzo[rst]benzo[12,1]tetrapheno[7,8,9-cde]naphtho[2,1,8-klm]pentaphene-1,3,12,14-tetrayl)tetrakis(oxy))-tetrakis(trimethylsilane) (**S4**)*

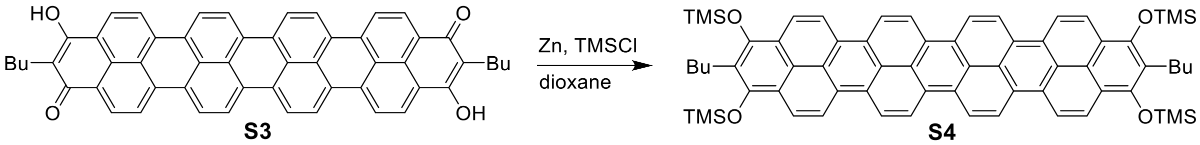

S3 S4

Quarteropyrenequinone **S3** (75 mg, 0.10 mmol, 1.0 eq), Zn dust (52 mg, 0.80 mmol, 8.0 eq) and trimethylsilyl chloride (0.2 mL, 1.6 mmol, 16.0 eq) were stirred for 3 h under argon atmosphere at 100 °C in 10 mL 1,4-dioxane. The dark blue solution was cooled to room temperature and all volatile components were removed in vacuo. The residue was taken up in 20 mL of dichloromethane and filtered. After removing the solvent under vacuum, the crude product was washed with *n*-pentane (10 mL). 22 mg (0.02 mmol, 14% over 2 steps) of **S4** were obtained as a dark blue solid. ^{1}H NMR (500.1 MHz, $CDCl_3$): δ_H = 0.48 (s, 36H, Si(C*H*$_3$)$_3$), 1.05 (t, $^{3}J_{H,H}$ = 7.5 Hz, *H*a), 1.26-1.35 (m, 8H, *H*b), 1.52-1.56 (m, 4H, *H*c), 8.58 (d, $^{3}J_{H,H}$ = 9.0 Hz, 4H, *H*d), 9.21 (d, $^{3}J_{H,H}$ = 9.5 Hz, 4H, *H*e), 9.48 (d, $^{3}J_{H,H}$ = 9.7 Hz, 4H, *H*f), 9.52 (d, $^{3}J_{H,H}$ = 9.7 Hz, 4H, *H*g) ppm. ^{13}C NMR not acquired due to low solubility. HR-MS (LIFDI+) m/z clcd. for $[C_{66}H_{70}O_4Si_4]^{+}$: 1038.43511 (found: 1038.43595). IR (ATR), ṽ = 2957 (m), 2940 (w), 1614 (s), 1500 (m), 1471 (w), 1326 (w), 1252 (m), 1221 (m), 1148 (m), 846 (vs), 784 (m), 755 (m), 658 (w) cm^{-1}. λ_{max} (abs.) = 634 nm (DCM). CV: $E_{1/2}(Red_2)$ = -2.00 V (vs. Fc/Fc$^+$), $E_{1/2}(Red_1)$ = -1.82 V (vs. Fc/Fc$^+$), $E_{1/2}(Ox_1)$ = 0.29 V (vs. Fc/Fc$^+$), $E_{1/2}(Ox_2)$ = 1.24 V (vs. Fc/Fc$^+$).

*1,3,10,12-tetrakis((triisopropylsilyl)oxy)benzo[rst]dinaphtho[8,1,2-cde:2',1',8'-klm]pentaphene (**15**)*

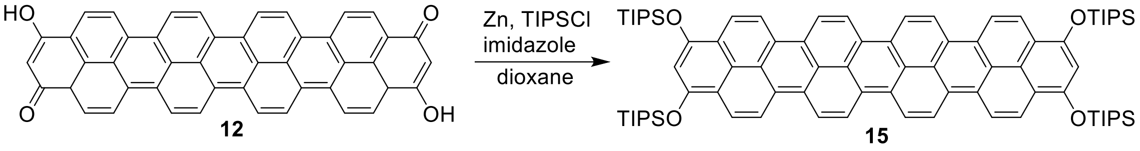

Quarteropyrenequinone **12** (189 mg, 0.30 mmol, 1.0 eq), Zn dust (320 mg, 4.80 mmol, 16.0 eq), imidazole (327 mg, 4.80 mmol, 16.0 eq) and triisopropyl chloride (1.0 mL, 4.8 mmol, 16.0 eq) were heated to 110 °C for 3 h under argon atmosphere in 20 mL 1,4-dioxane. The dark blue solution was cooled to room temperature, diluted with 20 mL chloroform and filtered over a pad of neutral aluminum oxide and rinsed with additional 50 mL DCM. The solvent was removed in vacuo and the dark blue residue was washed with methanol (20 mL), diethyl ether (20 mL) and *n*-pentane (20 mL). 68 mg (0.05 mmol, 14% over 2 steps) of the dark blue solid **15** were obtained after drying in vacuo. X-ray suited single crystals of **15** were grown by slow gas phase diffusion of *n*-pentane into a saturated DCM solution of **15** at room temperature. ^{1}H NMR (300.1 MHz, CD_2Cl_2): δ_H = 1.28 (d, $^{3}J_{H,H}$ = 7.4 Hz, 72H, $(CH_3)_2CH$), 1.56 (hept, $^{3}J_{H,H}$ = 7.5 Hz, 12H, $(CH_3)_2CH$), 7.26 (s, 2H, *H*a), 8.69 (d, $^{3}J_{H,H}$ = 9.5 Hz, 4H, *H*b), 9.10 (d, $^{3}J_{H,H}$ = 9.0 Hz, 4H, *H*c), 9.33 (br s, 8H, *H*d, *H*e) ppm. ^{13}C NMR (125.8 MHz, $CDCl_3$): δ_C = 12.4, 13.5, 17.8, 18.3, 107.5, 118.0, 119.9, 122.2, 122.3, 122.8, 123.7, 123.9, 124.4, 125.4, 126.8, 127.8, 151.6 ppm. HR-MS (LIFDI+) m/z clcd. for $[C_{82}H_{102}O_4Si_4]^+$: 1262.68551 (found: 1262.68521). IR (ATR), ṽ = 2948 (w), 2895 (w), 2866 (w), 1590 (w), 1498 (m), 1464 (s), 1389 (m), 1349 (m), 1300 (s), 1260 (m), 1186 (s), 1146 (w9, 1019 (m), 995 (w), 861 (m), 780 (vs), 681 (m) cm^{-1}. λ_{max} (abs.) = 642 nm (DCM, ε = 128215 $L{\cdot}mol^{-1}{\cdot}cm^{-1}$). λ_{max} (em.) = 670 nm (DCM). CV: $E_{1/2}(Red_2)$ = -2.19 V (vs. Fc/Fc^+), $E_{1/2}(Red_1)$ = -1.92 V (vs. Fc/Fc^+), $E_{1/2}(Ox_1)$ = -0.18 V (vs. Fc/Fc^+), $E_{1/2}(Ox_2)$ = 0.17 V (vs. Fc/Fc^+).

NMR spectra

*1,4-bis(4,4,5,5-tetramethyl-1,3,2-dioxaborolan-2-yl)naphthalene (**6**)*

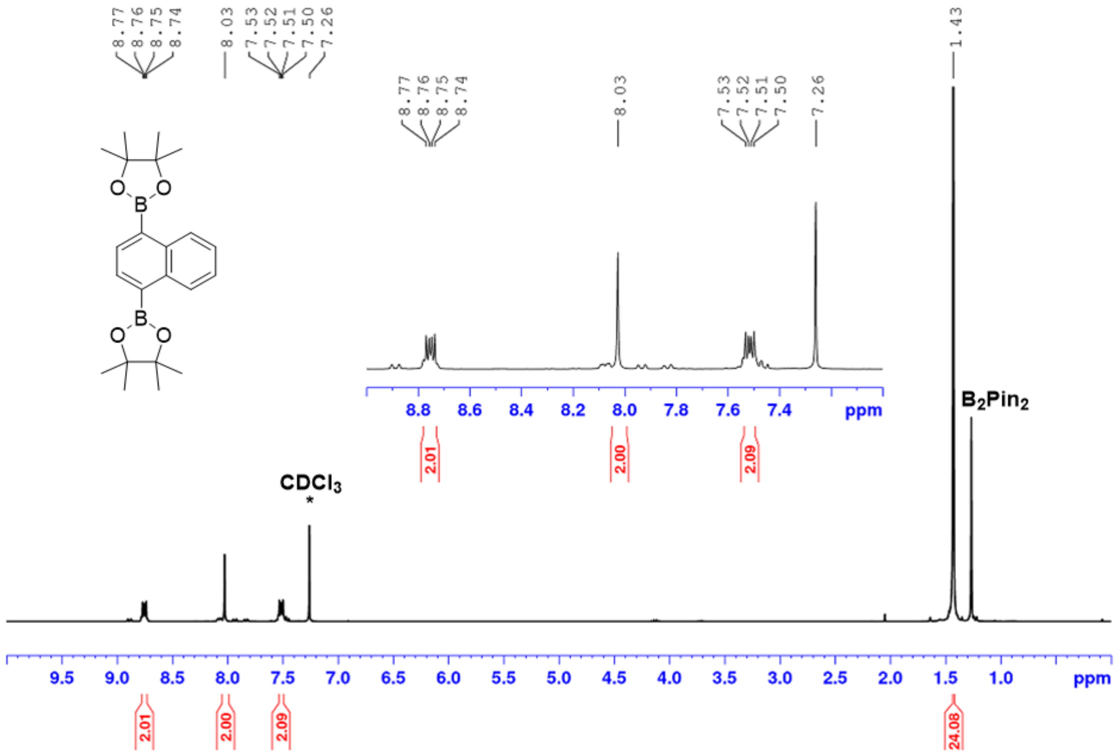

Figure S19. ^{1}H-NMR (300.1 MHz, 298K, $CDCl_3$*) of **6**, contains trace amounts (ca. 5%) of single borylated species.

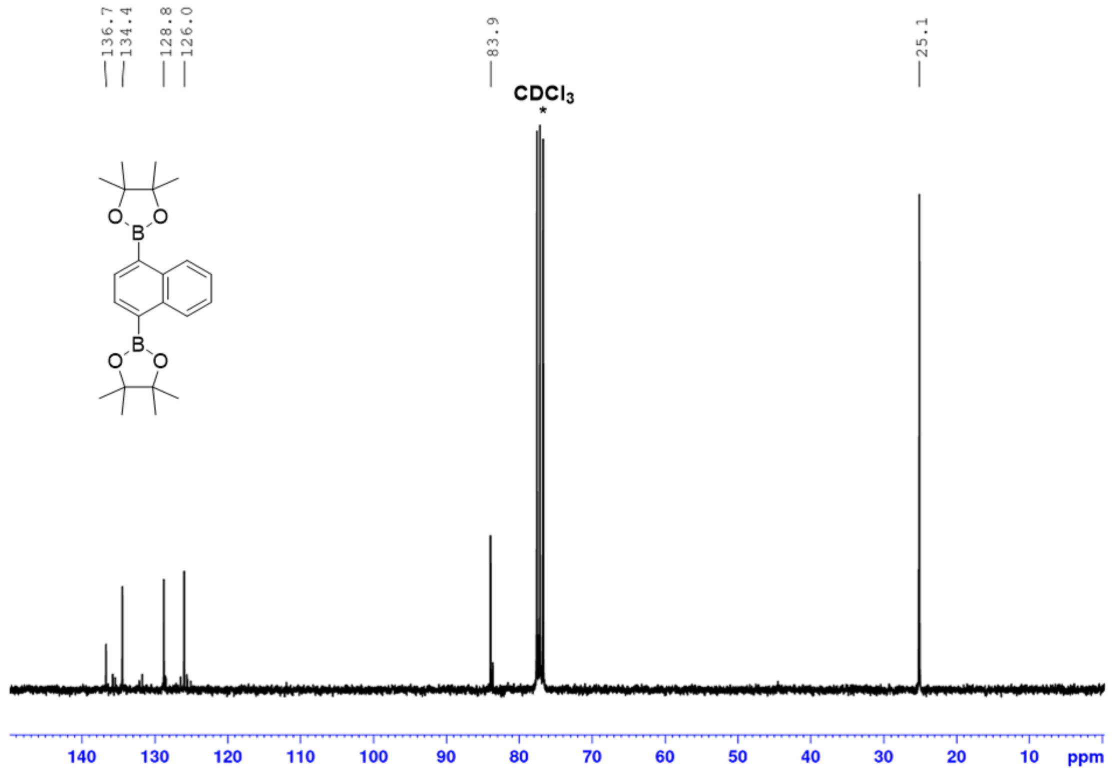

Figure S20. ^{13}C-NMR (75.5 MHz, 298K, $CDCl_3$*) of **6**, contains trace amounts (ca. 5%) of single borylated species.

*4,4’-dibromo-1,1’-binaphthyl (**S10**)*

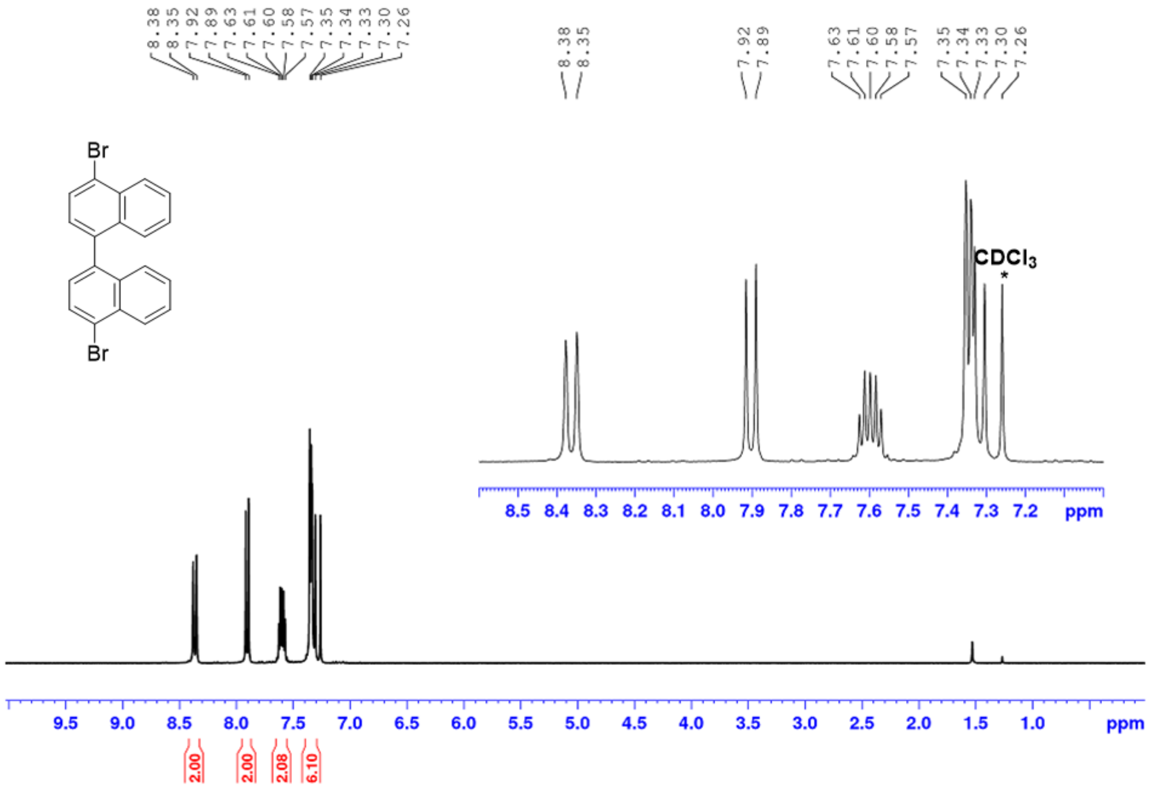

Figure S21. ^{1}H-NMR (300.1 MHz, 298K, $CDCl_3$*) of **S10**.

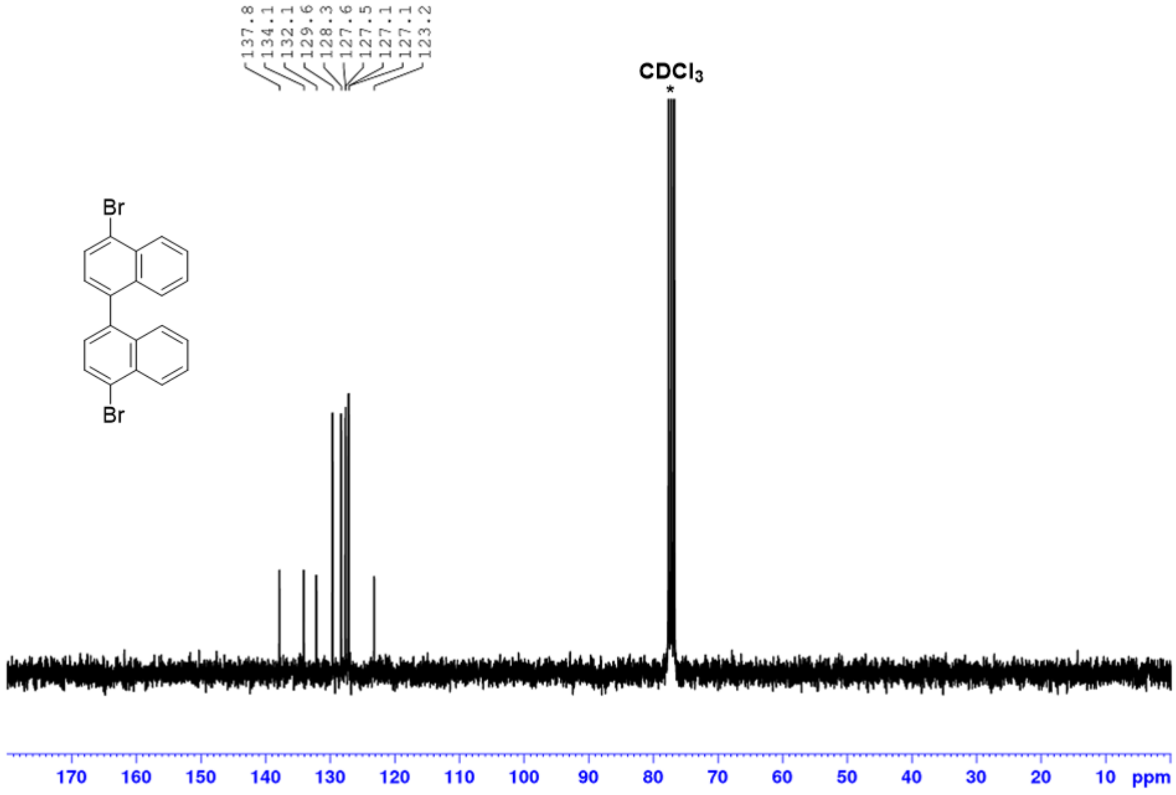

Figure S22. ^{13}C-NMR (75.5 MHz, 298K, $CDCl_3$*) of **S10**.

*4,4'-bis(4,4,5,5-tetramethyl-1,3,2-dioxaborolan-2-yl)-1,1'-binaphthalene (**7**)*

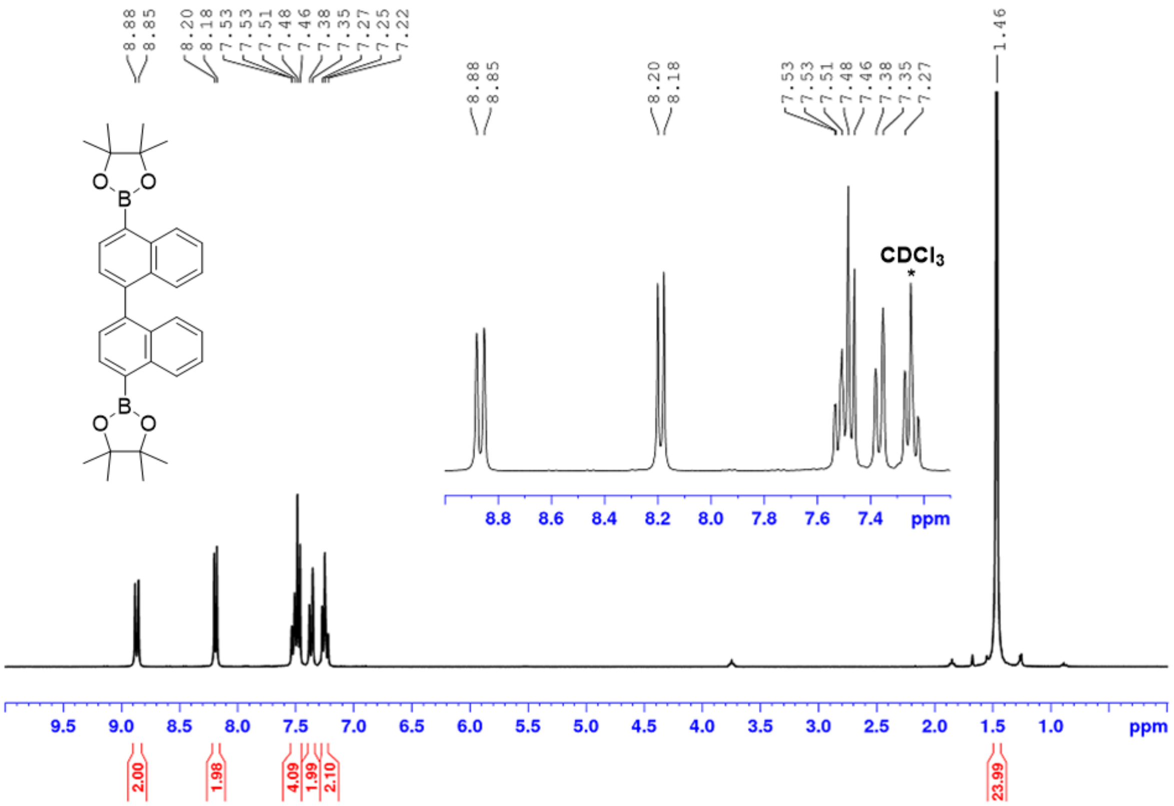

Figure S23. ^{1}H-NMR (300.1 MHz, 298K, $CDCl_3$*) of **7**.

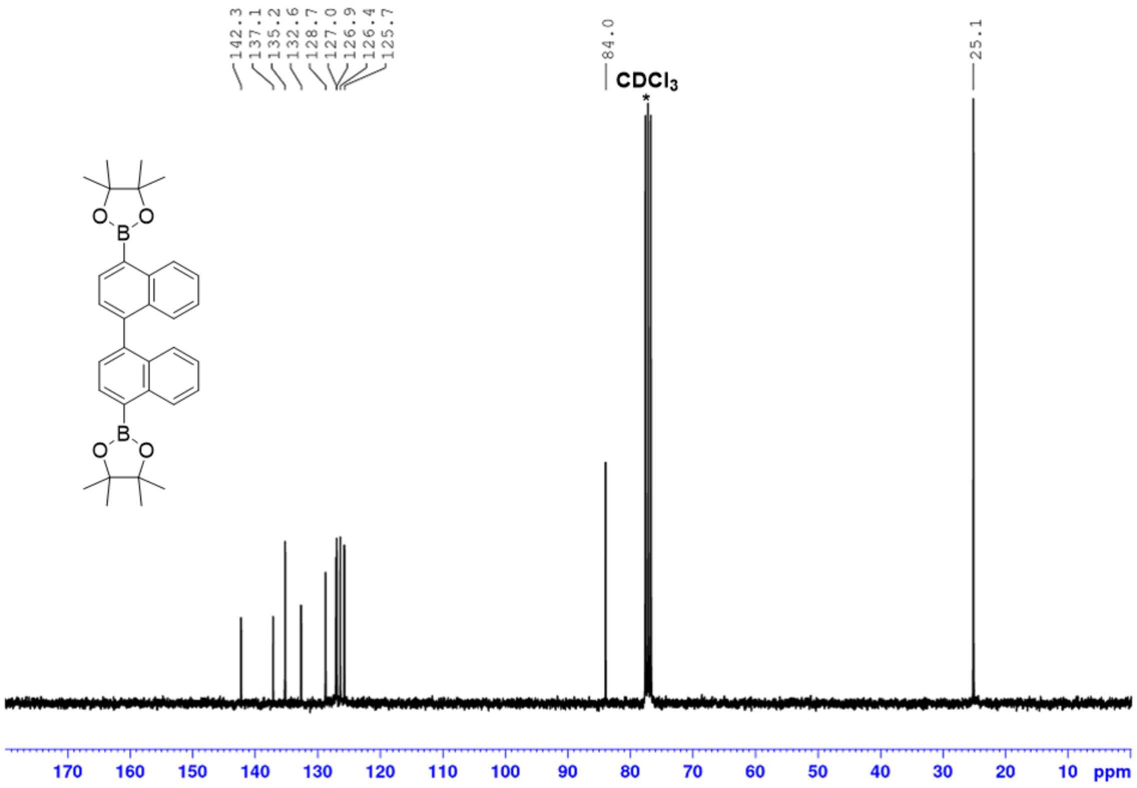

Figure S24. ^{13}C-NMR (75.5 MHz, 298K, $CDCl_3$*) of **7**.

*4,4''-dibromo-1,1':4',1''-ternaphthalene (**S5**)*

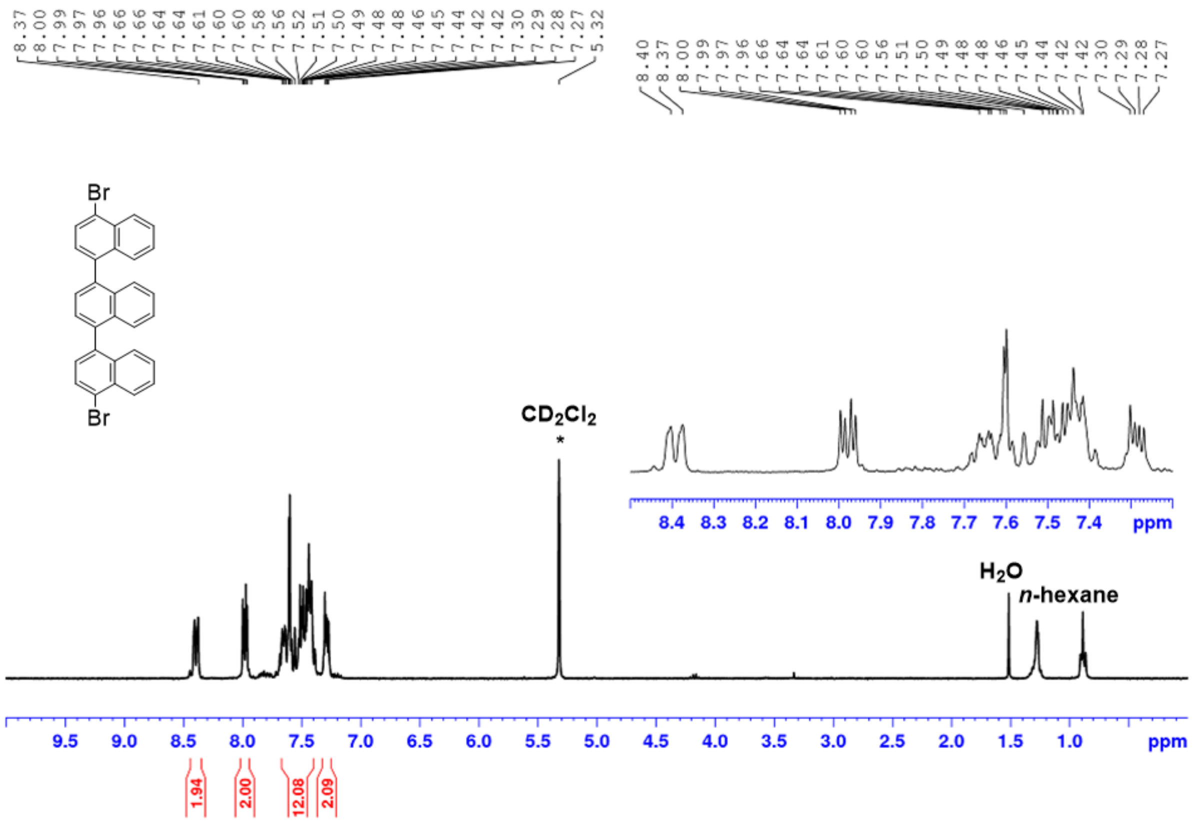

Figure S25. ^{1}H-NMR (300.1 MHz, 298K, CD_2Cl_2*) of **S5**.

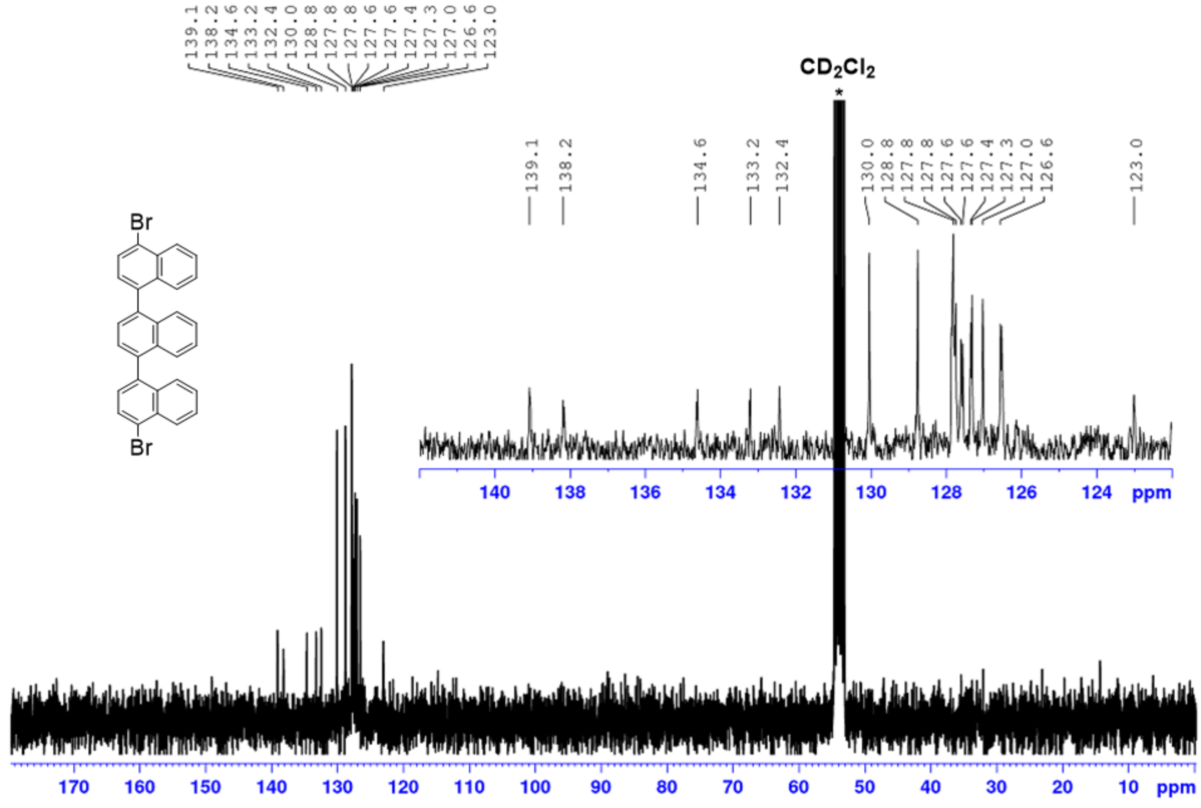

Figure S26. ^{13}C-NMR (75.5 MHz, 298K, $CDCl_3$*) of **S5**.

*4,4''-bis(4,4,5,5-tetramethyl-1,3,2-dioxaborolan-2-yl)-1,1':4',1''-ternaphthalene (**S6**)*

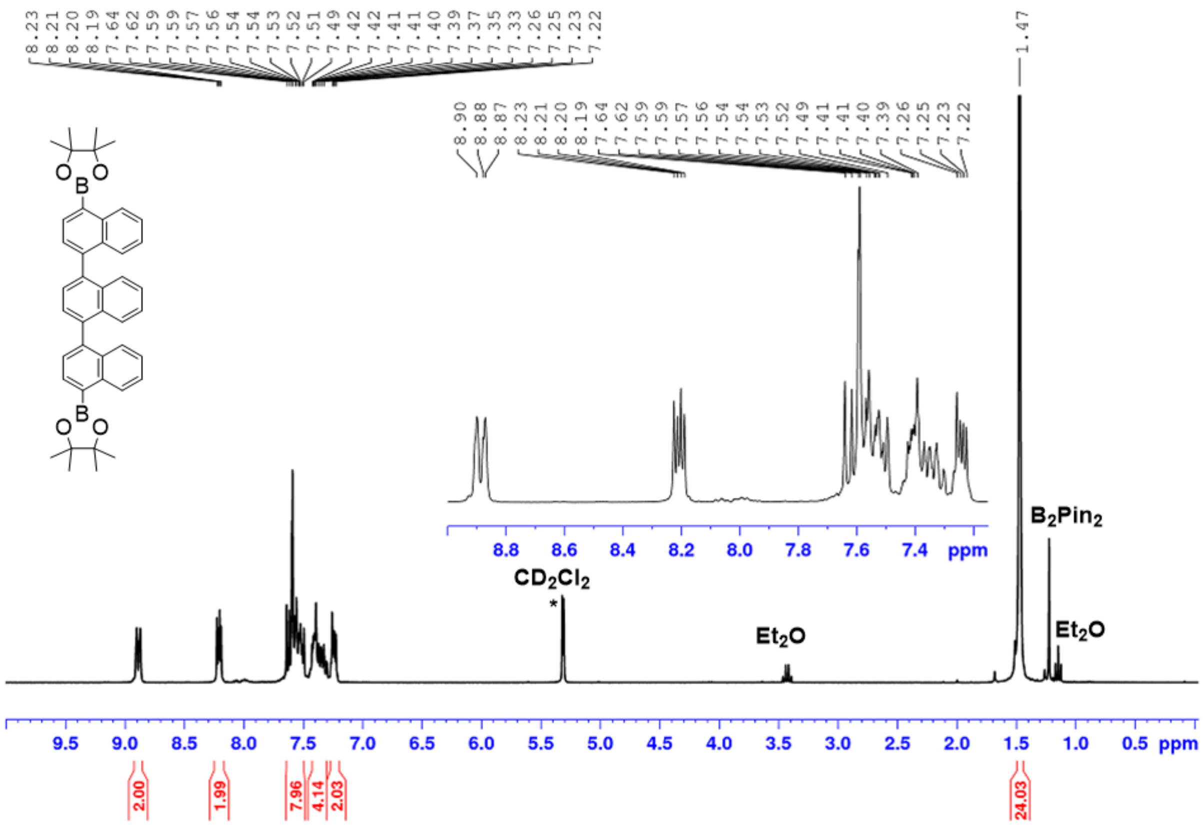

Figure S27. ^{1}H-NMR (300.1 MHz, 298K, CD_2Cl_2*) of **S6**.

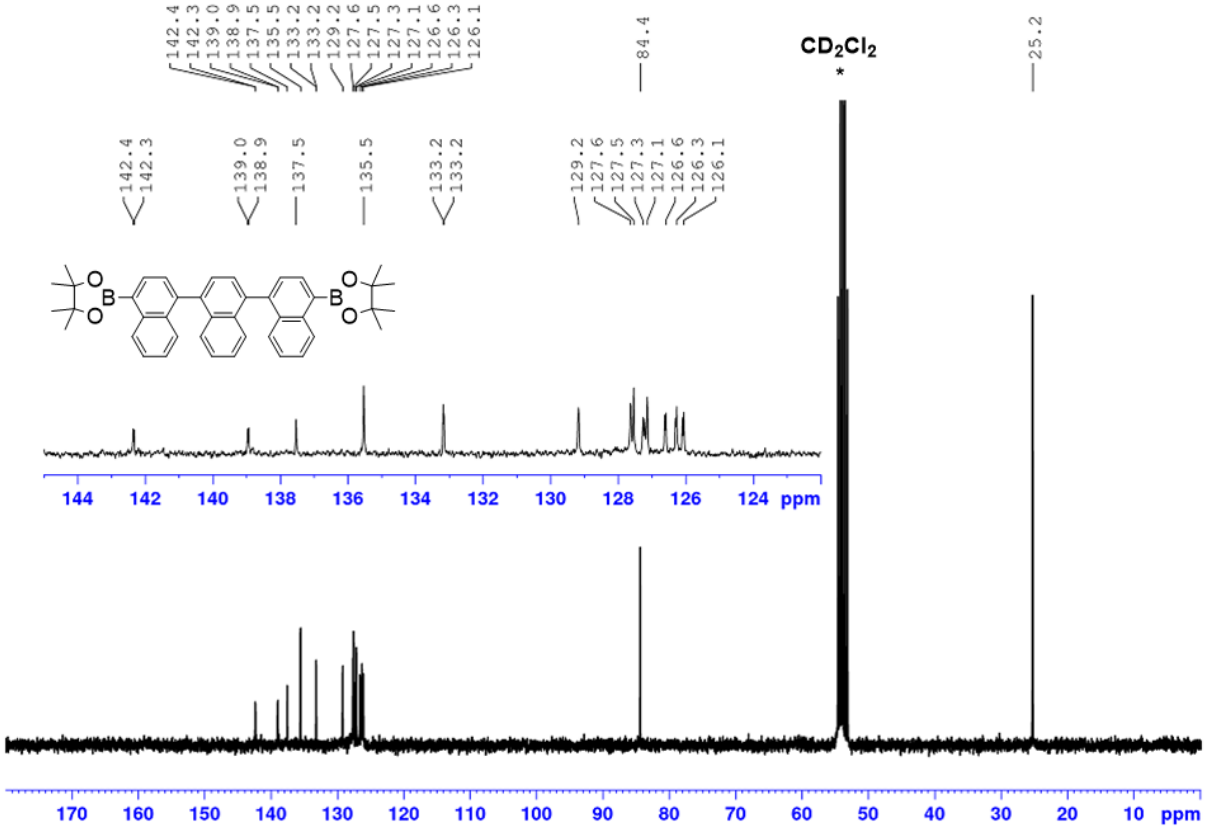

Figure S28. ^{13}C-NMR (75.5 MHz, 298K, $CDCl_3$*) of **S6**.

*6-bromo-3-hydroxy-1H-phenalen-1-one (**8**)*

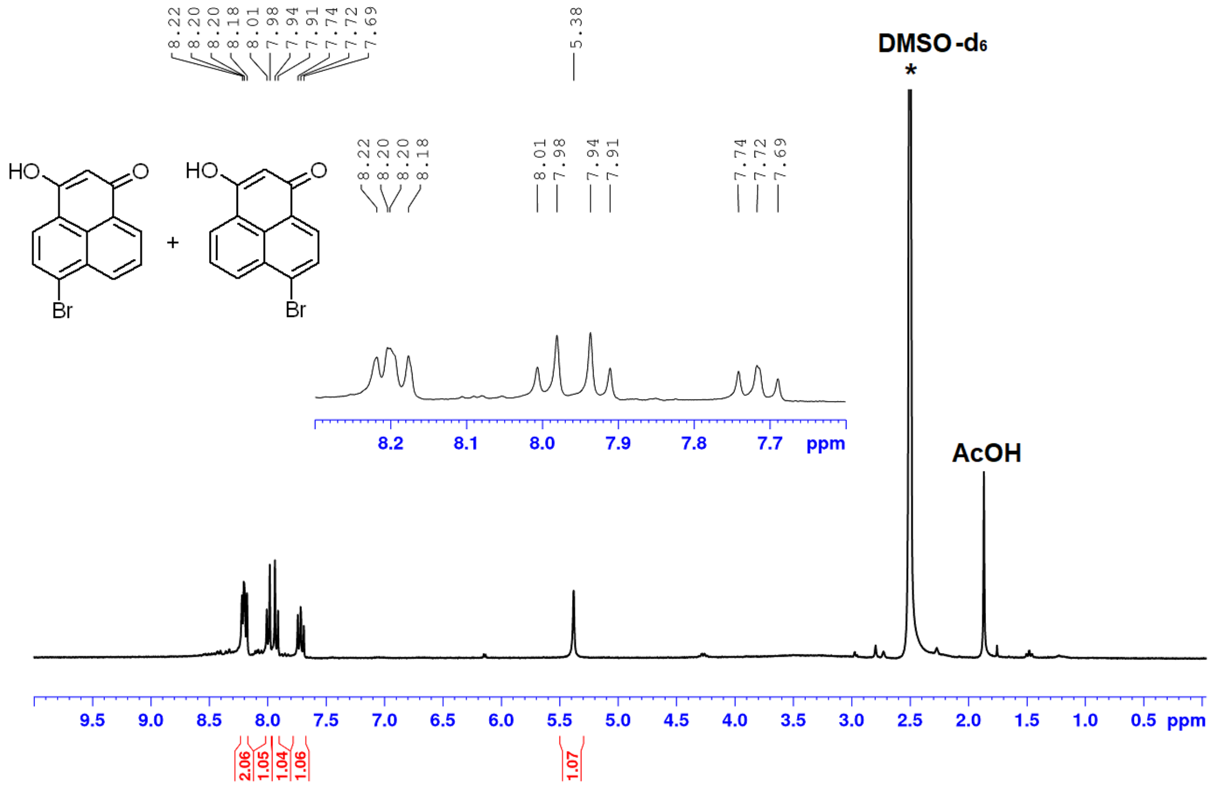

Figure S29. ^{1}H-NMR (300.1 MHz, 298K, DMSO-d_6*) of **8** (isomer mixture). Contains traces of acetic acid.

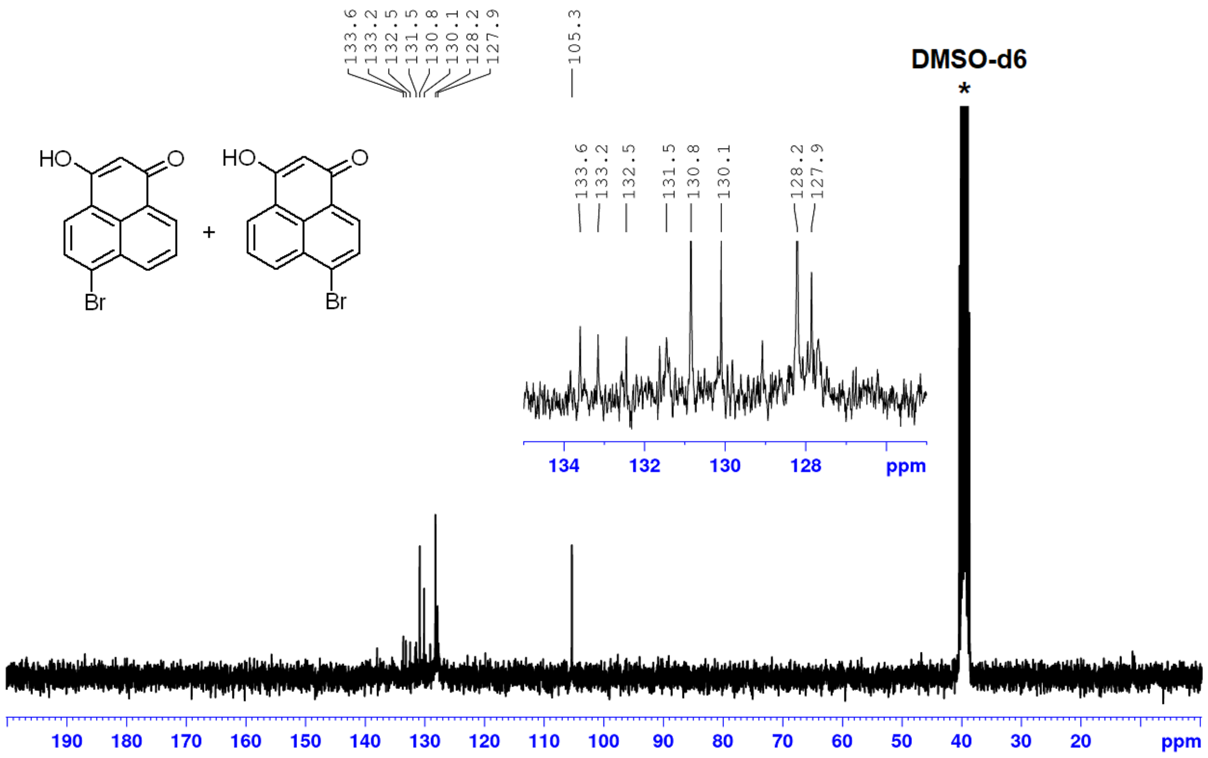

Figure S30. ^{13}C-NMR (75.5 MHz, 298K, DMSO-d_6*) of **8** (isomer mixture).

*6-bromo-2-butyl-3-hydroxy-1H-phenalen-1-one (**S1**)*

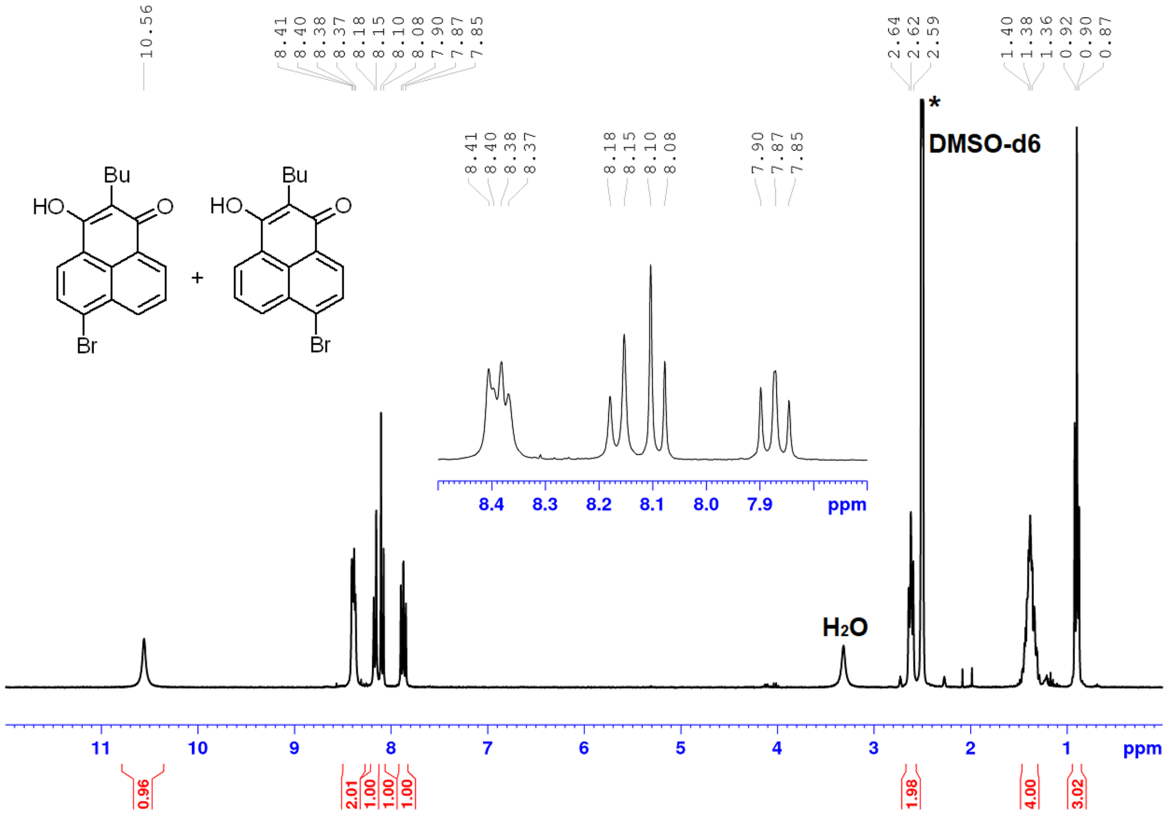

Figure S31. ^{1}H-NMR (300.1 MHz, 298K, DMSO-d$_6$*) of **S1** (isomer mixture).

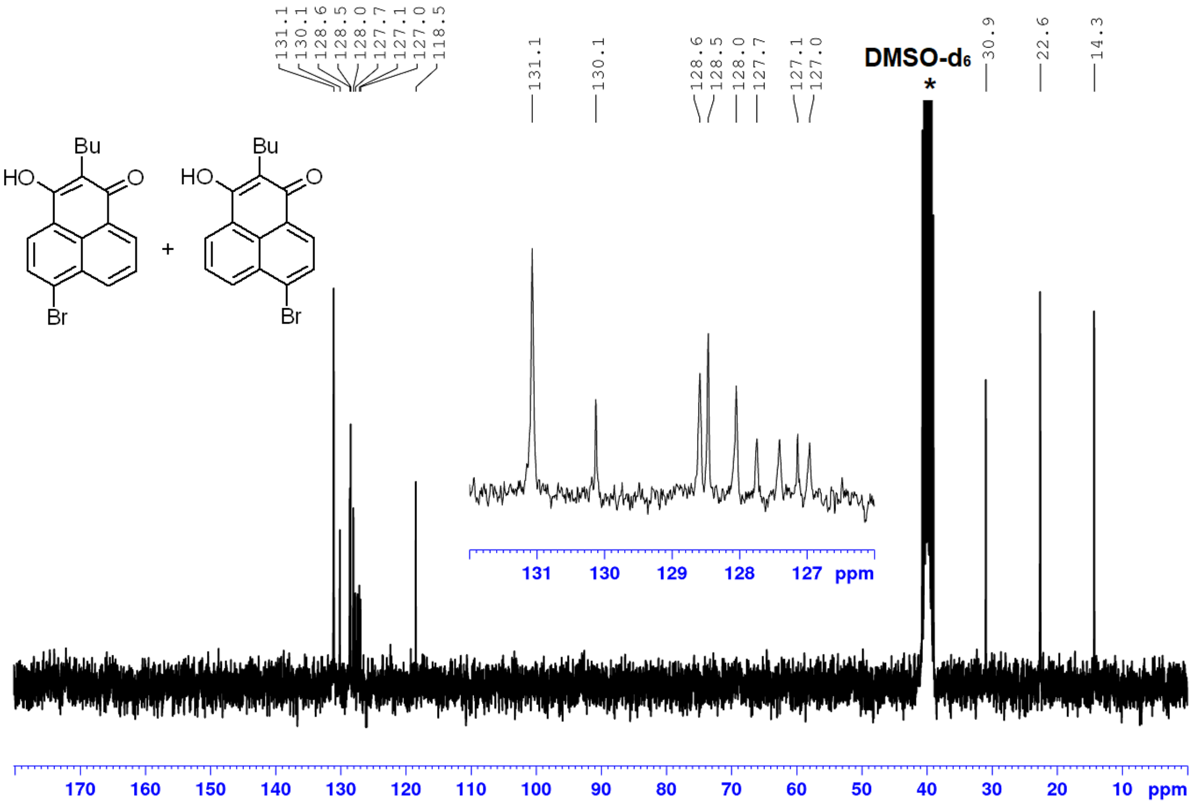

Figure S32. ^{13}C-NMR (75.5 MHz, 298K, DMSO-d$_6$*) of **S1** (isomer mixture).

*3-hydroxy-7-(4-(3-hydroxy-1-oxo-1H-phenalen-6-yl)naphthalen-1-yl)-1H-phenalen-1-one (**9**)*

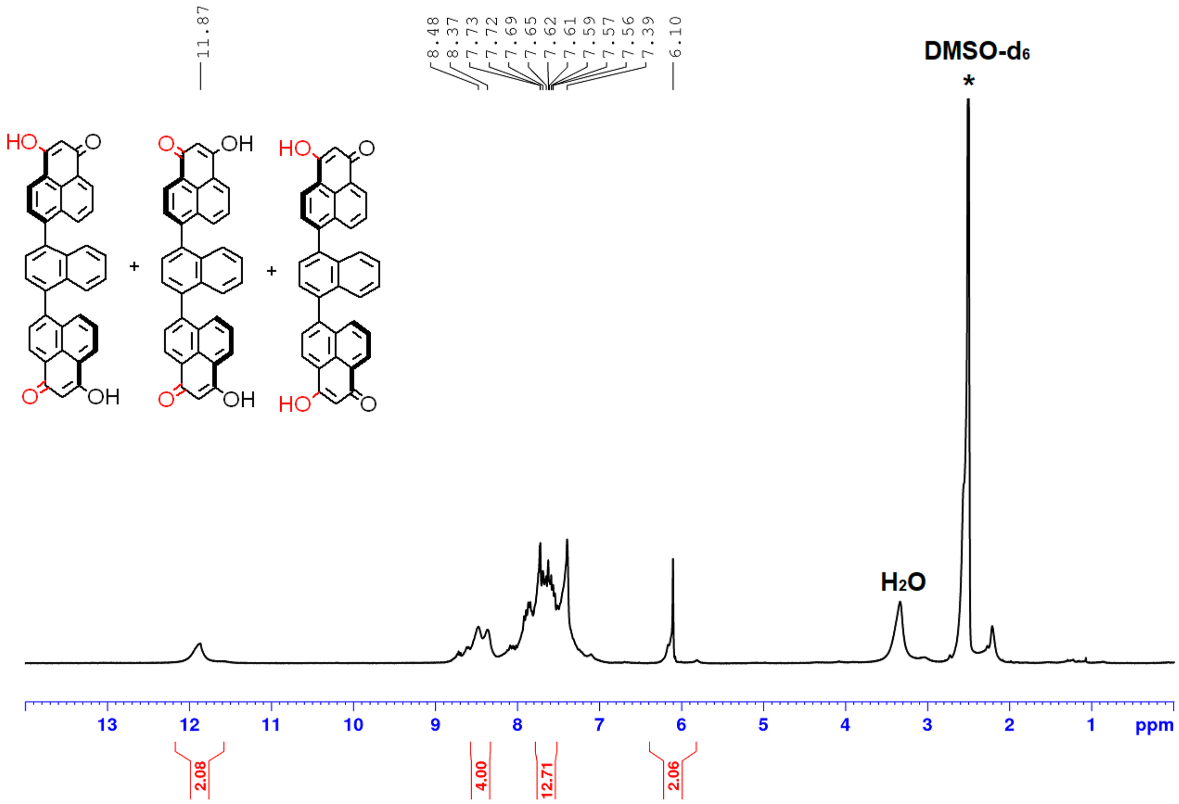

Figure S33. ^{1}H-NMR (300.1 MHz, 298K, DMSO-d$_6$*) of **9** (isomer mixture). Contains inseparable aromatic by-products.

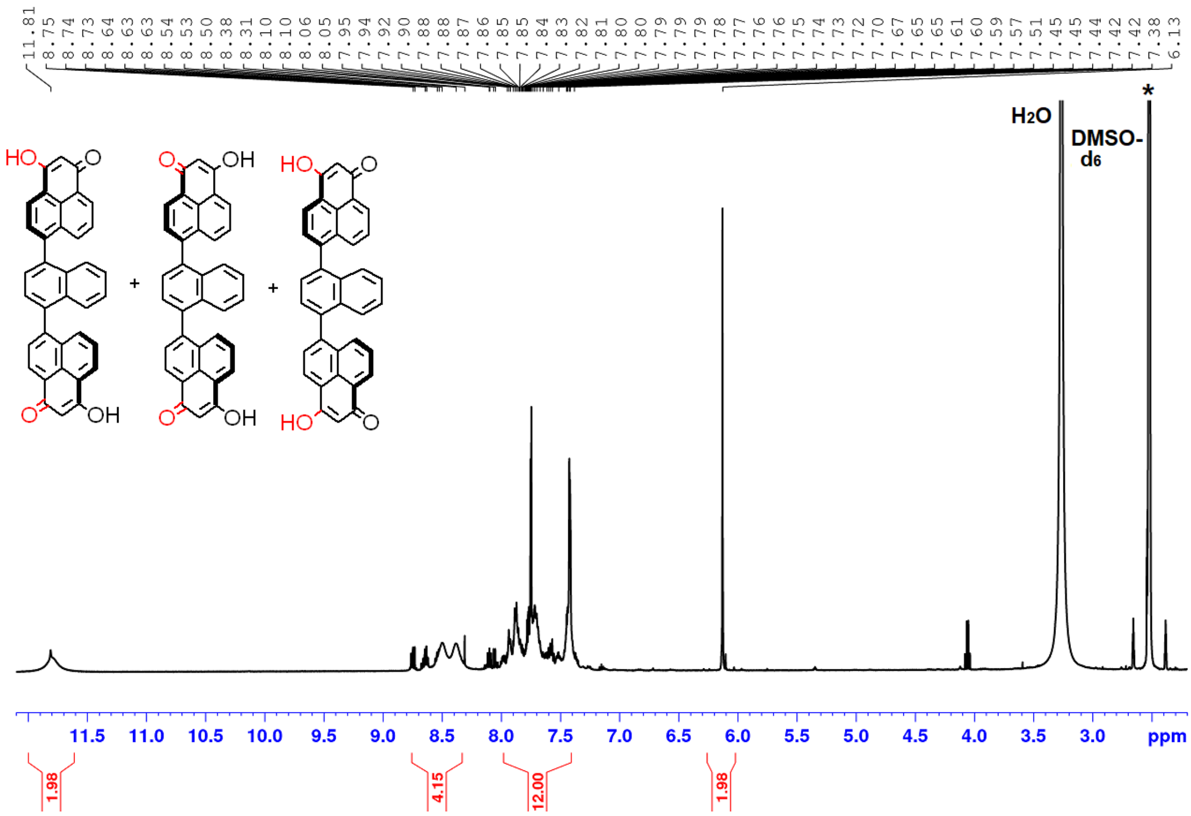

Figure S34. HT ^{1}H-NMR (500.1 MHz, 323K, DMSO-d$_6$*) of **9** (isomer mixture). Contains inseparable aromatic by-products.

*3-hydroxy-7-(4'-(3-hydroxy-1-oxo-1H-phenalen-6-yl)-[1,1'-binaphthalen]-4-yl)-1H-phenalen-1-one (**10**)*

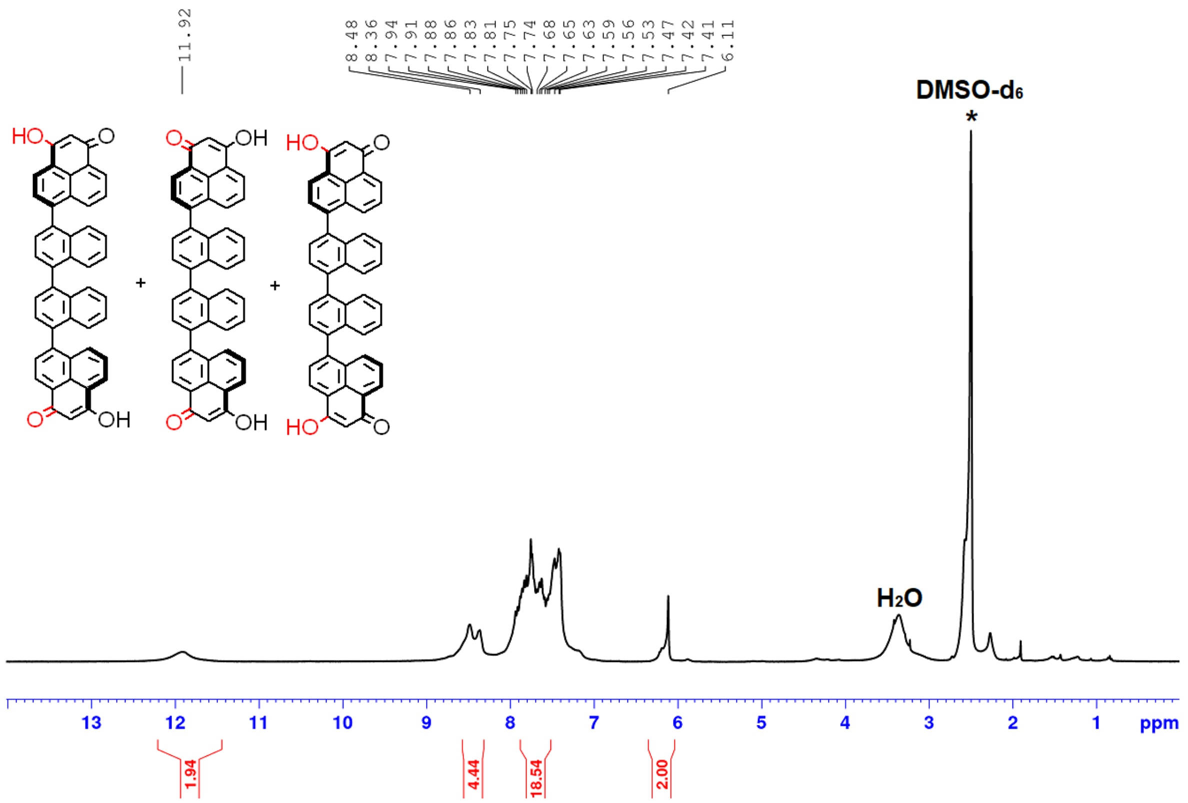

Figure S35. ^{1}H-NMR (300.1 MHz, 298K, DMSO-d_6*) of **10** (isomer mixture). Contains inseparable aromatic by-products.

*2-butyl-7-(4'-(2-butyl-3-hydroxy-1-oxo-1H-phenalen-6-yl)-[1,1'-binaphthalen]-4-yl)-3-hydroxy-1H-phenalen-1-one (**S2**)*

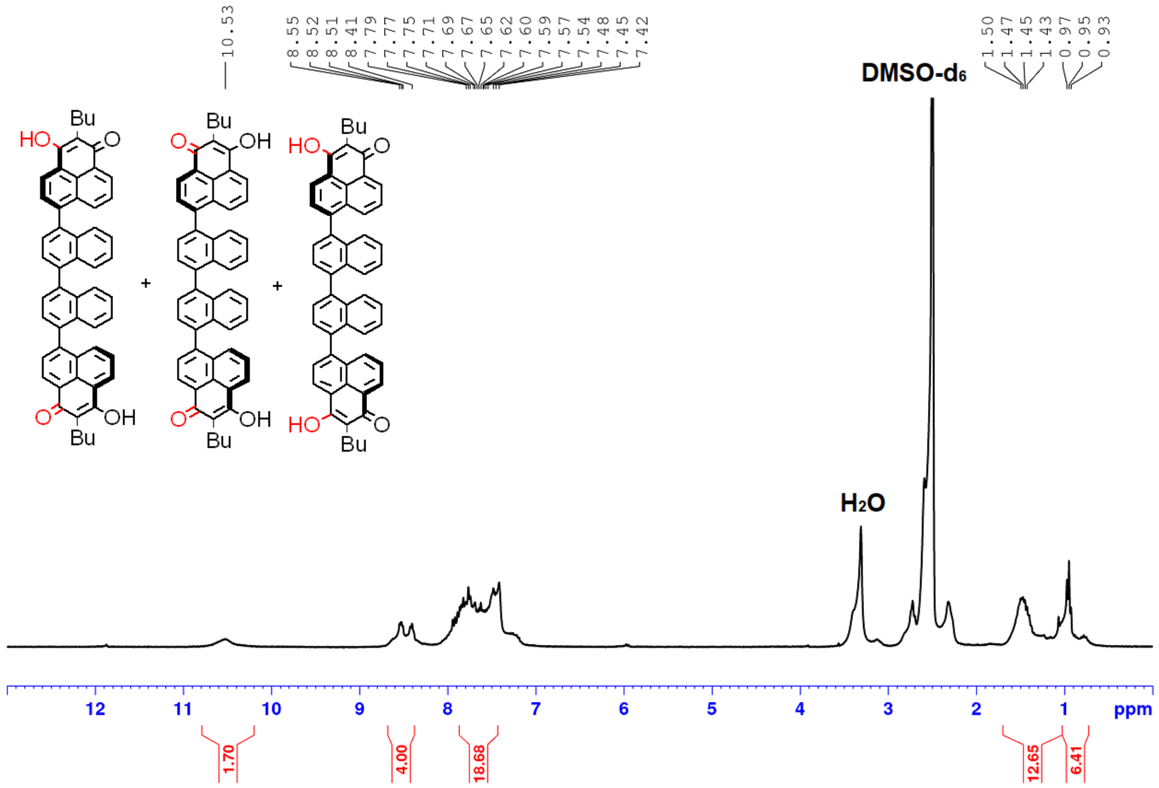

Figure S36. ^{1}H-NMR (300.1 MHz, 298K, DMSO-d_6*) of **S2** (isomer mixture). Contains inseparable aromatic by-products.

*2-butyl-7-(4'-(2-butyl-3-hydroxy-1-oxo-1H-phenalen-6-yl)-[1,1'-binaphthalen]-4-yl)-3-hydroxy-1H-phenalen-1-one (**S7**)*

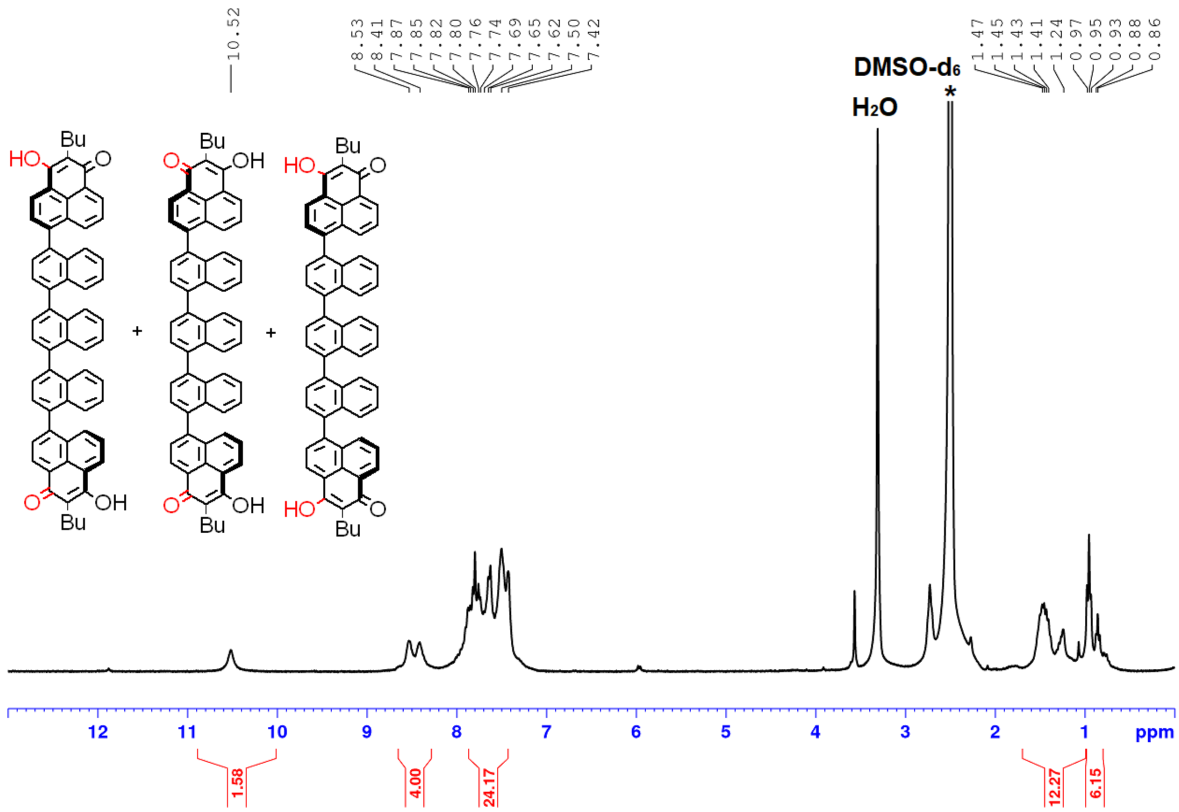

Figure S37. ^{1}H-NMR (300.1 MHz, 298K, DMSO-d$_6$*) of **S7** (isomer mixture). Contains inseparable aromatic by-products.

*1,3,6,8-tetrakis((triisopropylsilyl)oxy)pyrene (**5**)*

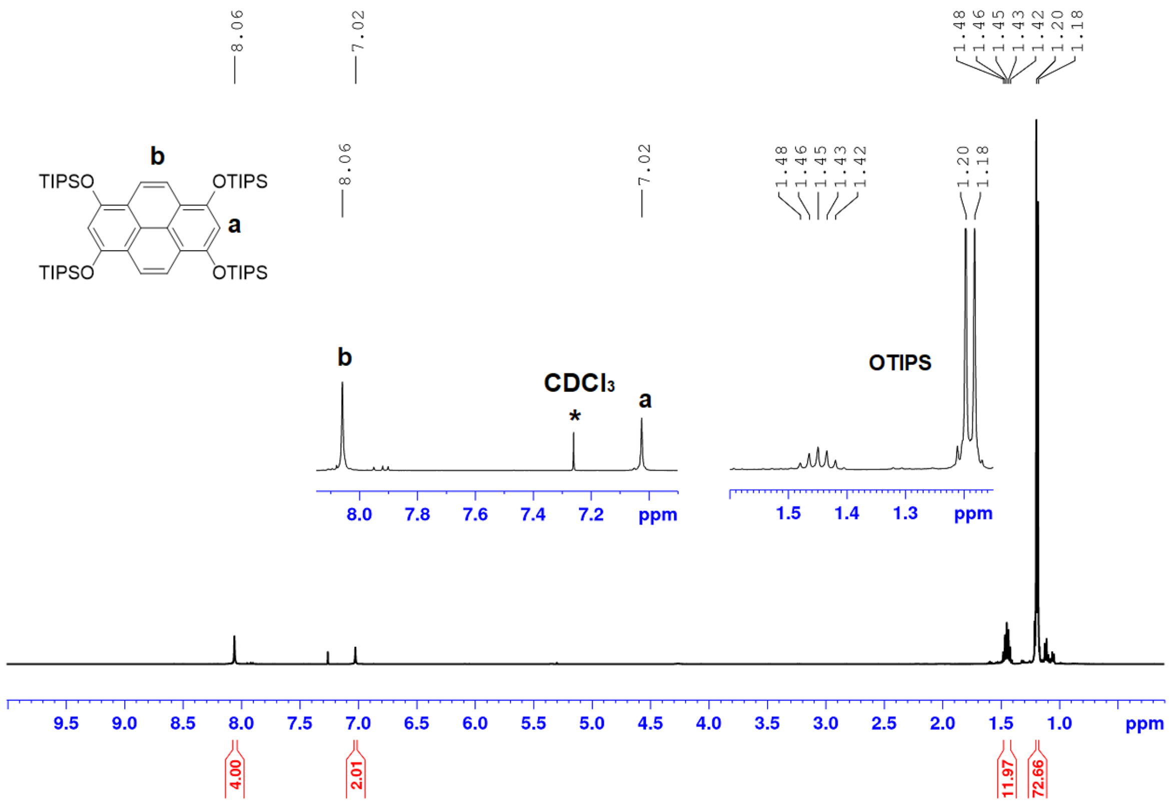

Figure S38. ^{1}H-NMR (500.1 MHz, 298K, CDCl$_3$*) of **5**.

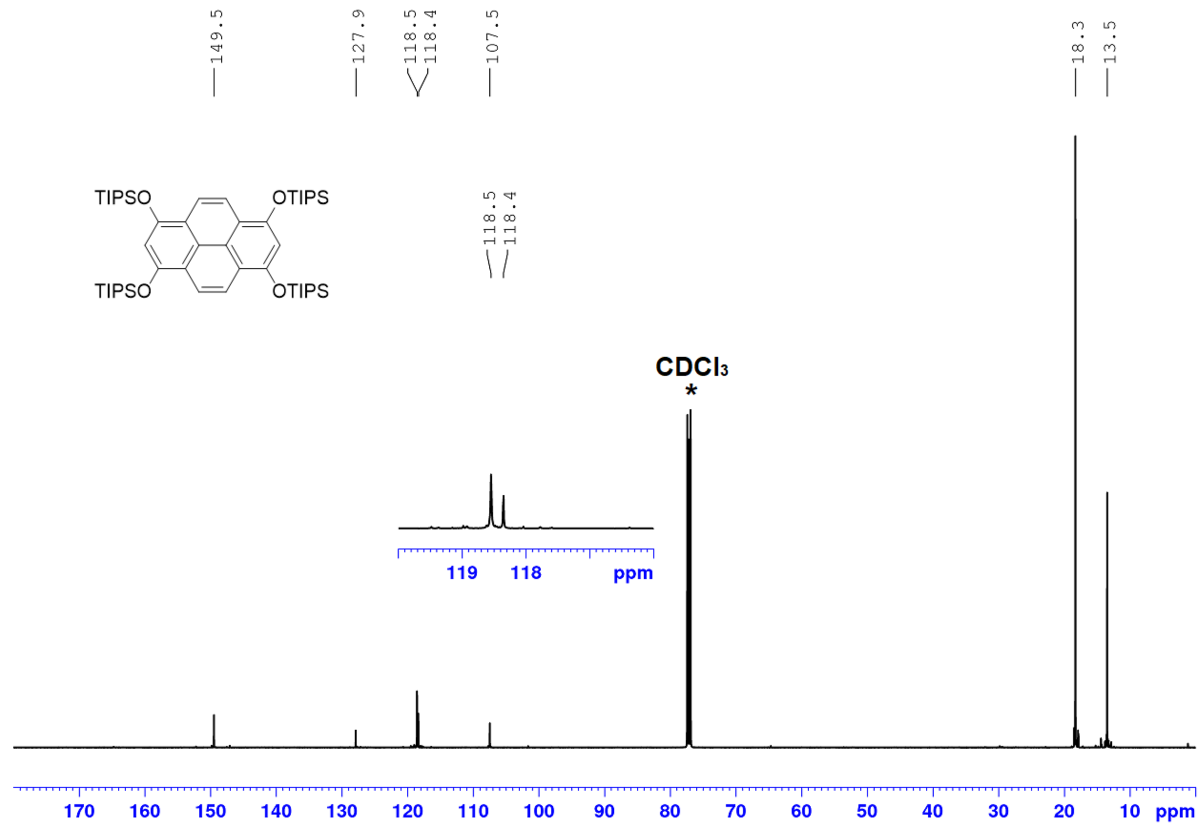

Figure S39. ^{13}C-NMR (125.8 MHz, 298K, $CDCl_3$*) of **5**.

*1,3,8,10-tetrakis((trimethylsilyl)oxydibenzo[cd,lm]-perylene (**4**)*

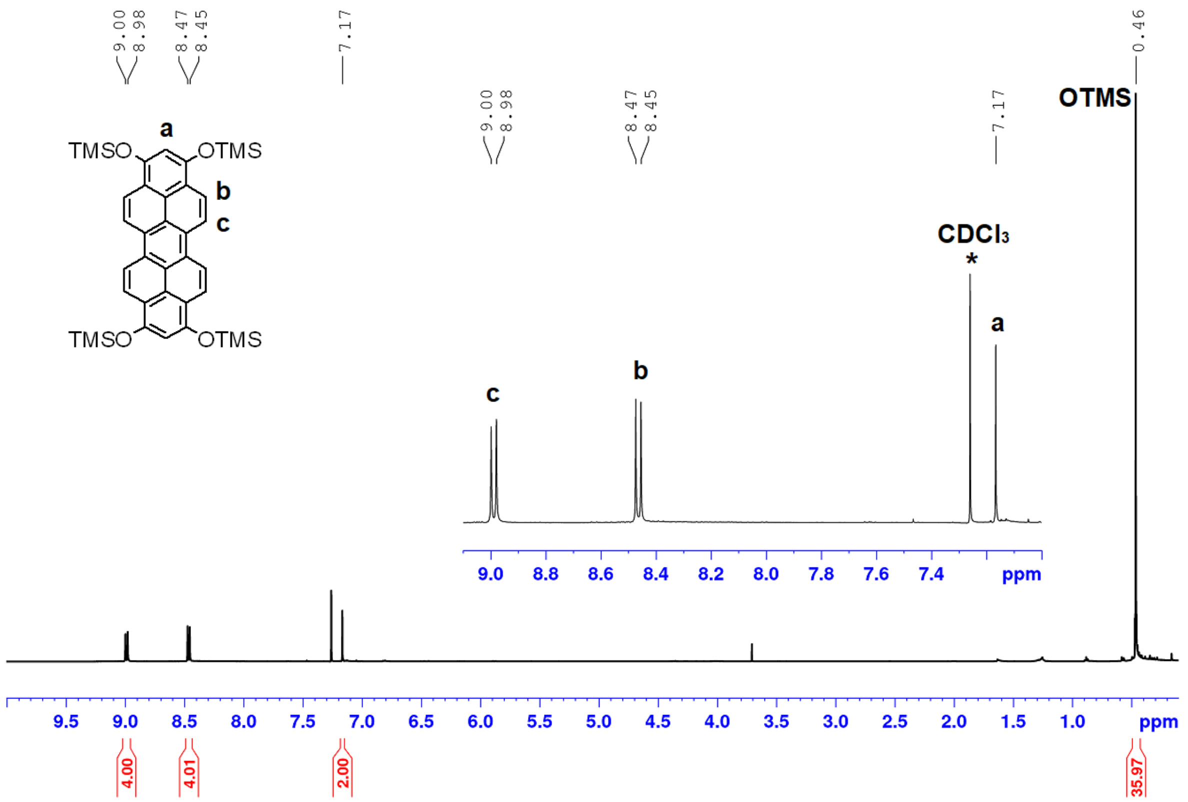

Figure S40. ^{1}H-NMR (500.1 MHz, 298K, $CDCl_3$*) of **4**.

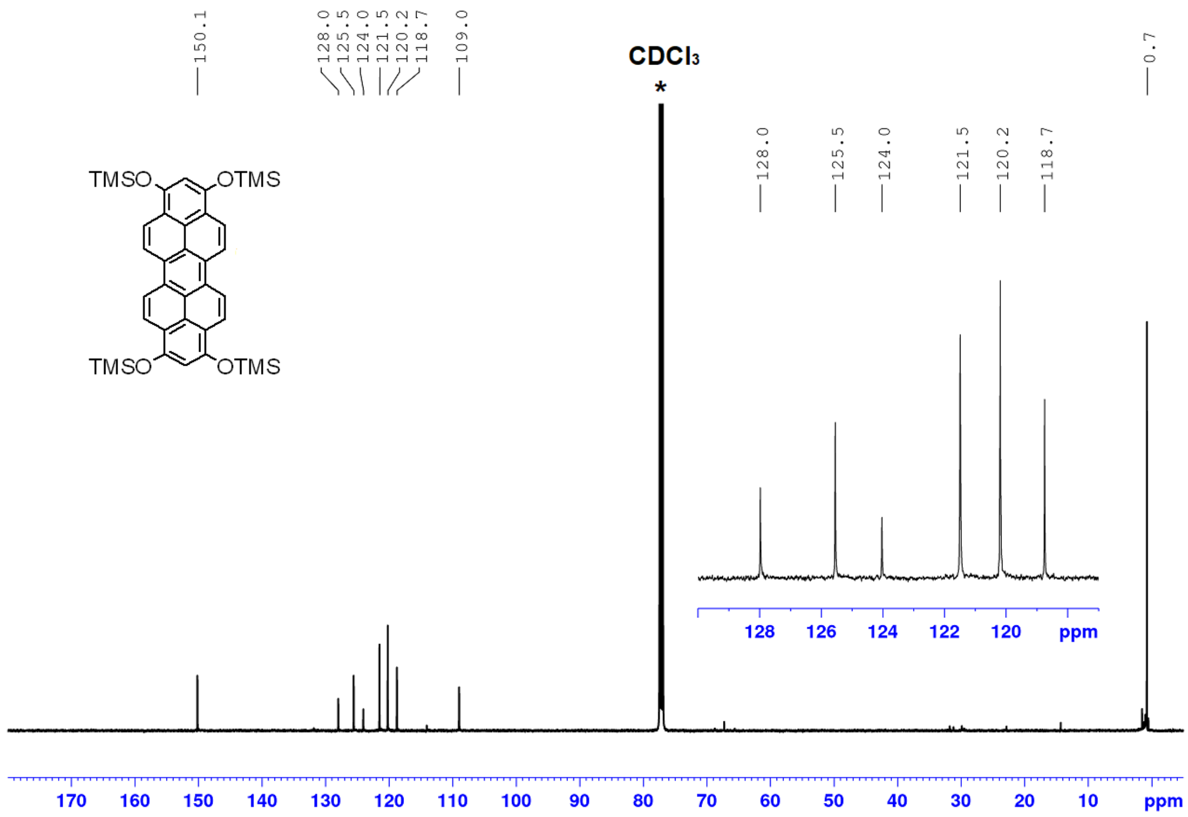

Figure S41. ^{13}C-NMR (125.8 MHz, 298K, $CDCl_3$*) of **4**.

*1,3,10,12-tetrakis((trimethylsilyl)oxy)benzo[rst]dinaphtho[8,1,2-cde:2',1',8'-klm]pentaphene (**13**)*

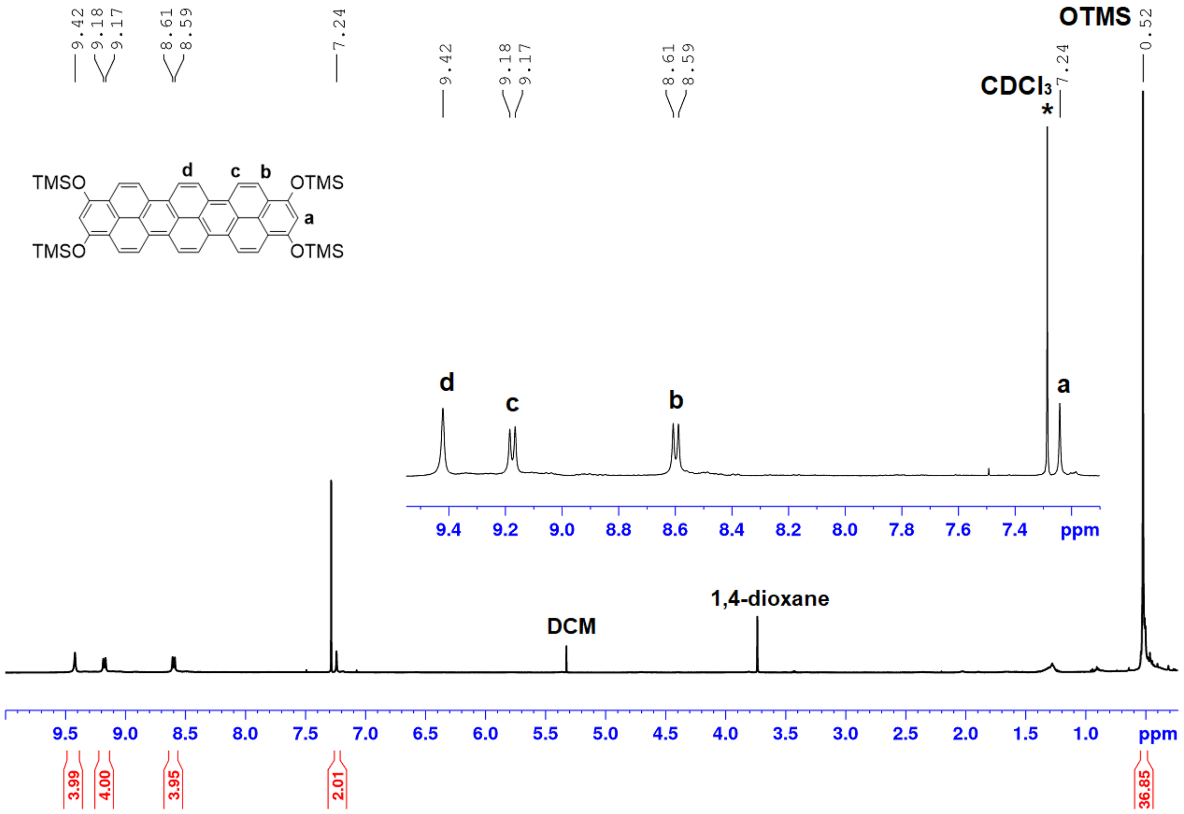

Figure S42. ^{1}H-NMR (500.1 MHz, 298K, $CDCl_3$*) of **13**.

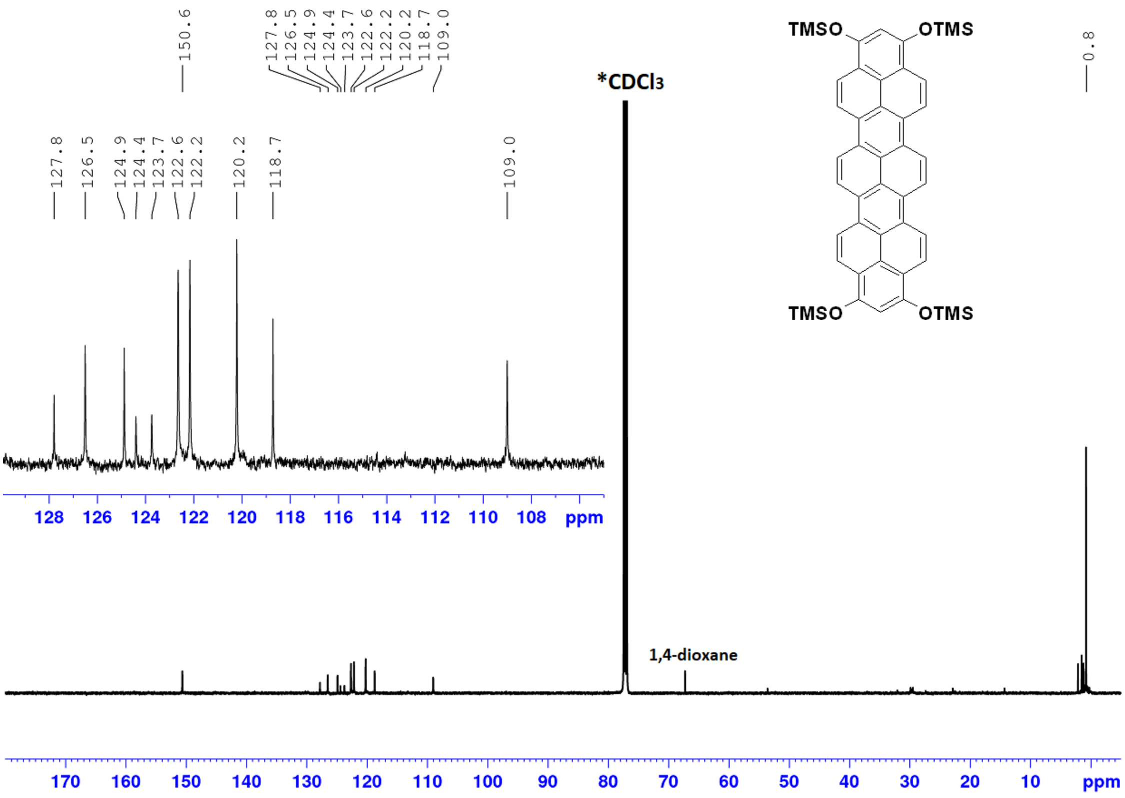

Figure S43. ^{13}C-NMR (125.8 MHz, 298K, $CDCl_3$*) of **13**.

*1,3,10,12-tetrakis((triisopropylsilyl)oxy)benzo[rst]dinaphtho[8,1,2-cde:2',1',8'-klm]pentaphene (**14**)*

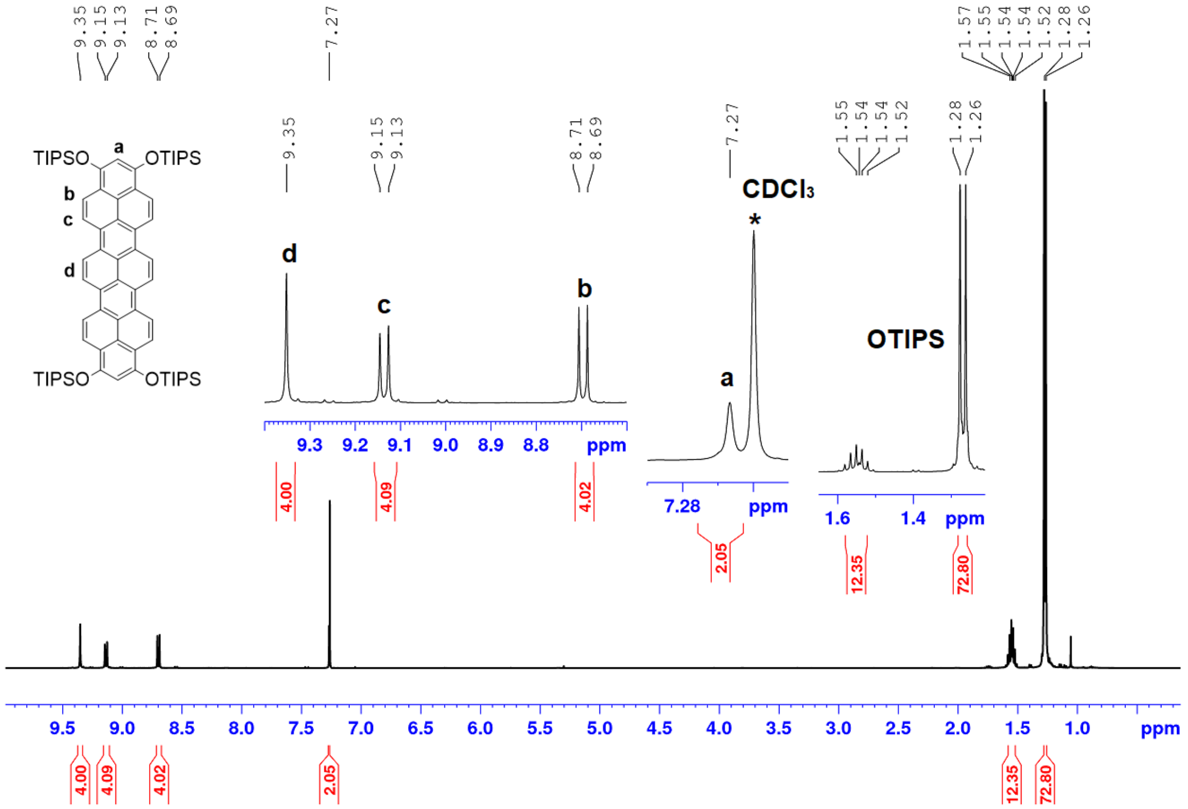

Figure S44. ^{1}H-NMR (500.1 MHz, 298K, $CDCl_3$*) of **14**.

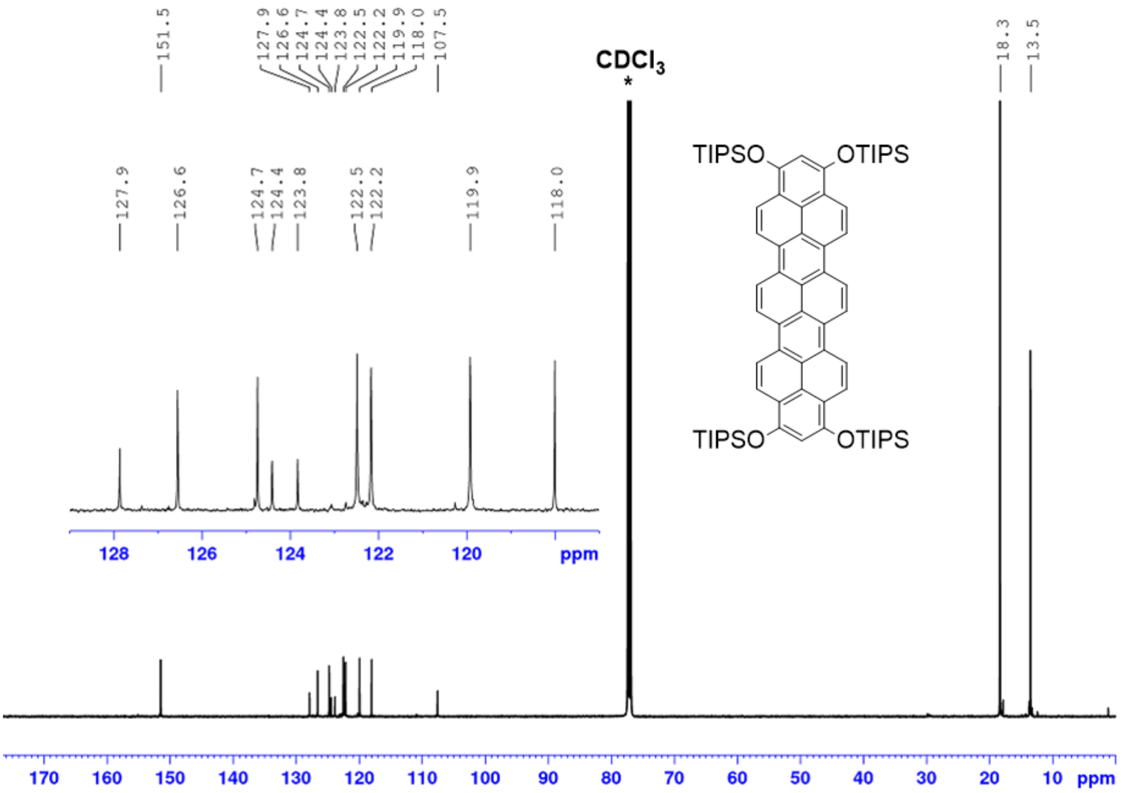

Figure S45. ^{13}C-NMR (125.8 MHz, 298K, $CDCl_3$*) of **14**.

*((2,13-dibutylbenzo[rst]benzo[12,1]tetrapheno[7,8,9-cde]naphtho[2,1,8-klm]pentaphene-1,3,12,14-tetrayl)tetrakis(oxy))-tetrakis(trimethylsilane) (**S4**)*

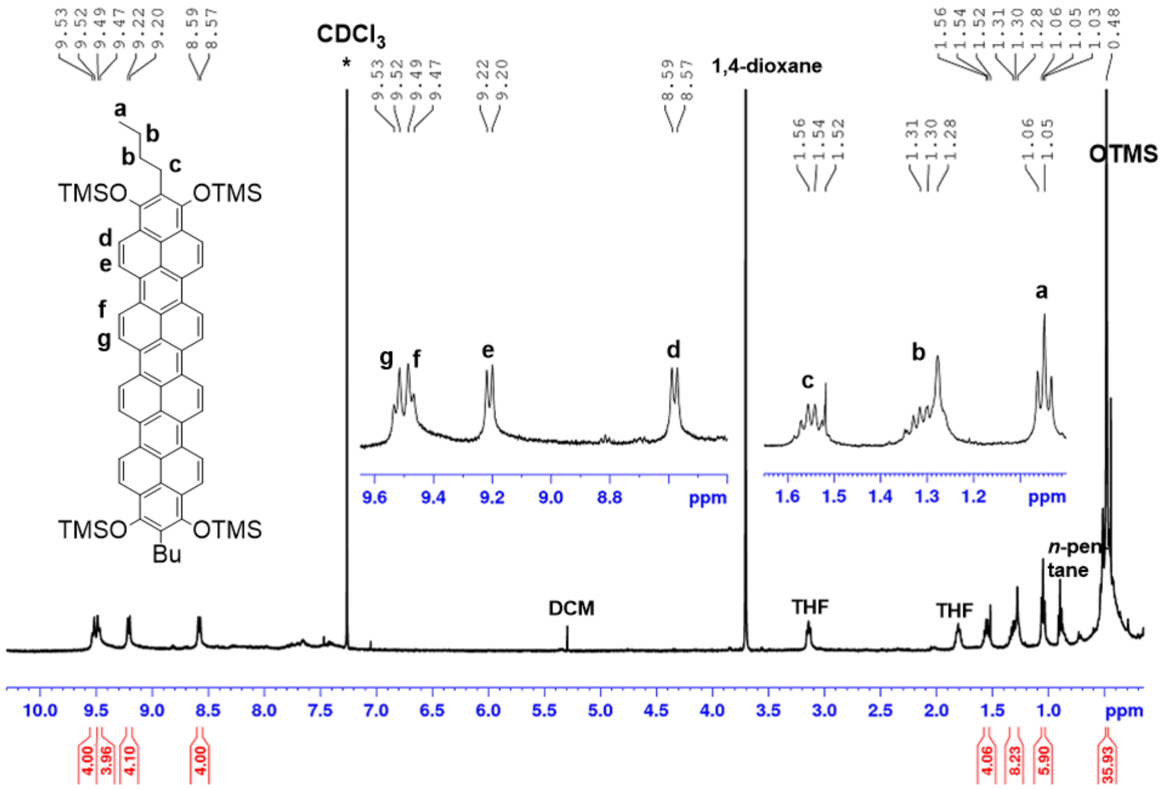

Figure S46. ^{1}H-NMR (500.1 MHz, 323K, $CDCl_3$*) of **S4**.

*1,3,10,12-tetrakis((triisopropylsilyl)oxy)benzo[rst]dinaphtho[8,1,2-cde:2',1',8'-klm]pentaphene (**15**)*

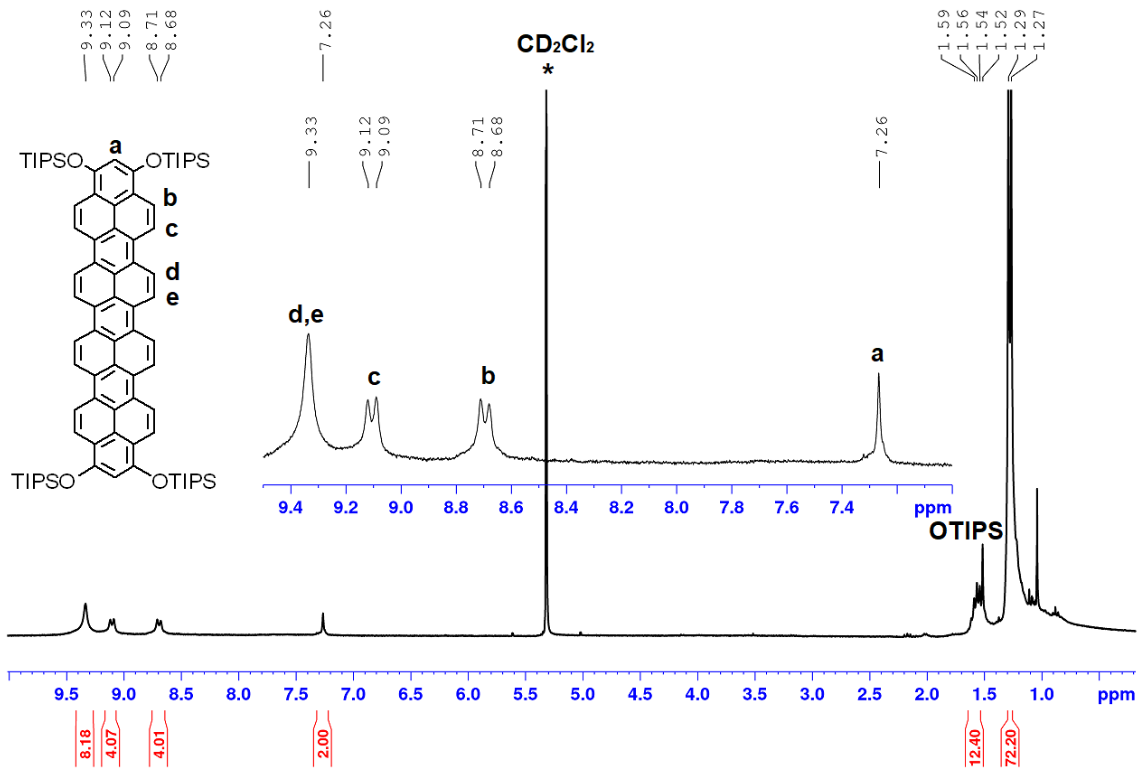

Figure S47. ^{1}H-NMR (300.1 MHz, 298K, CD_2Cl_2*) of **15**.

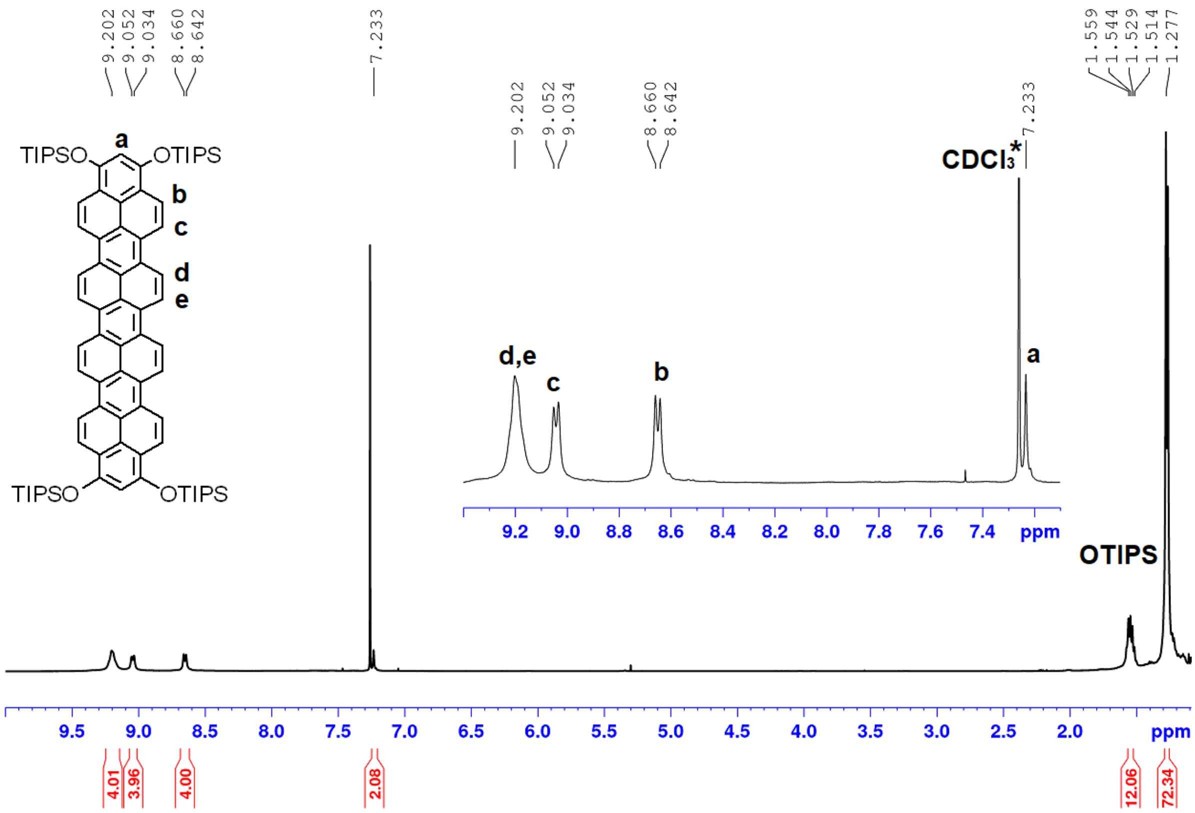

Figure S48. ^{1}H-NMR (500.1 MHz, 298K, $CDCl_3$*) of **15**.

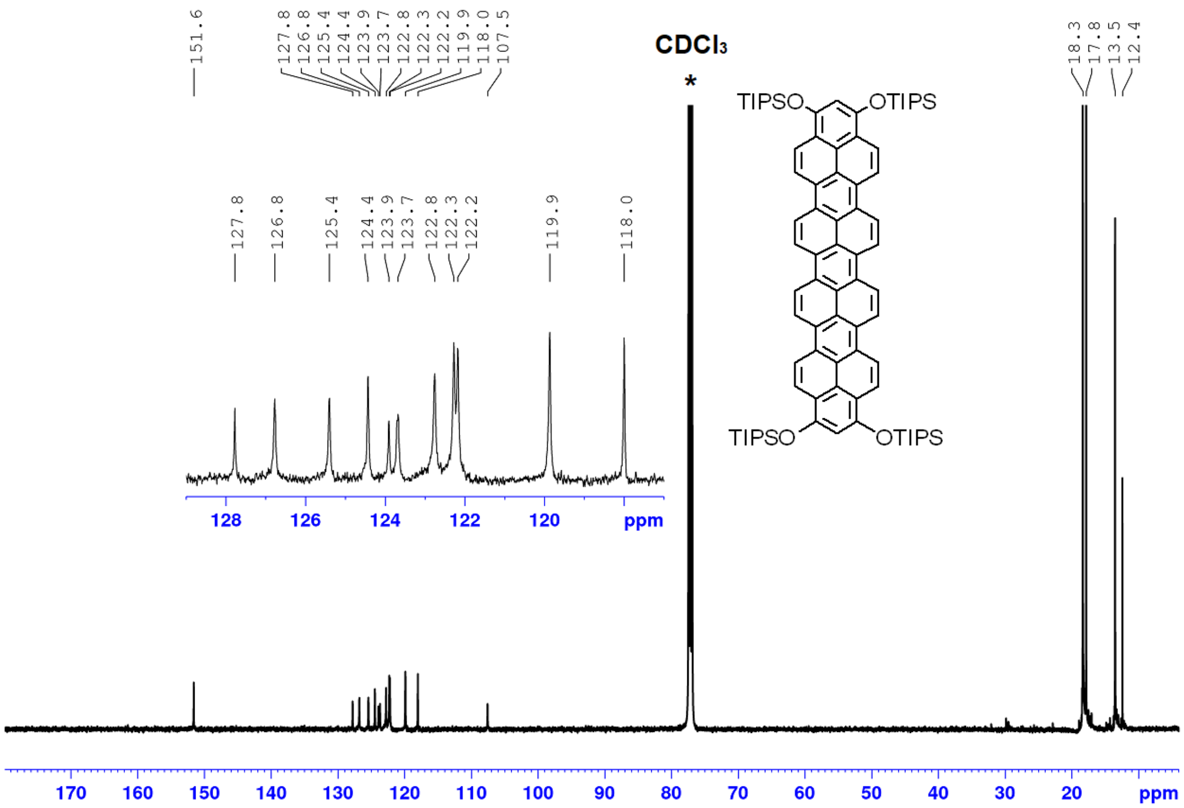

Figure S49. ^{13}C-NMR (125.8 MHz, 298K, $CDCl_3$*) of **15**.

APCI(-) Mass spectra of Suzuki coupling products

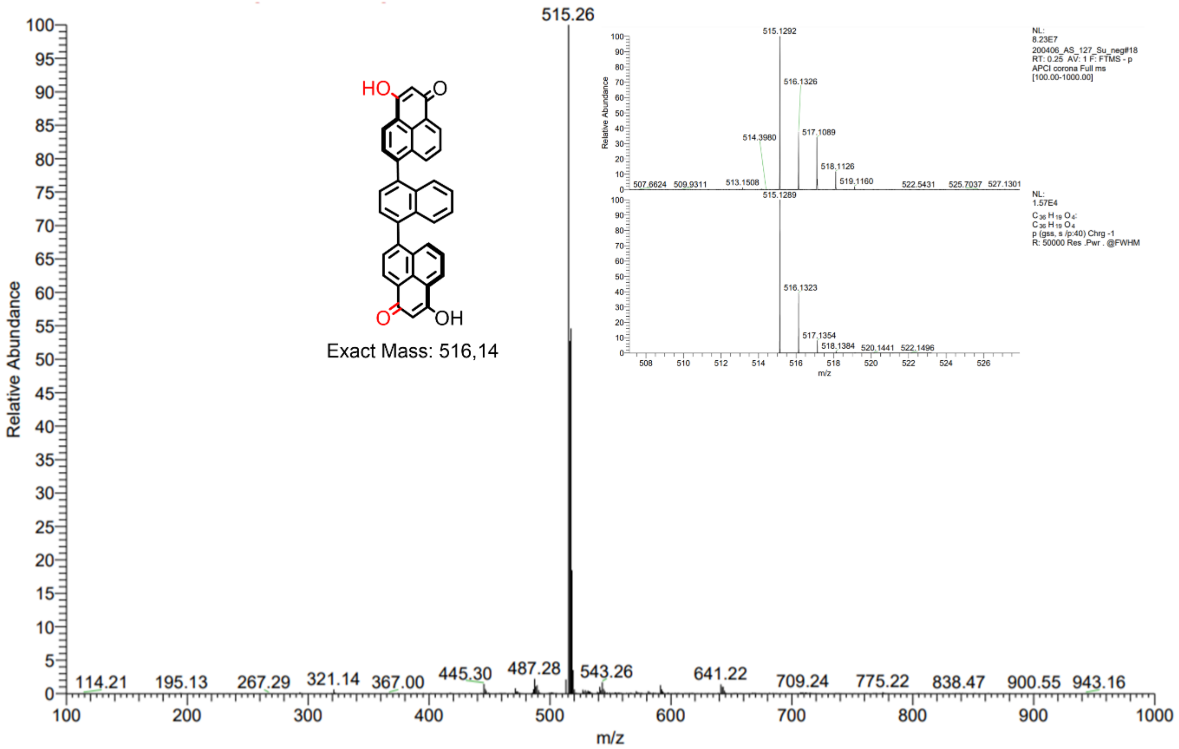

Figure S50. APCI(-) spectrum of **9**. Inset: high resolution spectrum and simulation of the product peak.

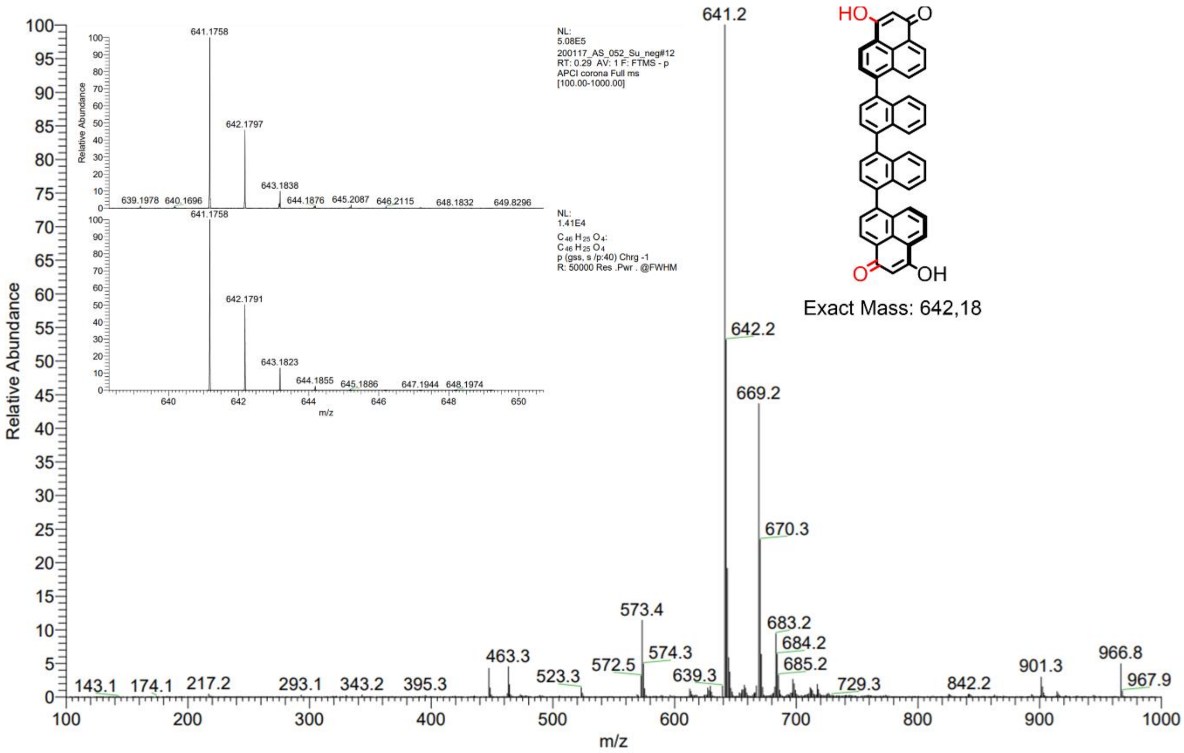

Figure S51. APCI(-) spectrum of **10**. Inset: high resolution spectrum and simulation of the product peak.

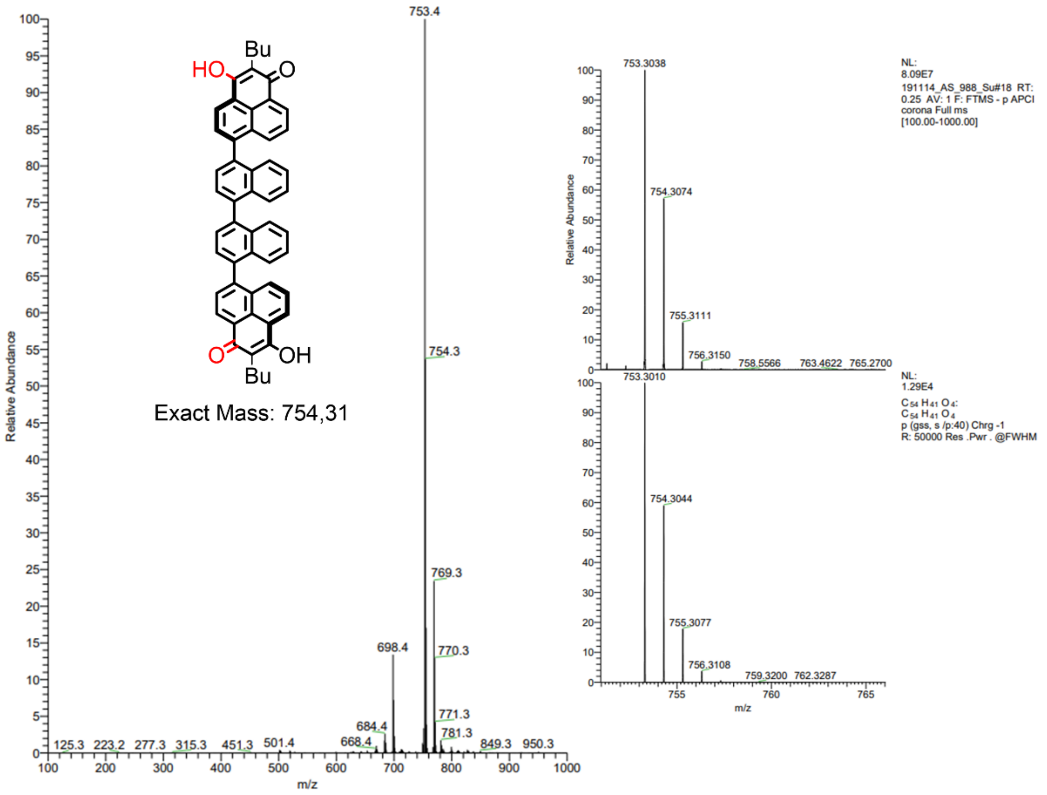

Figure S52. APCI(-) spectrum of **S2**. Inset: high resolution spectrum and simulation of the product peak.

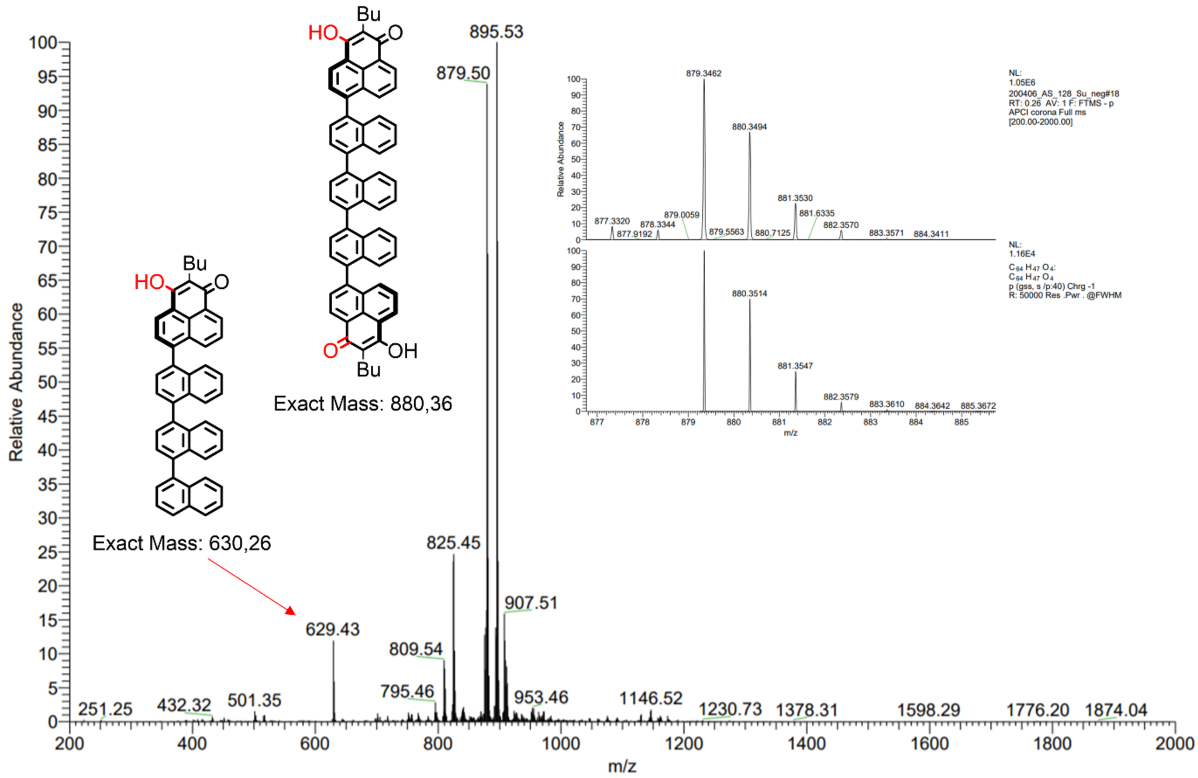

Figure S53. APCI(-) spectrum of **S7**. Inset: high resolution spectrum and simulation of the product peak.

Reverse Phase HPLC traces of Suzuki coupling products

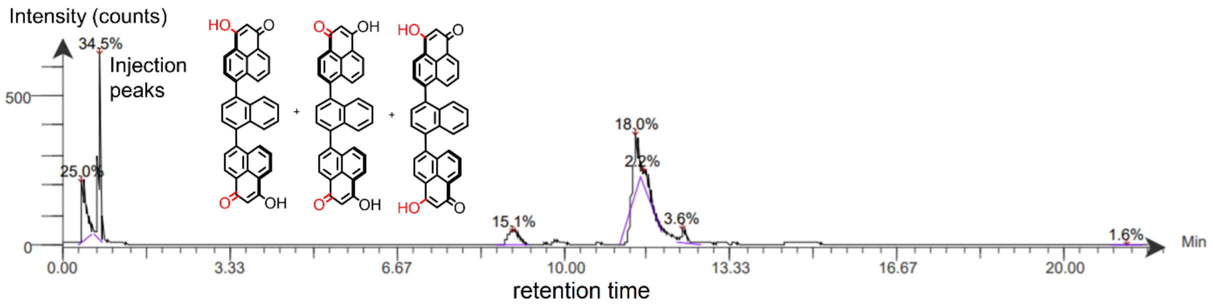

Figure S54. HPLC elution trace of **9** deprotonated with diluted aqueous NaOH (1 M), eluent: H_2O/acetonitrile, 95:5→5:95.

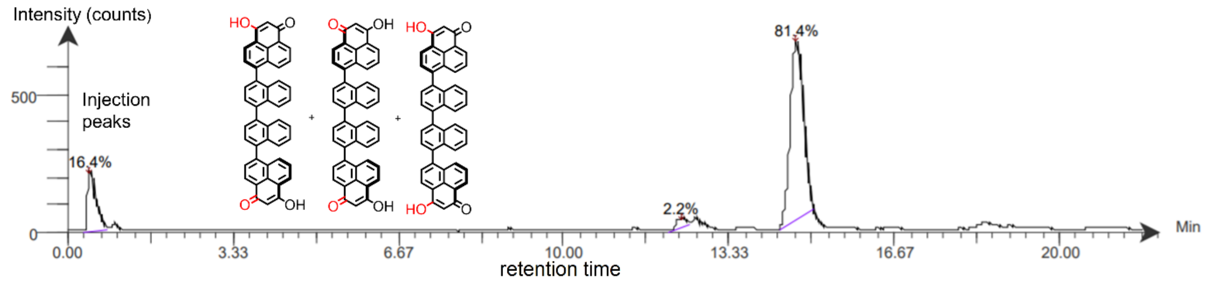

Figure S55. HPLC elution trace of **10** deprotonated with diluted aqueous NaOH (1 M), eluent: H_2O/acetonitrile, 95:5→5:95.

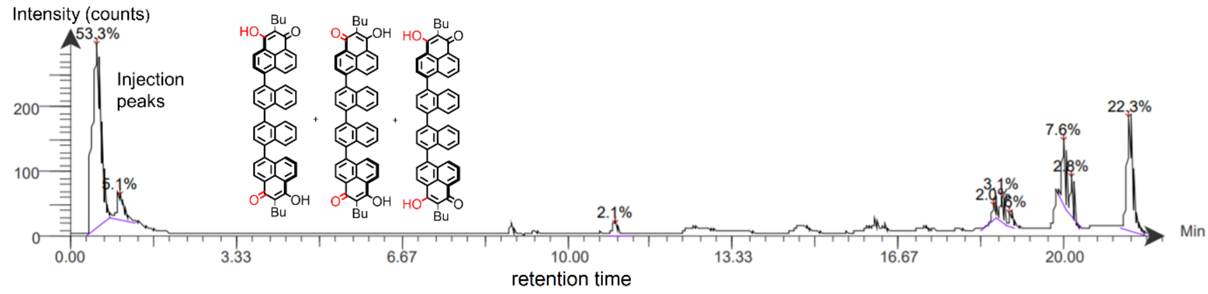

Figure S56. HPLC elution trace of **S2** deprotonated with diluted aqueous NaOH (1 M), eluent: H_2O/acetonitrile, 95:5→5:95.

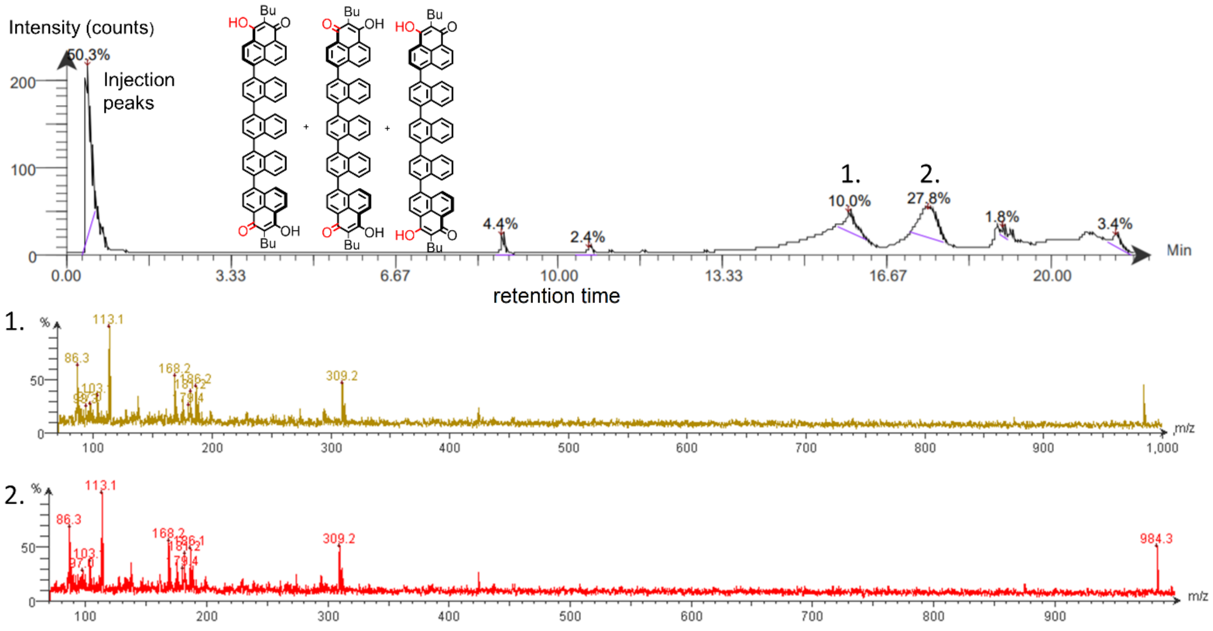

Figure S57. HPLC elution trace of **S7** deprotonated with diluted aqueous NaOH (1 M), eluent: H_2O/acetonitrile, 95:5→5:95 and ESI(-) mass spectra of fractions 1 and 2.

References

[1] W. L. F. Armarego, D. D. Perrin, *Purification of laboratory chemicals*, Butterworth-Heinemann, Oxford, **2002**.
[2] G. M. Sheldrick, *Acta Cryst. C* **2015**, *71*, 3–8.
[3] C. B. Hübschle, G. M. Sheldrick, B. Dittrich, *J. Appl. Cryst.* **2011**, *44*, 1281–1284.
[4] A. L. Spek, *Acta Cryst. D* **2009**, *65*, 148–155.
[5] H. P. K. Brandenburg, *Diamond*, Crystal Impact GbR, Bonn, **2012**.
[6] J. R. Lakowicz, *Principles of fluorescence spectroscopy*, Springer, New York, **2010**.
[7] a) Lee, Yang, Parr, *Phys. Rev. B* **1988**, *37*, 785–789; b) P. J. Stephens, F. J. Devlin, C. S. Ashvar, C. F. Chabalowski, M. J. Frisch, *Faraday Disc.* **1994**, *99*, 103; c) A. D. Becke, *J. Chem. Phys.* **1993**, *98*, 5648–5652.
[8] a) A. Schäfer, C. Huber, R. Ahlrichs, *J. Chem. Phys.* **1994**, *100*, 5829–5835; b) F. Weigend, *Phys. Chem. Chem. Phys.* **2006**, *8*, 1057–1065.
[9] F. Weigend, R. Ahlrichs, *Phys. Chem. Chem. Phys.* **2005**, *7*, 3297–3305.
[10] a) K. Eichkorn, O. Treutler, H. Öhm, M. Häser, R. Ahlrichs, *Chem. Phys. Lett.* **1995**, *240*, 283–290; b) M. von Arnim, R. Ahlrichs, *J. Comput. Chem.* **1998**, *19*, 1746–1757; c) F. Weigend, M. Häser, H. Patzelt, R. Ahlrichs, *Chem. Phys. Lett.* **1998**, *294*, 143–152.
[11] T. Yanai, D. P. Tew, N. C. Handy, *Chem. Phys. Lett.* **2004**, *393*, 51–57.
[12] a) R. Krishnan, J. S. Binkley, R. Seeger, J. A. Pople, *J. Chem. Phys.* **1980**, *72*, 650–654; b) A. D. McLean, G. S. Chandler, *J. Chem. Phys.* **1980**, *72*, 5639–5648.
[13] S. Grimme, J. Antony, S. Ehrlich, H. Krieg, *J. Chem. Phys.* **2010**, *132*, 154104.
[14] a) A. D. Becke, E. R. Johnson, *J. Chem. Phys.* **2005**, *123*, 154101; b) S. Grimme, S. Ehrlich, L. Goerigk, *J. Comput. Chem.* **2011**, *32*, 1456–1465; c) E. R. Johnson, A. D. Becke, *J. Chem. Phys.* **2006**, *124*, 174104; d) E. R. Johnson, A. D. Becke, *J. Chem. Phys.* **2005**, *123*, 24101.
[15] F. Neese, *WIREs Comput Mol Sci* **2011**, *2*, 73–78.
[16] a) Perdew, Burke, Ernzerhof, *Phys. Rev. Lett.* **1996**, *77*, 3865–3868; b) J. P. Perdew, K. Burke, M. Ernzerhof, *Phys. Rev. Lett.* **1997**, *78*, 1396.
[17] F. Weigend, *Phys. Chem. Chem. Phys.* **2002**, *4*, 4285–4291.
[18] D. Alezi, Y. Belmabkhout, M. Suyetin, P. M. Bhatt, Ł. J. Weseliński, V. Solovyeva, K. Adil, I. Spanopoulos, P. N. Trikalitis, A.-H. Emwas et al., *J. Am. Chem. Soc.* **2015**, *137*, 13308–13318.
[19] H. Jia, Y. Gao, Q. Huang, S. Cui, P. Du, *Chem. Commun.* **2018**, *54*, 988–991.
[20] N. Buffet, E. Grelet, H. Bock, *Chem. Eur. J.* **2010**, *16*, 5549–5553.
